# Development and usability testing of tools to facilitate incorporating intersectionality in knowledge translation

**DOI:** 10.1186/s12913-022-08181-1

**Published:** 2022-06-27

**Authors:** Kathryn M. Sibley, Danielle Kasperavicius, Isabel Braganca Rodrigues, Lora Giangregorio, Jenna C. Gibbs, Ian D. Graham, Alison M. Hoens, Christine Kelly, Dianne Lalonde, Julia E. Moore, Matteo Ponzano, Justin Presseau, Sharon E. Straus

**Affiliations:** 1 Department of Community Health Sciences, University of Manitoba, 379 - 753 McDermot Avenue, Winnipeg, MB R3E 0W3 Canada; 2 George and Fay Yee Centre for Healthcare Innovation, 379- 753 McDermot Avenue, Winnipeg, MB R3E 0W3 Canada; 3 Knowledge Translation Program, St. Michael’s Hospital, Unity Health Toronto, Toronto, ON Canada; 4 McMaster University, Department of Medicine, Hamilton, Ontario Canada; 5 Department of Kinesiology and Health Sciences, and Schlegel-UW Research Institute for Aging, University of Waterloo, Waterloo, ON Canada; 6 Department of Kinesiology and Physical Education, McGill University, Montreal, QC Canada; 7 Clinical Epidemiology, Ottawa Hospital Research Institute, Ottawa, ON Canada; 8 School of Epidemiology and Public Health, University of Ottawa, Ottawa, ON Canada; 9 Department of Physical Therapy, University of British Columbia, Vancouver, BC Canada; 10 Learning Network, Centre for Research & Education on Violence Against Women & Children, Western University, London, ON Canada; 11 The Center for Implementation, Toronto, ON Canada; 12 School of Psychology, University of Ottawa, Ottawa, ON Canada; 13 Division of Infectious Diseases, St. Michael’s Hospital, Unity Health Toronto, Toronto, ON Canada; 14 Department of Medicine, University of Toronto, Toronto, ON Canada

**Keywords:** Health equity, Gender-based analysis, Knowledge translation, Integrated knowledge translation

## Abstract

**Background:**

The field of knowledge translation (KT) has been criticized for neglecting contextual and social considerations that influence health equity. Intersectionality, a concept introduced by Black feminist scholars, emphasizes how human experience is shaped by combinations of social factors (e.g., ethnicity, gender) embedded in systemic power structures. Its use has the potential to advance equity considerations in KT. Our objective was to develop and conduct usability testing of tools to support integrating intersectionality in KT through three key phases of KT: identifying the gap; assessing barriers to knowledge use; and selecting, tailoring, and implementing interventions.

**Methods:**

We used an integrated KT approach and assembled an interdisciplinary development committee who drafted tools. We used a mixed methods approach for usability testing with KT intervention developers that included semi-structured interviews and the System Usability Scale (SUS). We calculated an average SUS score for each tool. We coded interview data using the framework method focusing on actionable feedback. The development committee used the feedback to revise tools, which were formatted by a graphic designer.

**Results:**

Nine people working in Canada joined the development committee. They drafted an intersectionality primer and one tool that included recommendations, activities, reflection prompts, and resources for each of the three implementation phases. Thirty-one KT intervention developers from three countries participated in usability testing. They suggested the tools to be shorter, contain more visualizations, and use less jargon. Average SUS scores of the draft tools ranged between 60 and 78/100. The development committee revised and shortened all tools, and added two, one-page summary documents. The final toolkit included six documents.

**Conclusions:**

We developed and evaluated tools to help embed intersectionality considerations in KT. These tools go beyond recommending the use of intersectionality to providing practical guidance on how to do this. Future work should develop guidance for enhancing social justice in intersectionality-enhanced KT.

**Supplementary Information:**

The online version contains supplementary material available at 10.1186/s12913-022-08181-1.

## Contributions to the literature

- This paper describes the development of the first tools for explicitly integrating intersectionality in KT planning. It brings together symbiotic work from two important and historically distinct academic traditions with shared goals of advancing social conditions (which includes health) and justice.
- The findings of the project move beyond recommending the use of intersectionality to providing practical resources on how to do this. The resultant tools may help ensure that KT interventions are inclusive and have the most potential to help a greater proportion of people.

## Background

Complexity and context are recognized as critical for implementation of evidence-informed health services and policies [1, 2]. Among many complex contextual factors, social factors, “the conditions in which people are born, grow, work, live, and age, and the wider set of forces and systems shaping the condition of daily life” [3], are consistently identified as key determinants of health and health inequities [4, 5]. Implementation science, and the field of knowledge translation (KT) more broadly, has been criticized for neglecting social factors such as gender [6] and equity [7] and perpetuating inequities due to foundational evidence that underrepresents key populations such as women and racialized communities [8]. Numerous efforts are underway that address these criticisms, including calls for explicit consideration of equity in every implementation project [9], synthesis of implementation equity effectiveness data, adaptation of conceptual frameworks, and development of guidance to support integrating equity considerations specifically in implementation research and practice [10–12] and more broadly in evidence-based decision-making to address global societal challenges [13].

**Fig. 1:**
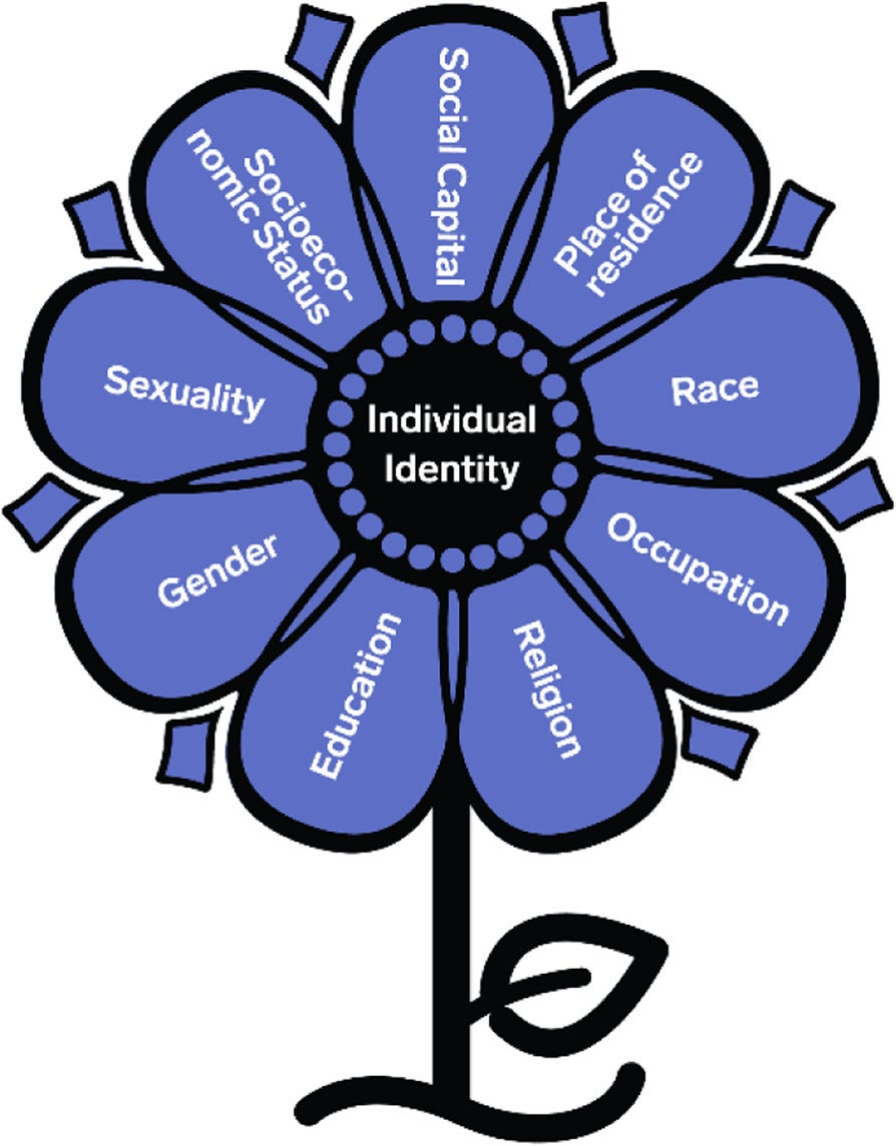
Visual representation of some intersecting categories. The categories mentioned in this figure are not an exhaustive list

Intersectionality is one approach that can advance equity considerations in KT and implementation. Intersectionality, a term coined by Dr. Kimberle Crenshaw [14], is a theoretical approach based on the work of Black feminist and critical race scholars who identified and emphasized how Black women faced multiple and intersecting forms of oppression [15–17]. In the context of health, Hankivsky and colleagues described how intersectionality “focuses on the ways in which multiple axes of social inequality intersect and co-construct one another at the macro and micro levels to produce a broad range of unequal outcomes, in both individual and population health” [18]. Axes of social inequality include (but are not limited to) experiences of racism, classism, heteronormativity, cisnormativity, ageism, audism, and xenophobia. The social intersections inhabited by an individual are not inherently additive [19] and reflect larger systems of privilege and/ or exclusion [20]. As such, intersectionality explains how a person’s experiences of oppression and/ or privilege may shift depending on the specific situation and highlights how these experiences and inequities unfold within complex health and social system structures (Fig. 1).

We propose that taking an intersectional approach may improve the design, delivery, receipt, effectiveness, and impact of KT and implementation interventions by more directly addressing specific health inequities affecting individuals and groups. Conversely, neglecting intersecting social factors in KT and implementation may perpetuate inequities leading to poor health outcomes [21]. For example, an analysis of census data in Ontario, Canada showed that neighbourhoods with a higher concentration of people of colour during the COVID-19 pandemic had three times higher COVID-19 infection rates, four times higher hospitalization rates, and deaths twice as high compared to neighbourhoods with lower concentrations of people of colour [22]. People of colour are more likely to be employed in work most negatively affected by the pandemic (e.g., food services); newcomers are also overrepresented in jobs with greater potential exposure to COVID-19 [22]. Accordingly, newcomers who are people of colour face compounded challenges in health and well-being due to intersections in social determinants of health [22]. If public health strategies do not use an intersectional lens, we risk that these populations will continue to bear the brunt of morbidity and mortality in future COVID-19 waves. However, despite arguments supporting the principles of intersectionality, intersectionality is critiqued for being difficult to apply [23]. Practical guidance and strategies for applying intersectionality in KT and implementation are warranted. As such, our objective in this project was to develop, evaluate and refine a suite of tools to support the application of intersectionality in KT. Our ultimate goal of developing these tools is to increase knowledge, facilitate positive attitudes, and support adoption of intersectional considerations in KT.

## Methods

### Project foundations and preliminary work

This study took place within a series of projects to advance intersectionality considerations in KT [24–28]. The series of projects took place in Canada between 2017 and 2019. A full description of the series of projects is available [27]. The projects used an integrated knowledge translation approach [29], defined here as a collaborative research process in which knowledge users [those who can use the knowledge generated through research to make informed decisions] [30] are engaged in governance and conduct of the research process. In the development phase, the overall project team led by SES identified KT intervention developers as the primary knowledge users for this work, and 19 people with experience developing KT interventions in research or practice roles were engaged as co-investigators or collaborators from project conception. Recognizing the interdisciplinary nature of this work, the project steering committee invited 26 scholars with training in intersectionality, women and gender studies, and health equity as co-investigators or collaborators. These individuals were identified from those who received national research funding or who published in peer-reviewed journals in this area.

The overall project team selected the Knowledge-to-Action (KTA) Framework [31], a highly cited implementation process model [32, 33] developed from a review of over 30 planned action theories, as an overarching framework. To facilitate the process of applying intersectionality in KT, the team chose to narrow our target context to health for older adults to reflect the needs of a population that has growing health care needs and where documented health disparities exist [34–37]. In the first project step, they invited a diverse group of community members, clinicians, KT practitioners, and researchers to participate in a Nominal Group Technique exercise and prioritized phases of KT to address using intersectionality [28]. In the second step, they prioritized the following KTA phases for integrating intersectionality: *identifying the problem/ evidence-to-practice gaps*; *assessing barriers to knowledge use*; and *selecting, tailoring, and implementing interventions* [28]*.* They then used a structured consensus process to identify one model, theory, or framework (MTF) corresponding to each prioritized KTA phase [25]. The following MTFs were selected: Iowa Model of Evidence-Based Practice [38] (*identifying the problem/ evidence-to-practice gaps*), the Consolidated Framework for Implementation Research [39] (*assessing barriers to knowledge use)*, and the Theoretical Domains Framework/ Behaviour Change Wheel [40] (*selecting, tailoring, and implementing interventions).* In the third step, they developed intersectionality-enhanced versions of each selected MTF through an iterative process of co-creation and consensus-building [24, 27].

### Study design

**Fig. 2:**
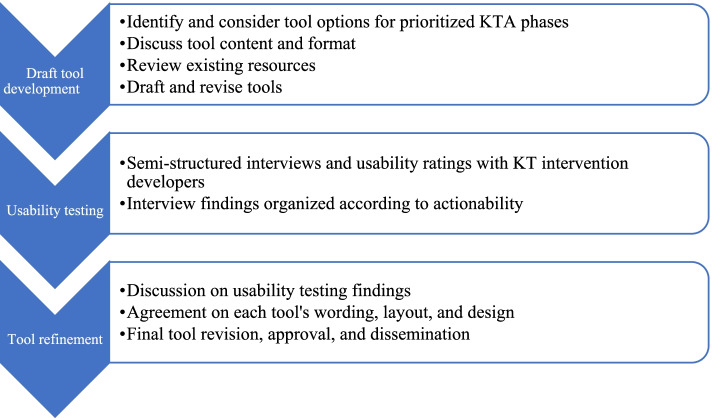
Tool Development Process

We used an integrated knowledge translation approach with an iterative, multi-step process to develop the suite of tools for applying intersectionality in each of the prioritized KTA phases and accompanying intersectionality-enhanced MTFs (Fig. 2). Our integrated knowledge translation approach involved including the project’s primary knowledge users (KT intervention developers) as members of an interdisciplinary committee we established to develop the tools (described below). All committee members were provided the opportunity to meet international standards for authorship of this peer-reviewed manuscript [41].

### Development committee formation and approach

We established an interdisciplinary committee to lead the tool development process. We recruited development committee members through informal networks of existing overall project team members (academic and knowledge user co-investigators and collaborators) and purposive recruitment of intersectionality experts. All committee members, including knowledge users, were involved at a level consistent with *Collaborate* on the IAP2 Spectrum of Public Participation, whereby members partnered “in each aspect of the decision, including the development of alternatives and the identification of the preferred solution” [42].

We created Terms of Reference for the development committee (Appendix A, see in Additional file 1). The committee met on an ad hoc basis via web-based conferencing system. All committee members provided their time in-kind. As intersectionality emphasizes reflexivity as a core concept, the committee worked to model intersectionality practice during each meeting, guided by Weaver’s Long Table Format [43]. For example, in each meeting, each member introduced themselves and specified how they would like to be addressed, what pronouns to use, and why they were interested in the project. Committee members were also encouraged to independently reflect on their personal values, social locations, privileges and impact of all these factors on their work in this project, and to complete the Harvard Implicit Association test [44].

#### Step 1: draft tool development

The development committee members held a series of meetings to develop draft tools. During initial meetings, committee members reviewed background project information and reference materials for each prioritized KTA phase and accompanying intersectionality-enhanced MTF and discussed ideas for scope, content, and structure of the suite of tools. The committee agreed that decisions would be made by consensus when at least 50% of committee members were present; if a decision by consensus was not possible, meeting attendees voted. Committee members identified a short list of tool options, discussed format and content options, and decided on the tool components. Research staff then created draft tools based on the instructions and guidance of the committee. Committee members individually reviewed and suggested revisions for each tool and discussed these revisions at subsequent meetings. This process was repeated until all members were satisfied with the tool drafts and did not request additional revisions. Following each committee discussion, research staff revised draft tools based on committee guidance and input from graphic designers.

#### Step 2: usability testing

We invited individuals who represented the relevant knowledge user group and self-identified as having experience developing KT interventions to participate using a purposive sampling approach via individual emails, recruitment flyers, electronic newsletters, and Twitter. We included individuals professionally employed in KT practice, as well as KT and implementation scientists. We recruited eight to ten individuals to test each tool, consistent with previous qualitative research sampling approaches [45].

Participants reviewed the tool prior to the interview and had access to the tool during the interview (either on screen or paper copy). Interview participants were sent questions in advance. Open-ended questions explored participant perceptions of each tool during one-hour semi-structured interviews. The interview included probes on issues that emerged, including the content and structure of the tools (Appendix B, see in Additional file 2). Feedback was elicited using the “think aloud” method [46]. Specifically, participants described thoughts, questions or concerns that came to mind as they reviewed the tool during the interview and reflected on how they would use it in practice. Interviews were conducted in English by two members of the research team (an interviewer and a note taker). A detailed notetaking chart to track information related to participants’ overall comments and suggestions for change was used during the interview. Each participant was asked to complete the System Usability Scale (SUS), a reliable usability measure [47, 48]. The SUS consists of 10 questions on a 5-point Likert scale, which is interpreted as an overall score between 0 and 100 [47, 48]. A score exceeding 68 is considered above average usability [47]. Interviews were audio recorded.

Immediately after each interview, the interviewer and note taker discussed notes and collated comments to ensure all relevant insights were recorded. If any points were unclear, the team reviewed the audio recording. They reviewed and resolved any conflicts in the notes until no critical issues were identified. They organized data using a framework approach [49]. For each tool, they coded interview findings into four categories based on potential actionability of the feedback: i. no action required (e.g., “this tool will be especially useful for less experienced team members); ii. formatting (e.g., “it would be helpful if key questions were bolded); iii. out of scope & no action taken (e.g., “it would be great if you can make a video explaining different sections of the tool”); and, iv. discussion and decision required (e.g., “is it appropriate to use the term Indigenous ancestry?”). Any feedback related to the substantive content of the tool was coded for discussion and decision. The two staff who conducted interviews coded data independently and resolved conflicts through discussion [49].

#### Step 3: tool refinement

For each tool, research staff created a summary report of the interview feedback and presented it to the development committee. To facilitate review and discussion, staff identified related or commonly mentioned issues and organized them into groups. Committee members individually reviewed summary reports, then met to address the discussion feedback; grouped issues were summarized in slides and discussed by the committee. As with the previous phase, decisions were made when at least 50% of meeting attendees voted in favor of a decision. When the committee did not meet the threshold for a decision, comments were forwarded to the principal investigator (SES, also a knowledge user) for final decision. Research support staff then revised tool content based on committee guidance and a copy editor and graphic designer finalized each tool’s wording, formatting, and layout.

## Results

### Development committee structure and processes

Eleven individuals joined the development committee; however, two individuals withdrew before the first meeting due to other commitments. Committee members included a subset of project co-investigators (*n* = 3) and members identified through external recruitment (*n* = 6). The development committee consisted of scholars with training in women and gender studies, political science and intersectionality, adult education experts, KT research funders, KT practitioners and KT and implementation researchers. Most individuals on the 9-member committee identified as white, heterosexual, female, had no spiritual or religious affiliation, were married or cohabitating, spoke English as a first language, had a masters or doctoral degree, lived in a medium or large population centre, in their own home, had full-time employment, and an annual income > 90,000$ CAD. The committee members were distributed across 4 out of 13 Canadian provinces. Six individuals represented the relevant knowledge user group of KT intervention developers (five as practitioners, one as researcher). Three committee members chose to be involved in manuscript writing and are included as co-authors.

### Draft tool development

The committee held twelve meetings over 1 year during the tool development phase. Initial discussions addressed communication practices and reflections on self-disclosed diversity within the development committee. The committee acknowledged that limited intersecting social categories were represented on the committee. Committee members also emphasized the need for practical tools and to “not reinvent the wheel” and suggested highlighting existing intersectionality resources and references. The committee supported the use of case studies or examples within each tool in addition to reflection questions and intersectionality considerations. Committee members recommended the need for a tool that introduced intersectionality, recognizing that many KT intervention developers may be unfamiliar with the foundations of intersectionality. An early decision of the committee was to focus on the three KTA phases, and include the intersectionality-enhanced MTFs as companions for each phase. The committee made this decision in recognition of the overall guiding framework for the project and the need for practical resources.

Based on these initial discussions and decisions (detailed in Appendix C, see in Additional file 3), the committee developed drafts for four distinct and related tools: an introductory primer on intersectionality (“Intersectionality Guide”) and one tool to support each of the three prioritized KTA phases. For each tool, two to three major revisions were undertaken to produce a draft tool that went forward to usability testing. For the phase *Identifying the problem/ evidence-to-practice gap,* the committee created a “Reflection Workbook” tool to prompt users to reflect on key principles and issues of intersectionality and to consider how that influences KT practice decisions. For the phase *Assessing barriers to knowledge use*, the committee created a “Guide to Common Approaches for Assessing Barriers and Facilitators” that focused on prompts and approaches for including intersectional considerations within data collection methods. For the phase *Selecting, tailoring and implementing interventions,* the committee created a workbook to support a structured approach to developing and conducting KT interventions with an intersectional lens. Draft tools ranged in length from 10 to 25 pages and contained case studies, intersectionality reflection questions, and resources on intersectionality and implementation.

### Usability testing

Thirty-one individuals with experience developing KT interventions participated in usability testing (*n* = 7–9 people/ tool). Characteristics for 27 participants are described in Table 1. The intervention developers had an average of 9 years of KT experience working across three different countries.

**Table 1:** Usability testing participant characteristics

Characteristic	Proportion of sample
Having a longstanding impairment, health condition, or learning difference	11%
White (North American, European)	63%
Average household income greater than $150,000 CAD	33%
LGBTQ+	15%
Sex assigned at birth – female	85%
Current gender identity – female	85%
Have a religious or spiritual affiliation	48%
Primary caregiver of a child or other person	37%
Between 30 and 39 years old	40%

Across the four draft tools, 22 issues were coded as needing discussion and a decision by the development committee (average of 6/ tool). All issues are reported in Appendix D (column A) (see in Additional file 4), and here we summarize issues noted across all tools and highlight issues that stimulated significant discussion and revision by the committee.

Consistent feedback received across all tools was the need to shorten length and include more visualizations. There were also multiple requests for additional information, resources and suggestions for further elaboration. There were numerous comments to clarify and simplify the language level and terms used in the tools, which several participants felt contained unnecessary jargon. Participants requested clear reporting about decisions made (e.g., rationale for use of specific definitions) and underlying assumptions for the tools (e.g., expectation that user has some existing knowledge of KT practice).

There were several issues that subsequently sparked significant committee discussion. Several participants commented on the use of specific terms (e.g., privilege, disadvantage, oppression) and debated whether they might serve to engage the intended knowledge user audience, or alternatively, result in a negative reaction that may discourage some participants from using the tools. Participants raised the specific issue of privilege and emphasized the need to ensure the tools do not further exclude individuals who experience oppression. Participants also commented on the intersectional considerations emphasized in the tools. For example, the importance of considering Indigenous ancestry in the context of intersectionality was raised.

Specific feedback was received on the tool for the *Selecting, tailoring, and implementing interventions* phase. In particular, participants felt that the tool was overwhelming due to the significant amount of content. Participants felt that intersectionality considerations were most relevant in the selecting and tailoring implementation interventions sub-components of the phase and recommended removing the section focused on implementing interventions because it did not contain information specific to intersectionality.

Average SUS scores are summarized in Table 2. Only one tool draft was considered above average in terms of usability (Guide for Common Approaches to Assessing Barriers & Facilitators to Knowledge Use). The remaining tool drafts received a below average usability rating.

**Table 2:** Average System Usability Scale (SUS) summary scores for each draft tool component

Tool Component	Number of Usability Testing Participants	Average SUS Summary Score	Standard Deviation
Introductory Primer (“Intersectionality Guide”)	9	62	18.56
Identify Problem/ Evidence-to-Practice Gap (“Reflection Workbook”)	7	65	23.95
Assess Barriers to Knowledge Use (“Guide for Common Approaches to Assessing Barriers & Facilitators to Knowledge Use”)	8	78	17.75
Selecting, Tailoring and Implementing Interventions (“KT Interventions Workbook”)	7	60	15.30

### Tool refinement

The development committee met four times to address the feedback. The committee reduced the length of individual tools and discussed specific strategies for shortening each tool (e.g. moving some content to appendices, refining table of contents). The committee created short (one-two pages) summaries of longer tools (i.e., those more than ten pages). A statement declaring project assumptions and incoming biases was added to the introduction of each tool. A statement was also created on Indigeneity and the limitations of project considerations and incorporated into each tool. Tool components considered to potentially oppress some individuals further (e.g., social identities statements activity) were included as optional resources/ references only or removed.

The committee discussed the recommendation to remove the “implement intervention” component from the “select, tailor, implement” tool at length, recognizing that this represented a distinct shift from the KTA Framework on which the project was based. The committee decided to move the “implement” content to an appendix to simplify and facilitate deeper consideration of intersectionality in selecting and tailoring KT interventions.

The final suite of tools included six documents: i) Intersectionality Guide; ii) Intersectionality and Knowledge Translation Guide Sheet; iii) Reflection Workbook; iv) Reflection Worksheet; v) Guide for Common Approaches to Assessing Barriers & Facilitators to Knowledge Use; vi) Selecting and Tailoring KT Interventions Workbook [27]. Details of the tools are described in Supplementary Table 1 and all tools are available in Appendix E.

## Discussion

We used a collaborative, reflective, comprehensive and rigorous approach to create a suite of tools to support the development of KT interventions that consider intersectionality. Our collaborative and reflective approach required us to be both responsive and iterative. For example, the development committee chose to focus the tools on each prioritized KTA phase rather than the associated intersectionality-enhanced MTFs. We see this focus as a strength that increases tools’ applicability because users are not limited to using the resources with a specific MTF. A second key point of response was the development committee’s decision to revise the focus of one tool on selecting and tailoring implementation interventions, reducing the emphasis on implementation of interventions, in response to usability test feedback.

Our comprehensive and rigorous approach is consistent with other tool development methods reported in implementation science [50, 51]. A framework for implementation tool development published after this project was launched emphasizes several components included in our development process, such as explore (define), develop, and integrated knowledge translation. We are also addressing evaluation and action planning through our ongoing work evaluating the effects of the tools when applied in an established KT training program. Our tools are distinguished from existing intersectionality guides [52, 53] in two important ways: first, the explicit focus on incorporating intersectionality in KT and implementation research and practice; and second, the explicit reporting of our methodology and approach for tool development. While other intersectionality guides have been identified as using a collaborative approach [52], few (if any) details are reported. There may be different standards for development and reporting for guides across disciplines, and while recognized as important in implementation science, it should not be viewed as a limitation of other guides. An important strength of this project is our interdisciplinary approach, in which we are among the first to bring together people working in both intersectionality and knowledge translation. Team and committee members addressed different foundational knowledge bases, biases and interpretations and were able to come together to produce the tools. This process was not straightforward, and we discuss in a companion paper the challenges, opportunities and tensions we worked through as a team [26].

We recognize the limitations of tools and the series of projects as a whole. Our overall project does not address all components of intersectionality equally. Prior to the current phase of tool development, the overall project team decided to focus on increasing consideration of intersecting social factors in KT, prioritizing this as an appropriate “first step” for our target knowledge user group (KT intervention developers). Other important features of intersectionality, in particular, power, privilege and social justice, were addressed through an embedded approach within the tools. We acknowledge known criticisms of our type of approach. Rice and colleagues argued that “it does intellectual disservice to coopt the parts of a theory that are compatible with existing structures and power relations (for the technical work the theory does in managing complexity) while ignoring aspects of the theory that orient to intervening in those structures and relations to liberatory ends” [23]. The overall project team’s decision to focus on intersecting social considerations may relate in part to the historical traditions of implementation science that do not explicitly consider social justice [54], and may reflect implicit biases towards aspects of intersectionality that are more congruent with implementation conventions. We maintain that even this introductory approach to intersectionality has disruptive potential for KT and implementation science. We recognize that while it is not always necessary to always address all intersections, it can be challenging to determine which components are relevant to the topic at hand, and thus tools need to support the full complement of intersectionality. We will continue to address this limitation in future work, and in the interim refer readers to complementary intersectionality guides with a stronger focus on advocacy and social justice [26].

A second important limitation of our work was that despite our efforts to engage diverse perspectives, not all voices are represented. We recognize that those involved in the project represent a limited range of privileged identities. We acknowledge that our tools were developed with mostly Canadian input, and entirely from high income countries. We recognize that we did not address Indigeneity, which is important in light of Canada’s colonial history (and relevant around the world). We address this limitation in detail on our project website [55]. The limited extent of Indigenous considerations has also been recognized in other intersectionality tools [56], which may reflect ongoing debates about the challenges associated with talking about Indigeneity using intersectionality, as there are questions as to whether this framework is appropriate for addressing colonial legacies and Indigenous values [57, 58]. Lastly, we recognize our methodological limitations whereby we only conducted a single round of usability testing due to financial constraints, and that we did not transcribe interviews due to budget limitations. However, interviews were recorded and reviewed by two staff members to identify salient data.

## Conclusions

Our work makes important advancements to KT. The project as a whole has advanced explicit theoretical considerations of intersectionality in established frameworks, and in KT intervention planning. We developed practical tools that can be used by KT intervention developers to embed intersectionality into KT initiatives, going beyond recommending the use of intersectionality to providing practical guidance on how to do this. Our tools may help ensure that KT planning and resultant interventions are inclusive and have the most potential to help the most people. We invite readers to use the tools and report on their experiences with them so that we can learn, improve future iterations, and examine their effectiveness.

## Supplementary Information

**Additional file 1: Appendix A.** Development Committee Terms of Reference.

**Additional file 2:  Appendix B.** Example Usability Testing Interview Guide (Intersectionality Guide).

**Additional file 3: Appendix C.** Development committee discussions and decisions for tool drafts.

**Additional file 4: Appendix D.** Usability testing issues for development committee discussion and decisions.

**Additional file 5:** Final intersectionality and knowledge translation tools.

**Additional file 6: Supplementary Table 1.** Summary of intersectionality and knowledge translation toolkit.

## Data Availability

The datasets used and analyzed during the current study are available from the corresponding author on reasonable request.

## Abbreviations

KTA: Knowledge-to-Action
KT: Knowledge Translation
MTF: Model, Theory, or Framework
SUS: System Usability Scale
