# Supplementary material for: Development and usability testing of tools to facilitate incorporating intersectionality in knowledge translation
Source: BMC Health Serv Res. 2022 Jun 27;22:830. doi: 10.1186/s12913-022-08181-1 (PMC9238081; doi:10.1186/s12913-022-08181-1)
Supplement: Supplementary file 1 — Additional file 1: Appendix A. Development Committee Terms of Reference. [file 12913_2022_8181_MOESM1_ESM.docx]

Appendix A: Development Committee Terms of Reference

**What is the objective of this study?**

The objective of this research study is for KT intervention developers who are addressing the needs of older adults to use an intersectional approach when designing and implementing interventions.

We define an intersectional approach as a way of thinking that considers the layered and shifting effects of social factors for individuals implementing or receiving a KT intervention.

Examples of social factors are: place of residence, ethnicity, occupation, gender, sexuality, religion, education, socioeconomic status, (dis)ability, d/Deafness, and social capital.

Individuals face intersecting oppressions due to sexism, racism, ableism, classism, transphobia and other forms of oppression. Individuals can simultaneously experience advantage and disadvantage.

**What is the role of the Development Committee?**

- Create and provide feedback on tools or training material for 3 intersectionality-enhanced KT models/theories/frameworks to support their use by KT intervention developers
  - 1-2 tools or training materials for each enhanced KT framework
    - Examples of framework-related tools: interview guides and surveys
    - Examples of framework-related training materials: presentation slides and interactive group activities

**What is the role of the Development Committee Chair?**

The role of the Development Committee Chair is to facilitate each meeting by doing the following:

- Keep meeting discussions in line with the agenda
- Ensure agenda items are discussed and decisions are made
- Encourage participation from Development Committee members
- Summarize action items at conclusion of the meeting

**What are members’ roles and responsibilities?**

The Development Committee will include 9-12 members, each of whom will have a term of membership of 3 years. The vast majority of work will take place in winter 2018/2019. Members will offer diverse knowledge and experiences, and will represent perspectives from KT, adult education, and intersectionality.

Role of Each Member

- Share knowledge, opinions, and experience to help create tools and training materials
- Provide strategic input on the development of model/theory/framework-related tools and training materials

Responsibilities of Members

- Listen to and respect diverse perspectives, forms of knowledge, and lived experiences
- Commit to working through any communication challenges with other Development Committee members
- Listen respectfully and explain opposing ideas
- Consider opposing ideas through an open and critical lens
- Be receptive to receiving and giving feedback (informally to Project Support Team; formally via evaluation methods)
- Actively participate in meetings
- Clarify concepts and ask questions if further information is needed
- Understand the objectives of the research project and desired outcomes
- Be understanding of any limitations of the project and activities that may be out of scope

**How will Meetings Occur?**

Meetings will occur approximately bi-weekly by webinar or teleconference for 1 hour. The meetings will occur from the months of December 2018 to March 2019. If a rapid response is needed to address specific issues, the Development Committee will meet by telephone as needed.

Prior to the meeting, all members will receive a package of materials that contains the meeting agenda, meeting minutes from the previous meeting (if relevant), and any relevant documents that may be discussed at the meeting.

Scheduling meetings

A Doodle poll or email will be sent to all Committee members to determine the most appropriate date and time for the following meeting. We will need at least 50% of attendees at each meeting in order to make project decisions.

Decision-making

The Committee endeavors to arrive at decisions by consensus.

Where a decision cannot be reached by consensus, the decision shall be made by a majority of the votes cast by all members present.

We may also use group consensus processes (e.g., DeBono’s Thinking Hats) to discuss any particularly challenging issues.

Privacy and Confidentiality

All files and information will be kept strictly confidential and will not be used for any purpose outside of that agreed upon by the group members involved in the project.

**How can I give or receive feedback about my participation?**

Committee members will be invited to complete a survey at the end of each meeting to determine meeting effectiveness and member commitment. We want to ensure that the Development Committee membership continues to be productive and profitable for all members.

**How do I withdraw my participation?**

If members would like to withdraw their participation from this Committee, they may do so by approaching the Development Committee Chair and project support team. Members will not be penalized for withdrawing their participation. Members are encouraged to voice their concerns to the Development Committee Chair and project support team at any time, or by completing the feedback survey.
