# Supplementary material for: Development and usability testing of tools to facilitate incorporating intersectionality in knowledge translation
Source: BMC Health Serv Res. 2022 Jun 27;22:830. doi: 10.1186/s12913-022-08181-1 (PMC9238081; doi:10.1186/s12913-022-08181-1)
Supplement: Supplementary file 2 — Additional file 2: Appendix B. Example Usability Testing Interview Guide (Intersectionality Guide). [file 12913_2022_8181_MOESM2_ESM.docx]

Appendix B- Example Usability Testing Interview Guide (Intersectionality Guide)

**Preamble:**

**Welcome and Introductions:**

- Hello, --------.
- My name is [*Research team member*] and I am from the Knowledge Translation program at St. Michael’s Hospital. I will be conducting the interview with you today. In the room with me today, we also have [*Research team member*] our research assistant who will be taking notes in the interview.
- Thank you for agreeing to speak with me today.
- Before we get started with recording the interview, I want to give some background on the project and review any questions on the forms that were sent to you.
  - Once I’ve had a chance to answer all your questions, we will turn on the recorder and begin our interview.

**Explain the Objective of the Project:**

- KT is the process of getting research evidence used in health care practice
- Overall, our objective for this project is for KT intervention developers to use an intersectional approach when designing and implementing KT interventions.
- In particular, the project is focused on KT projects addressing the needs of older adults, though an intersectional approach can apply to any project.
- In order to support KT intervention developers in taking an intersectional approach, an interdisciplinary group has created a few tools that relate to particular parts of the KT process
- The purpose of the interview is to gain your feedback on one of the tools.
  - The tool we will be discussing relates to the ____ stage of the Knowledge to Action cycle, in which:

| Prioritized stage of the [Knowledge to Action Cycle](about:blank) (completed at January 2018 symposium) | Tool created by Development Committee to support an intersectional approach at relevant [Knowledge-to-Action Cycle](about:blank) stage | Key activities in [Knowledge to Action Cycle](about:blank) Stage | Model/Theory/Framework selected and enhanced with intersectionality by the Framework Committee |
| --- | --- | --- | --- |
| N/A | Intersectionality Primer | N/A | N/A |
| Stage 1: Identify Problem; Determine the Know/Do Gap; Identify, Review, Select Knowledge | Problem Identification Reflection Worksheet | •             Define the gap between research and health care.  •             Identify a behaviour change that needs to be made to improve health care (e.g., prescribe a different dosage of a medication, do not screen for a disease using a certain technology).   - This behaviour change is sometimes called a ‘clinical intervention’. | Iowa Model of Research-Based Practice to Promote Quality Care (Iowa Model) |
| Stage 3: Assess Barriers/Facilitators to Knowledge Use | Intersectionality Considerations for Popular Methods for Conducting Barriers/Facilitator Assessments | •             Look for what might help or hinder people from changing their behaviour (i.e., what prevents people from doing or not doing the ‘clinical intervention’) | Consolidated Framework for Implementation Research (CFIR) |
| Stage 4: Select, Tailor, Implement [KT] Interventions | Selecting and Tailoring KT Interventions Worksheet | •             Pick KT strategies that will encourage people to change (e.g., education sessions, use of opinion leaders, policy changes)  •             Strategies should directly address identified barriers/facilitators (e.g., if knowledge is a barrier to doing the ‘clinical intervention,’ a reasonable KT strategy may be education sessions). | Behaviour Change Wheel (BCW)  [includes the Theoretical Domains Framework] |

**Review Terms of Consent and Confidentiality:**

Prior to this interview, you had filled out a study reimbursement form and a confidentiality form. Do you have any questions about the forms you filled in?

*[Address any questions or concerns and remind anyone who has not submitted to send the completed forms back as soon as possible]*

- We take the issue of confidentiality seriously.
- No personal information about you will be shared with anyone outside the study team.
- Your real name will not appear anywhere in the written transcripts or reports concerning today’s session.
- Any information from today that can identify who you are will be changed in any reports coming from this study.
  - For example, if you say your workplace name, we will replace that with a vague identifier such as ‘participant’s workplace’ so the information cannot be linked to you.
  - We will be audio taping this interview so that we do not lose any details of our discussion.

Do I have your permission to audio tape this interview?

*[Turn recorder on]*

Today is (Day, Month, Year) and I’m here with participant _____ conducting the Intersectionality & KT tool usability interview

**For the purpose of recording your consent to participate I will ask you again, do you consent to being interviewed and audio recorded?**

Thank you.

**Interview Questions:**

- For the purpose of this interview, please feel free to scroll through the document online and use your cursor to point to particular sections.
- I’m going to first ask you some questions on the overall tool and then I am going to ask some questions about content on particular questions.
- There are no right or wrong answers.

Have you had a chance to take a look at the tool?

- Great!

OR

- Please take a few minutes to review this tool. Let me know when you are ready.

| **Overall Impressions** |
| --- |
| 1. What are your initial thoughts on this [tool]?  - Do you like this tool? Why or why not? - How could the tool be improved? |
| **Purpose of the Tool** |
| 1. Would this tool help you in planning your KT interventions? 2. Are there any particular parts of the tool that you feel would help more with approaching your projects with an intersectional approach?    - If so, what? 3. Are there any particular parts of the tool that you feel would cause you any concerns and/or that would make it hard for you to include an intersectional approach in your intervention?   If so, what? |
| **Appearance** |
| 1. How did you find the look of this tool?  - i.e., is the tool attractive to look at?  1. Would you make any changes to the appearance of this tool? 2. How did you find the colours that were used for this tool?  - Would you suggest the colours be changed? - And if so to what colours?  1. How did you find the size of the words used in this tool?  - Would you suggest we change the size of the words?   And if so to what size? |
| **Format & Navigation** |
| 1. Does the information presented in the tool follow a logical progression?    - If not, how would you reorganize or present this information? 2. Do you like the Table of Contents appearing on every page? 3. Do you prefer the layout with the horizontal or vertical page orientation? |
| **Content Questions** |
| **Introduction Section**   1. Does the information in this section make sense? Is it easy to understand?  - **If no,** - What doesn’t make sense/isn’t clear? - Is the language appropriate? - Is there anything in this section that you were unsure about or had trouble understanding? - **If yes,** - What aspects of the section make it easy to understand?  1. Is there anything missing from this section?    - If so, what?    - Why would this be helpful? 2. Is there anything that should be removed/changed from this section?    - If so, what?   Why would you remove/change this? |
| **KT Project Management Section**   1. Does the information in this section make sense? Is it easy to understand?  - **If no,** - What doesn’t make sense/isn’t clear? - Is the language appropriate? - Is there anything in this section that you were unsure about or had trouble understanding? - **If yes,** - What aspects of the section make it easy to understand?  1. Is there anything missing from this section?    - If so, what?    - Why would this be helpful? 2. Is there anything that should be removed/changed from this section?    - If so, what?   Why would you remove/change this? |
| **Activities Section**   1. Does the information in this section make sense? Is it easy to understand?  - **If no,** - What doesn’t make sense/isn’t clear? - Is the language appropriate? - Is there anything in this section that you were unsure about or had trouble understanding? - **If yes,** - What aspects of the section make it easy to understand?  1. Is there anything missing from this section?    - If so, what?    - Why would this be helpful? 2. Is there anything that should be removed/changed from this section?    - If so, what?   Why would you remove/change this? |
| **Case-Study Section**   1. Does the information in this section make sense? Is it easy to understand?  - **If no,** - What doesn’t make sense/isn’t clear? - Is the language appropriate? - Is there anything in this section that you were unsure about or had trouble understanding? - **If yes,** - What aspects of the section make it easy to understand?  1. Is there anything missing from this section?    - If so, what?    - Why would this be helpful? 2. Is there anything that should be removed/changed from this section?    - If so, what?   Why would you remove/change this? |
| **Resources Section**   1. Does the information in this section make sense? Is it easy to understand?  - **If no,** - What doesn’t make sense/isn’t clear? - Is the language appropriate? - Is there anything in this section that you were unsure about or had trouble understanding? - **If yes,** - What aspects of the section make it easy to understand?  1. Is there anything missing from this section?    - If so, what?    - Why would this be helpful? 2. Is there anything that should be removed/changed from this section?    - If so, what?   Why would you remove/change this? |
| **Key Terms Section**   1. Does the information in this section make sense? Is it easy to understand?  - **If no,** - What doesn’t make sense/isn’t clear? - Is the language appropriate? - Is there anything in this section that you were unsure about or had trouble understanding? - **If yes,** - What aspects of the section make it easy to understand?  1. Is there anything missing from this section?    - If so, what?    - Why would this be helpful? 2. Is there anything that should be removed/changed from this section?    - If so, what?    - Why would you remove/change this? |
| **Appendices Section**   1. Does the information in this section make sense? Is it easy to understand?  - **If no,** - What doesn’t make sense/isn’t clear? - Is the language appropriate? - Is there anything in this section that you were unsure about or had trouble understanding? - **If yes,** - What aspects of the section make it easy to understand?  1. Is there anything missing from this section?    - If so, what?    - Why would this be helpful? 2. Is there anything that should be removed/changed from this section?    - If so, what?    - Why would you remove/change this? |
| **Overall Questions** |
| 1. Is there anything missing from this tool?    1. If so, what?    2. Why would this be helpful? 2. Is there anything that should be removed/changed from this section? 3. If so, what?   Why would you remove/change this? |
| **System Usability Scale*** |
| - *See system usability table on next page. - I’m going to ask you 10 short questions based on the overall tool usability. These are standardized questions. - I’ll ask for your response on a scale from 1-5, with 1 being ‘Strongly disagree’ and 5 being ‘Strongly agree’.   Do you have any questions before I proceed? |
| **Revisiting Overall Tool Comments** |
| Do you have any other feedback, comments, or concerns regarding the tool that they would like to bring up? |
| **Demographic Questions** |
| - I have no more questions related to the tool. - If there is nothing further that any of you would like to add, I have a few **voluntary** demographic questions and then we will wrap up the interview.   - **You can elect to say “prefer not to answer” for any question.**   - We will also send you a demographic survey following our discussion. You can complete this survey in your own time. - The purpose of collecting this information is to report back to our funder on participant demographics.  1. Please indicate how many years you have been working in KT:   _______________________   1. Which province do you work in?   _______________________ |
| **Thank you again for taking time to speak today.** We will be in touch by email with any follow up comments. Please feel free to follow up with any insights we may have missed in our discussion today. We appreciate your insights! |

**System Usability Scale***

|  | Strongly disagree |  |  |  | Strongly agree |
| --- | --- | --- | --- | --- | --- |
| - - - 1. I think that I would like to use this tool frequently | 1 | 2 | 3 | 4 | 5 |
|  | | | | | |
| - - - 1. I found the tool unnecessarily complex | 1 | 2 | 3 | 4 | 5 |
|  | | | | | |
| - - - 1. I thought the tool was easy to use | 1 | 2 | 3 | 4 | 5 |
|  | | | | | |
| - - - 1. I think that I would need the support of a technical person to be able to use this tool | 1 | 2 | 3 | 4 | 5 |
|  | | | | | |
| - - - 1. I found the various functions in this tool were well integrated | 1 | 2 | 3 | 4 | 5 |
|  | | | | | |
| - - - 1. I thought there was too much inconsistency in this tool | 1 | 2 | 3 | 4 | 5 |
|  | | | | | |
| - - - 1. I would imagine that most people would learn to use this tool very quickly | 1 | 2 | 3 | 4 | 5 |
|  | | | | | |
| - - - 1. I found the tool very cumbersome to use | 1 | 2 | 3 | 4 | 5 |
|  | | | | | |
| - - - 1. I felt very confident using the tool | 1 | 2 | 3 | 4 | 5 |
|  | | | | | |
| 10. I needed to learn a lot of things before I could get going with this tool | 1 | 2 | 3 | 4 | 5 |
