# Supplementary material for: Development and usability testing of tools to facilitate incorporating intersectionality in knowledge translation
Source: BMC Health Serv Res. 2022 Jun 27;22:830. doi: 10.1186/s12913-022-08181-1 (PMC9238081; doi:10.1186/s12913-022-08181-1)
Supplement: Supplementary file 3 — Additional file 3: Appendix C. Development committee discussions and decisions for tool drafts. [file 12913_2022_8181_MOESM3_ESM.docx]

Appendix C– Development committee discussions and decisions for tool drafts

|  | Discussion theme | Decision made to tools |
| --- | --- | --- |
| Brainstorming | | |
| Purpose | - Necessity for an overarching guide to intersectionality - Three tools for each of the three prioritized stages of the Knowledge-to-Action Cycle - Not re-creating tools that already exist | - Create a minimum of four tools:   - Intersectionality Guide   - Identify problem/ evidence-to-practice gap: Reflection Workbook   - Assess barriers to knowledge use: Guide for Common Approaches to Assessing Barriers & Facilitators to Knowledge Use   - Select, tailor and implement interventions: Selecting, Tailoring and Implementing KT Interventions Workbook - Tools will reference other intersectionality tools that are freely available online |
| Format | - Comprehensive resources that can be used independently by implementation practitioners - Assume that implementation practitioners will not have access to an intersectionality specialist | - Longer guides/workbooks that contain empty boxes for users to fill in - Hyperlinks to external sites for digital copies - Full reference section should be included in tools |
| Draft 1 (pre-usability testing) | | |
| Purpose | - Assume that implementation practitioners have a basic awareness of implementation - Include project management language and steps - Build in reflection questions on power dynamics and relationships  \|  \| \| --- \| | - Include language and content in tools that assumes that a reader has a basic awareness of implementation - Include project management steps, including intersectional considerations, within the Intersectionality Guide - Include content relating to power dynamics and relationships within the Reflection Workbook |
| Format | - Encourage open-ended questions and that there are no right answers - Include a Table of Contents within each tool - Tools should have both online viewing (PDF) and printing capabilities | - Include considerations and questions written in an open-ended manner that encourages reflection - Include a table of contents for all tools - Tools should fit on standard printer paper and should be formatted vertically |
| Clarity | - Running example of a KT project - Include an introductory page to introduce reader to a tool | - Include the Mobilization of Vulnerable Elders (MOVEs) project to all relevant tools - Include an introductory box to the first page of each tool |
