# Supplementary material for: Development and usability testing of tools to facilitate incorporating intersectionality in knowledge translation
Source: BMC Health Serv Res. 2022 Jun 27;22:830. doi: 10.1186/s12913-022-08181-1 (PMC9238081; doi:10.1186/s12913-022-08181-1)
Supplement: Supplementary file 4 — Additional file 4: Appendix D. Usability testing issues for development committee discussion and decisions. [file 12913_2022_8181_MOESM4_ESM.docx]

Appendix D– Usability testing issues for development committee discussion and decisions

| Discussion theme | | Decision made to tools |
| --- | --- | --- |
| Purpose | - Indigenous considerations need to be included - Include additional resources and guidance within the Intersectionality Guide - Case-studies are too research focused and too simplistic - Tools are too focused on research and epidemiology paradigms - Some activities (e.g., Social Identity Statement activity) are constructed for highly privileged individuals. These activities may further marginalize individuals. - Some reflection questions and content feel repetitive - Need for transparency of why certain definitions were selected, who was on the committee - Need guidance on how to prioritize which intersecting categories are most important to an implementation project - Tools should consider/respect different ways of knowing | - Included a project limitation statement, including indigenous considerations, to the tools and project website - Additional resources and links added to the Intersectionality Guide - Revised the case study and tool content to be implementation practice oriented, including context nuances - Removed the Social Identity Statement activity from Intersectionality Guide - Merged reflection questions that had similar content - Added an introduction page(s) to all tools that includes project background and overview of who created the tools - Added a reflection point that prioritizing intersecting categories is complex and there is no right answer. Team discussion is encouraged. - Added prompts in multiple tools on defining evidence, high-quality evidence, and credible evidence |
| Format | - Tools are too long. In particular, the Selecting and Tailoring KT Interventions Workbook is “information overload.” - Format question responses boxes as tables or graphics, where possible. Use additional visualizations. - Create more white space on pages - Use different colours to delineate different sections | - Created one-page versions of two tools - Shortened content on a sub-phase from a tool and moved content to an appendix - Reformatted reflection questions onto intersectional flower image, added tables - Worked with graphic designer to create more visualizations, white space, and use different colours for different tool sections |
| Clarity | - Language used in the tools is highly research oriented - Consider separating sub-questions from overarching reflection questions - Definitions of privilege, disadvantage, oppression - Need to confirm who these tools are for | - Revised tool language to be more plain-language and practical - Keep existing grouping of reflection questions and sub-questions - Keep plain-language definitions of privilege, disadvantage, and oppression aligned across tools - Added an introduction question to initial tool pages outlining the intended audience |
