# Supplementary material for: Development and usability testing of tools to facilitate incorporating intersectionality in knowledge translation
Source: BMC Health Serv Res. 2022 Jun 27;22:830. doi: 10.1186/s12913-022-08181-1 (PMC9238081; doi:10.1186/s12913-022-08181-1)
Supplement: Supplementary file 5 — Additional file 5: Final intersectionality and knowledge translation tools. [file 12913_2022_8181_MOESM5_ESM.pdf]

Additional File 5

Intersectionality and Knowledge Translation Tools

Intersectionality & Knowledge Translation (KT)

Intersectionality Guide

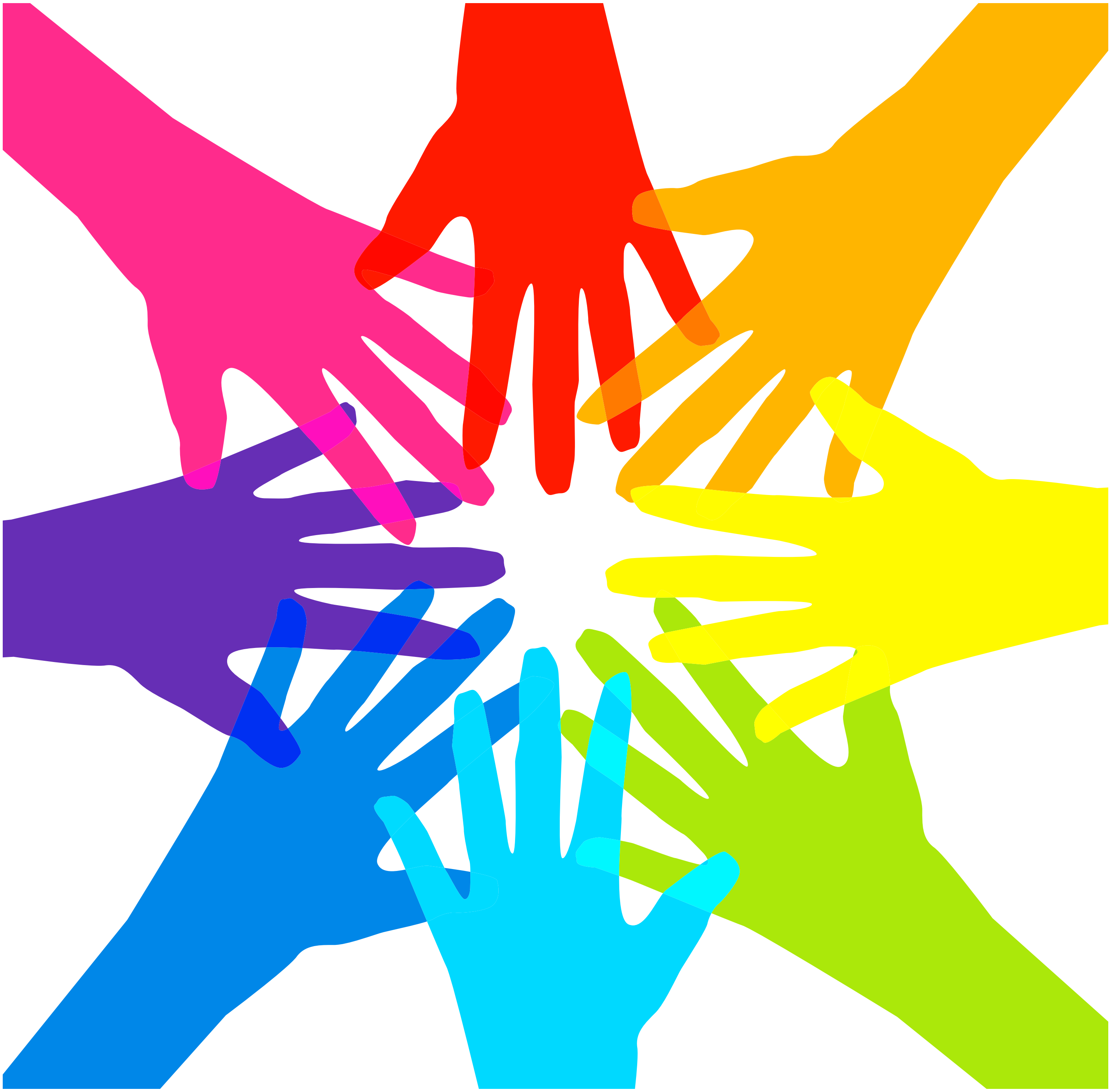

Table of Contents

Introduction 03
Summary of background on the guide

What is Intersectionality? 04
Detailed explanation of intersectionality and its history

KT Project Management 05
Considerations for designing and implementing a KT strategy with an intersectional lens

Step 1: Initiation 06
Define the KT project and set expectations for participating in the KT project

Step 2: Planning 08
Create schedules, task lists, resource lists, and a budget.

Step 3: Execution 09
Implement the KT project plan

Step 4: Monitoring 10
Evaluate whether the execution is aligned with the KT project plan

Step 5: Close 11
Reach the KT project goal

Activities for Applying Intersectionality 12
Reflections on intersectionality through individual and team-based activities

Resources 14

Key Terms 15

Appendices 18
Additional content for consideration

Introduction

Why should you use this tool?

The purpose of this tool is to inform KT intervention developers about intersectionality and how to take an intersectional approach in KT.

By applying an inclusive and equitable lens to KT interventions, you can design more effective interventions that address the complex realities of the people you work with.[1,2,3,4]

This tool relates to the overall **Knowledge-to-Action (KTA) Cycle**[5] and can be used in multiple situations (e.g., undertaking policy analysis, writing grant proposals, and conducting citizens' panels).

What is knowledge translation?

Knowledge translation (KT) is the process of moving evidence into health care practice.[6]

KT intervention developers are people who create KT interventions designed to improve health care.

For example, a KT intervention developer may design a KT intervention to change how often nurses encourage patients to exercise in long-term care homes. The KT intervention may include restructuring nurses' workflow and delivering in-person education sessions.

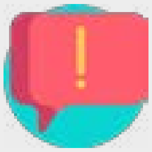

Please note: Taking an intersectional approach is needed to recognize the importance of individuals' social identities within the greater context of systems and structures of power. These reflect macro systems of privilege and oppression. Keep in mind that recognizing areas of advantage, disadvantage, and oppression may bring up feelings of confusion, guilt, distress, among others. It is okay to feel uncomfortable. There is a difference between feeling uncomfortable and unsafe.

How do you take an intersectional approach to KT?

As intervention developers, we take an intersectional approach to KT by considering the dynamic nature of social identities and their interactions with social structures and systems.

You consider these social identities and their interactions at all stages of the KT process. When doing this, you think about the identities of the people designing (e.g., KT intervention developers) and receiving (e.g., clinicians) the KT intervention and those affected by the intervention (e.g., patients).

What should you expect from this tool?

It provides background information on intersectionality, which will help you take an intersectional approach to your work. It also outlines how to consider intersectionality during each step of KT project management. In addition, it contains resources and activities you can apply to our work.

You can use this tool anytime during your project and can revisit it as often as you need to.

Who made this tool?

This tool was collaboratively developed in an iterative fashion by an interdisciplinary team of KT scholars, KT intervention developers, intersectionality scholars, and adult education experts.

Project limitations

See Appendix A for a project limitation statement.

This tool cannot be broadly applied to Indigenous peoples, and there may be more culturally appropriate models, theories, and frameworks that are useful to consider when conducting projects that involve Indigenous communities.

What is intersectionality?

What is intersectionality?

Intersectionality* is a way of looking at the world that recognizes that people's experiences are shaped by a combination of social factors, including their gender, racialization, age, among others.[7-13] These experiences occur within and interact with connected systems and structures of power, such as sexism and racism.[7-13]

Note that there are various definitions of intersectionality and that they are evolving.

History of intersectionality

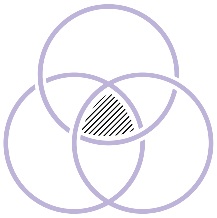

For an overview of intersectionality, refer to this video (available with subtitles) from **TED. Kimberlé Crenshaw's The Urgency of Intersectionality:** https://tinyurl.com/gs2dkny[14]

Kimberlé Crenshaw coined the term "intersectionality." Intersectionality is rooted in black feminist thought and the advocacy work of black feminists in the 1980s.[7-10]

To hear about Jamia Wilson's lived experience with intersectionality, visit **Race Forward #RaceAnd: Jamia Wilson:** https://tinyurl.com/yybfgksm[15]

What are intersecting categories?

Intersecting categories include age, gender identity, sex, and other aspects of one's lived experience. These aspects interact to form a person's identity (See Figure 1).[2,12,13] One's intersecting identities reflect larger systems of oppression/ privilege (e.g., sexism, ageism).[2,12,13]

A person's social identities shape their experiences in the world and how they view it (including the conscious and unconscious biases they hold).

You will learn more about how to consider your biases as you start to look at the KT project management steps.

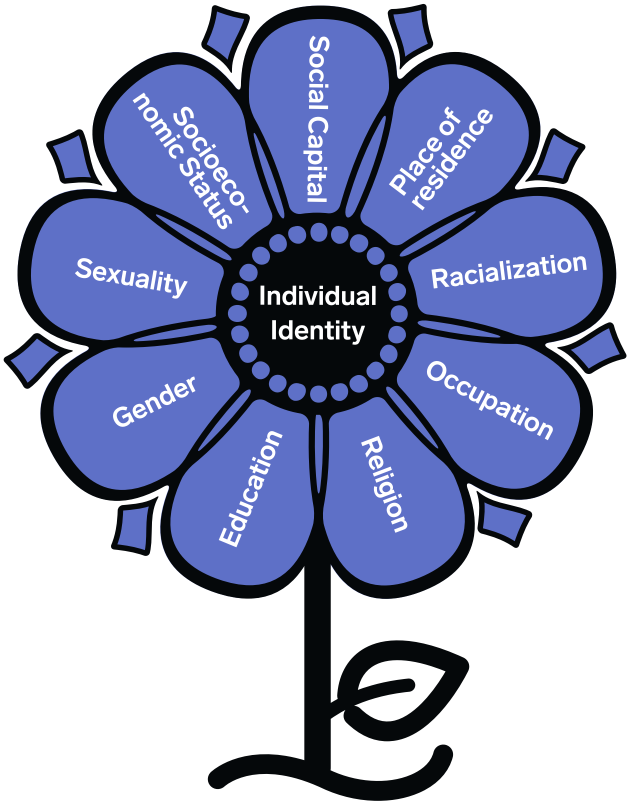

Figure 1. Visual representation of some intersecting categories.[12,16,17] The categories mentioned in this figure are not an exhaustive list.

KT Project Management Steps

A KT project involves designing and implementing a KT strategy

Consider your own biases

Before starting your KT project, consider the biases you may have as an individual and as a team. A bias is a preconceived judgment for or against a particular individual or group.[18]
A bias can be conscious or unconscious:

- Conscious bias (also known as explicit bias) is within one's conscious awareness.[18]
- Unconscious bias (also known as implicit bias) is beyond one's conscious awareness.[18]

To learn more about unconscious bias, view the **University of California, San Francisco's Office of Diversity & Research Unconscious Bias Resources:**
https://tinyurl.com/y5bjazb7[18]

Review the 5 key steps of KT project management to the right and reflect on how your biases will impact the KT project.

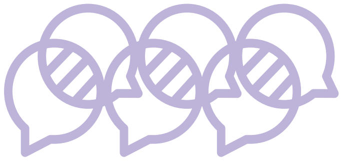

"Taking an intersectional approach doesn't mean that your activities include everyone, can overcome every obstacle, and accommodate every possible situation. It means you are aware of the gaps, ensuring that your work and practices are not creating obstacles and that you are working towards fair and equitable opportunities for everyone." [19]

- Multicultural Centre for Women's Health, 2017

01 Initiation

- Define the KT project.
- Set expectations for participating in the KT project.

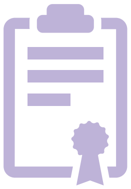

02 Planning

- Create schedules, task lists, resource lists, and a budget.

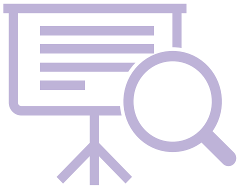

03 Execution

- Implement the KT project plan.

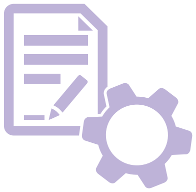

04 Monitoring

- Evaluate whether the execution is aligned with the KT project plan.

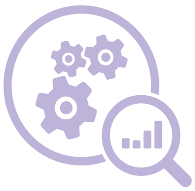

05 Close

- Reach the KT project goal.

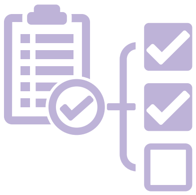

01 Initiation

Define the KT project and set expectations for participating in the project

Setting up our team

- ☐ Reflect on and recognize how your values, experiences, knowledge, and social identities may influence your work.[9] (See **Appendices** for examples of different activities to consider using.)

  This reflection may make you feel uncomfortable. That is OK.

  - Feeling uncomfortable (e.g., feeling uneasy when you are confronted with stereotypes about a group) is not the same as feeling unsafe (e.g., feeling that your well-being is threatened).
  - If a person feels unsafe, follow your organization's protocol and relevant legislation (e.g., Ontario's *Occupational Health and Safety Act*).
- ☐ Encourage team members to reflect, but do not require members to disclose their responses.
  - Members may identify with a number of marginalized identities; they may feel pressure to speak or fear being tokenized.[13]
- ☐ Reflect on power dynamics that may exist on the team. Remember that power is relational and includes experiences of power over others and with others (working together).[20]
  - For an in-depth discussion on power and its central role in intersectional analysis, see **Hankivsky, O., & Cormier, R. Intersectionality: Moving Women's Health Research and Policy Forward:** https://tinyurl.com/y47827xl[21]
- ☐ Prioritize, include, and respect the voices of those who experience and are impacted by the KT intervention.[13,22]
- ☐ Ask team members how they want to be involved in the project. Be flexible in meeting their needs, desires, and ways of participating.
- ☐ Celebrate each team member's different skills; do not assume each member will contribute identically.[19]
  - For example, encourage team members to share their ideas through different means including visual art and poetry.

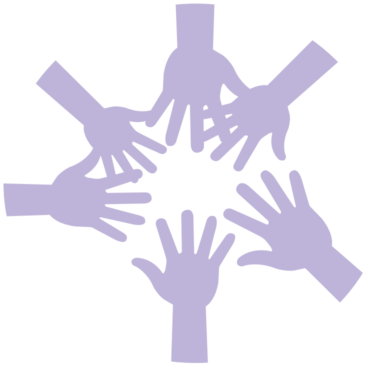

- ☐ Do not assume you know team members' social identities.
- ☐ Consider how your organization's policies, practices, or procedures could create advantages or disadvantages to participating in the project based on someone's intersecting categories.[2]
- ☐ Establish how decision-making will occur. Does the process let all voices have a say?[19]

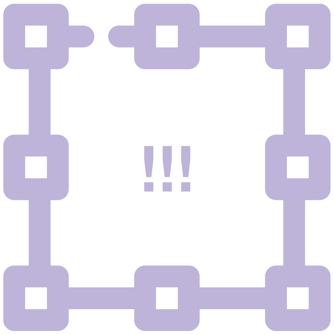

- ☐ Create a 'safe space' for project team members to engage.[23]
  - Consider **Lynn Weber's (1990) Guidelines for Discussion:** https://tinyurl.com/y34gx9mr[24]
  - Respect team members' requests for confidentiality.

01 Initiation

...continued

Accessibility

- ☐ Ask team members if they require any accommodations to participate.
    - In advance, inform funders that accessibility resources are important to the project. Include them in estimations
    - In communications, include comments such as "if you require accommodations of any sort in order to participate in these activities, please contact ____."
    - Do not assume what accommodations a person needs; provide an active offer for team members to come to you with accommodations requests.
        - Collaboratively craft communication norms (e.g., agree to spell out acronyms in each project document).
    - Collaboratively develop a project vision statement that reflects an intersectional approach.[13]

To help plan all communications and events with accessibility and inclusivity in mind, use these resources:

CRIAW Diversity Through Inclusive Practice: An Evolving Toolkit for Creating Inclusive Processes, Spaces & Events:
https://tinyurl.com/y3rmkgtg[25]

Ontario Council of University Libraries Accessibility Information Toolkit for Libraries:
https://tinyurl.com/y3yry25a[26]

Ryerson University Hosting Accessible Events or Meetings:
https://tinyurl.com/y27texys[27]

Work to establish and nurture trust.[19,28]

These suggestions apply across different project management steps:

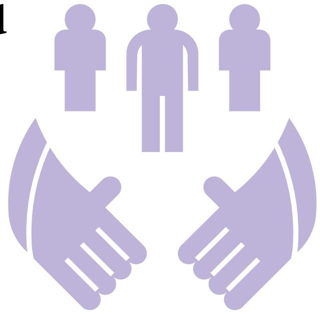

- ☐ Approach your interactions with others genuinely. Show curiosity and compassion.
- ☐ Do not be afraid of difficult self-reflections or discussions.
- ☐ Lead by example.
- ☐ Trust your team members and assume everyone is doing their best.
- ☐ Be consistent and predictable.
- ☐ Honour your commitments.
- ☐ Communicate honestly, openly, and often.
- ☐ Encourage questions.
- ☐ Explore shared experiences.
- ☐ Involve all individuals in a meaningful way (avoid tokenism). Do not expect individuals to speak on behalf of the groups they identify with.
- ☐ Be flexible.
- ☐ Think long term. Building trust takes time; expect to put in time to build trust.
- ☐ Consider how the problem is being framed. Each problem can be viewed from many perspectives. Be open to changing how you and the team frame the problem.[13,29]

02 Planning

Create schedules, task lists, resource lists, and a budget

Support our team members

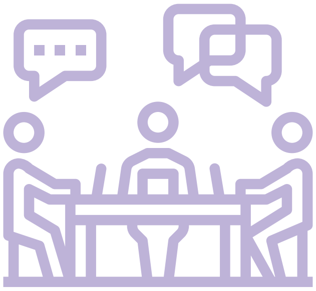

- ☐ Where possible, allow for flexible working conditions (e.g., hold online meetings, provide flexible hours for project work).
  - Plan to remunerate team members for their contributions (e.g., cash, gift cards).[19]
  - Provide access to supports for members' participation (e.g., interpreters, caregivers).[19]
  - Consider the living wage for the community we work in. A living wage is the "hourly wage a worker needs to earn to cover their basic expenses and participate in their community." [30]
    - For example, the living wage in Toronto, Ontario in November 2019 was $22.08. [30]

- ☐ Be particularly attentive to the needs of low-income participants. Consider offering bus tickets, parking, taxi fares, childcare, or other supports to make it easier for everyone to participate.

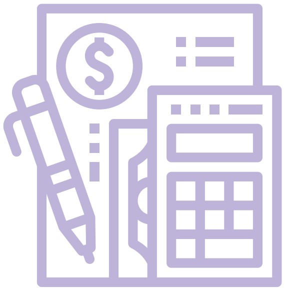

- ☐ Note that remuneration can be complex. Consider the following resources when deciding on compensation:

BC Centre for Disease Control. Peer payment standards:
https://tinyurl.com/yyuwdjku[31]

Canadian AIDS Society Peerology. A guide by and for people who use drugs on how to get involved:
https://tinyurl.com/y7levkhy[32]

Strategy for Patient-Oriented Research. Considerations when paying patient partners in research:
https://tinyurl.com/y6jvlta5[33]

CARFAC-RAAV. Minimum Recommended Fee Schedule:
https://tinyurl.com/yxoamchg[34]

- ☐ Add accessibility needs into budget planning and explore alternative opportunities for funding accessibility needs

- ☐ Be mindful of the resources available for the project when selecting compensation and participant supports. Remember that it is reasonable to run a project with limited scope.[29]

03 Execution

Implement the KT project plan

Consider intersectionality when implementing the plan

☐ Collect data in multiple formats that are accessible to project participants (e.g., participants can complete surveys on paper, online, over the phone).[23]

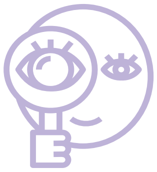

☐ Use sample sizes large enough to capture the multiple intersecting categories relevant to the project.[23]

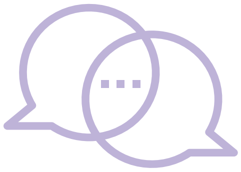

☐ Investigate different ways of disaggregating data to understand how intersecting categories impact/are impacted by the problem.[23]

☐ Determine which intersecting categories are most important to both the project and implementation context and why.[11]

☐ Collect data on multiple system levels (micro: individual; meso: regional or provincial; macro: national/international).[13, 23] Reflect on how these levels interact.

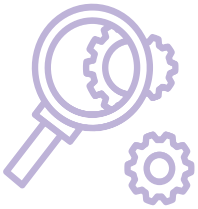

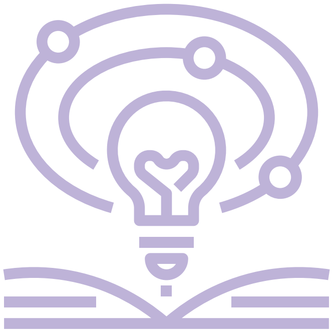

☐ Provide team members with resources on approaches to intersectionality in data analysis (e.g., Gender Based Analysis+[23])

☐ Support team members by letting them contribute in ways that work for them.[29]

- For example, if a team member prefers to provide feedback on materials verbally instead of via email, get their feedback by phone.

☐ Ensure that workload is fairly distributed based on team members' identified needs and preferences.

04 Monitoring

Evaluate whether the execution is aligned with the KT project plan

- ☐ Establish baseline indicators to measure involvement and effectiveness of the project team. Assess these indicators for different groups of people on the study team.[11,23] For example, ask patient partners if they feel they are participating in ways they want to. If not, why not?

  For more information on engagement, visit these resources:
  - **Arthritis Research Canada's Patient Engagement in Research (PEIR) Plan:** https://tinyurl.com/yyrugmc5[35]
  - **Deverka et al.'s Model for Effective Engagement:** https://tinyurl.com/y5ojc8nm[36]

- ☐ Obtain feedback from team members and project participants on whether current project management is meeting their needs.[11] Ask for suggestions for improvement.

- ☐ Acknowledge the gaps in the project's reach.[19] If not all project partners were able to participate, why not? For example, single parents may not have been able to participate because the project was not able to fund and arrange childcare.

- ☐ Consider intersectionality when monitoring and evaluating implementation:
  - Contextualize findings in relation to forms of oppression and power structures.[11]
    For more on power, visit: **Hankivsky, O., & Cormier, R. Intersectionality: Moving Women's Health Research and Policy Forward:** https://tinyurl.com/y47827xl[21]
  - Avoid large group categorizations that may miss intra group differences.[11] Balance the need to report disaggregated results with the need to ensure participant anonymity.

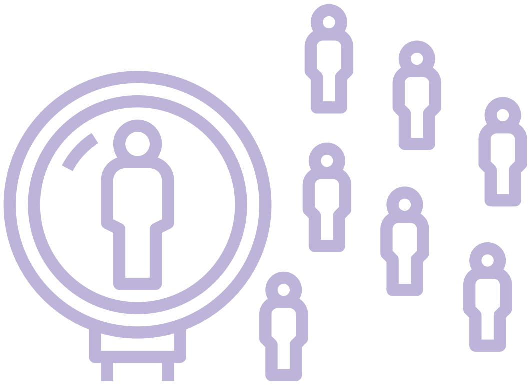

Be mindful of how similarities and differences are recognized in project results. Do not assume that findings are applicable or essential to everyone, and do not ignore historical and current patterns of inequality.[11] For example, do not assume that the results from the women in the project reflect all women. How might women's different intersecting categories (e.g., immigration status, education) impact the study results and your contextualization of the results?

05 Close

Reach the KT project goal

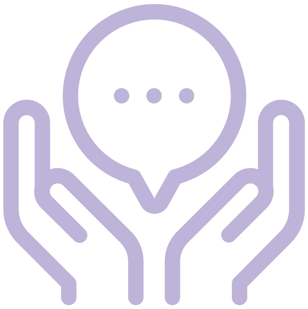

☐ Reflect on and discuss successes and lessons learned with the project team. Share these insights across other projects.

- Think about (1) what worked and why, (2) what did not work and why, (3) what could have been done differently, and (4) what adjustments and changes are required now.[37]
- Review project and organizational policies and incorporate insights.[11,23]

☐ Credit all project participants for their contributions.

☐ Disseminate results through multiple means.[11] Tailor dissemination formats to relevant audiences through such means as media, art, music, storytelling, and community feasts.[11]

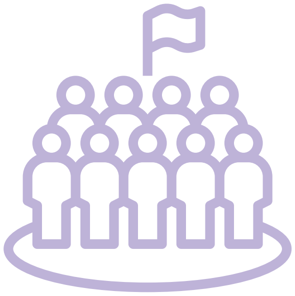

Think about shared goals and ideas for future projects with project partners. As you have already laid the groundwork for working together, reflect on how you can continue your positive working relationships with stakeholders.

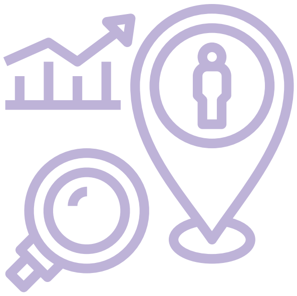

"Our social locations are not fixed. They depend on the specific situations and settings in which we find ourselves: our political and historical contexts, as well as the forces that govern our behaviour and operate around us such as laws, policies, institutions and media. Our social locations arise from a constellation of many co-operating factors and interactions of power and discrimination."[19]

- Multicultural Centre for Women's Health, 2017

Applying Intersectionality:

Activities to complete individually and/or with the project team

The next section provides an overview of the activities that you can complete individually or as a team. Before you look at the activities available, there are guidelines you need to consider to take an intersectional approach to your work.

When leading and participating in the activities highlighted on the next page, consult, adapt, and apply Weber's (1990) discussion guidelines.[24] Share the following guidelines with your team:

- ☐ Acknowledge that racism, classism, sexism, heterosexism, and other institutionalized forms of oppression exist.
- ☐ Acknowledge that we are all systematically taught misinformation about our own group and members of other groups. This is true for everyone, regardless of our group(s).
- ☐ Assume that both the people you study and the members of the team always do the best they can.
- ☐ Agree not to blame yourselves or others for the misinformation we have learned but to accept responsibility for not repeating misinformation once we have learned otherwise.
    - Recall that feeling uncomfortable (e.g., feeling uneasy when you are confronted with stereotypes about a group) is not the same as feeling unsafe (e.g., feeling that your well-being is threatened).
    - If you or a team member feels unsafe, follow your organization's protocol and relevant legislation (e.g., Ontario's Occupational Health and Safety Act).

For see more on how to understand unique and shared experiences of others, visit: **TV 2 Danmark All That We Share:** https://tinyurl.com/hlgg3qk [38]

- ☐ Actively explore stories about people in your own group and in others
- ☐ Share information about our groups with other team members and never demean, devalue, or "put down" people because of their experiences.
- ☐ Agree to actively combat myths and stereotypes about your own groups and other groups so we can break down the walls that prohibit group cooperation and group gain.
- ☐ If possible, refer team members who ask for additional support to your organization's Employee Assistance Program.
- ☐ Consider other logistics before starting the activities (e.g., rules of engagement, confidentiality, privacy, informing others about what to expect, reinforcing safe spaces)

How can a project facilitator support conducting the activity?

- ☐ Ideally, appoint an activity facilitator who has experience/expertise with discussions on intersectionality, privilege, and oppression.
- ☐ Contact your organization's Diversity, Equity, and Inclusion Office (or similar) for suggestions.

What response(s) may the activity elicit?

Recognizing areas of advantage or disadvantage may bring up feelings of confusion, guilt, distress, and pride among others.

Appendix	Time commitment	Purpose	Who completes the activity?
**Consider Your Own Diversity: Intersectionality Wheel** (See Appendix B)<br><br>KT Project Management Step 01: Initiation	15 minutes	To help individuals explore areas where they experience advantages and/or disadvantages in their lives.	Individuals. Do not force team members to disclose their responses.
**Critical Self-Reflexive Practice** (See Appendix C)<br><br>KT Project Management Step 01: Initiation	30 minutes	To help individuals pay attention to power through critical self-reflective practice. To help team members collaboratively become more aware of power and seize opportunities to challenge assumptions and renegotiate power.	Individuals, who engage in group discussions with the project team.
**Small Group Exercise: Considering Barriers to Health** (See Appendix D)<br><br>KT Project Management Step 01: Initiation	1.5 hours	To help team members recognize how various categories intersect and have compounded impacts.	Project team

Intersectionality Resources

These resources supplement other resources embedded in the Guide.

More information on intersectionality

An animated video teaching the basics of intersectionality through different stories.

Teaching Tolerance. Intersectionality 101:
https://tinyurl.com/y7kwvj6w[39]

Provides useful tools to use during workshops that focus on intersectionality.

Rainbow Health Network. Training for Change: Practical Tools for Intersectional Workshops:
https://tinyurl.com/yyvrgvhm[40]

A primer providing intersectional perspectives on health research and policy related to the context of women's health.

Hankivsky, O., & Cormier, R. Moving Women's Health Research and Policy Forward:
https://tinyurl.com/y47827xl[21]

Tools / Courses / Guides

A plain language guide, focusing on how understanding social problems can be altered through applying intersectionality.

Olena Hankivsky. Intersectionality 101:
https://tinyurl.com/y485633k[13]

Provides an introductory course on the process of Gender Based Analysis Plus (GBA+), where participants "assess how diverse groups of women, men and non-binary people may experience policies, programs, and initiatives."

Department for Women and Gender Equality Gender Based Analysis Plus Course:
https://tinyurl.com/y4n4zaca[23]

A guide for minimizing harm to the individuals impacted by the policies and practices developed.

Government of Canada: Trauma and violence-informed approaches to policy and practice:
https://tinyurl.com/y64n39z6[41]

Important considerations

A newsletter focusing on the topic of intersectionality while highlighting "relevant information (e.g., resources), research, and promising practice."

The Learning Network. Intersectionality Newsletter:
https://tinyurl.com/yxwhdcat[11]

A practical guide for both individuals and organizations who are seeking to learn more about intersectionality.

The International Lesbian, Gay, Bisexual, Transgender, Queer and Intersex Youth & Student Organisation. Intersectionality Toolkit :
https://tinyurl.com/y2sga6c7[42]

Tips for using language that place people first rather than adding more stigma with stereotypes and assumptions.

Youth Mental Health Canada. People First Language:
https://tinyurl.com/y4saads8[43]

Key Terms

The definitions below provide the vocabulary needed to take an intersectional approach with your work. There are a range of definitions possible for these terms, and these definitions will change over time. Be sure to reflect with your team and community partners on the definition(s) your team will use.

Accessibility: Accessibility involves removing the barriers faced by individuals with a variety of disabilities (which can include but are not limited to physical, sensory, cognitive, learning, and mental health disabilities) and the barriers (e.g., people's attitudes, system-level barriers) that impede an individual's ability to fully participate in social, cultural, political, and economic life.[2]

Class: A person or group's real or perceived economic status or background.[44]

Disability: The UN Convention definition of people with disabilities states that "Persons with disabilities include those who have long-term physical, mental, intellectual, or sensory impairments which in interaction with various barriers may hinder their full and effective participation in society on an equal basis with others."[45]

Equity: Each individual is given different supports so they have equal access to opportunities.[46]

Equality: Equal conditions for realizing opportunities. For more on equal treatment, equity, and equality, please see **Department for Women and Gender Equality (2018) GBA+: Equality or Equity?** https://tinyurl.com/y3onf5gr[23]

Ethnicity: Ethnicity is a socially constructed concept that encompasses an individual's cultural associations. This typically includes shared ancestry and/or geographic origins with common languages, cultural traditions, and cultural symbols, including but not limited to values and norms, religion, and diet.[17,47]

Gender: The Canadian Institute of Health Research (CIHR) defines gender as "the socially constructed roles, behaviours, expressions and identities of girls, women, boys, men, and gender diverse people. It influences how people perceive themselves and each other, how they act and interact, and the distribution of power and resources in society. Gender is usually conceptualized as a binary (girl/woman and boy/man) yet there is considerable diversity in how individuals and groups understand, experience, and express it." [48]

For more on gender, please visit The 519 Glossary of Terms: https://tinyurl.com/y4l5kzol[49]

Impairment: A change in a person's physical or psychological function. The World Health Organization (WHO) emphasizes how the impairments, activity limitations, and participation restrictions that can encompass disability are a product of the interaction between an individual's body and the social and environmental barriers that exist within the society where an individual is positioned.[45,50]

Intersectionality: Intersectionality is a way of looking at the world that recognizes that human beings' experiences are shaped by a combination of social factors, including their gender, racialization, age, among others.[7-13] These experiences occur within and interact with connected systems and structures of power, such as sexism and racism.[7-13]

Intersectional approach in knowledge translation (KT): An intersectional approach to doing KT considers the dynamic nature of social identities and their interactions with social structures and systems. We consider these social identities and their interactions at all stages of the KT process. When doing this, we think about the identities of the people designing (e.g., KT intervention developers) and receiving (e.g., clinicians) the KT intervention and those affected by the intervention (e.g., patients). For example, we might explore how a young nurse who works as a KT intervention developer, identifies as non-binary and as a member of an ethnic minority group, and speaks English as an additional language may have unique barriers to working with mainly older white male doctors in leadership positions. Accordingly, she may implement an intervention differently than older white male doctors in leadership positions would.

Oppression: When a social group is systematically denied access or resources based on their membership in a social group that is generally a targeted or oppressed group.[51]

Key Terms

...key terms

Privilege: The unearned advantages that individuals benefit from by identifying or being born into certain groups.[52,53] Members of privileged groups typically do not recognize their advantages and instead view them as the way things simply are, leading to a normalization of privilege along with silence and denial about its influence on oppression.[53,54]

Racialization: Race has historically been viewed as a biological construct, representative of genetic similarities between groups from similar ancestries and geographic origins. However, the existence of distinguishable genetic differences between groups has been widely refuted, leading to a shift from race being viewed as a biological construct to race being viewed as a social-political construct where groups from similar ancestral and geographic backgrounds are grouped based on phenotypic genetic expression.[17,47]

Sex: The Canadian Institute of Health Research (CIHR) defines sex as "a set of biological attributes in humans and animals. It is primarily associated with physical and physiological features including chromosomes, gene expression, hormone levels and function, and reproductive/sexual anatomy. Sex is usually categorized as female or male but there is variation in the biological attributes that comprise sex and how those attributes are expressed."[48]

Sexual Orientation: The 519 defines sexual orientation as "The direction of one's sexual interest or attraction. It is a personal characteristic that forms part of who you are. It covers the range of human sexuality from lesbian and gay, to bisexual and straight."[49]

Stakeholders: Residents or groups who use or are affected by policies and procedures.[2] Other terms used to describe stakeholders can include but are not limited to partners, collaborators, allies, and contributors.

Stereotypes: Assumptions that all people in a particular group are the same without considering individual differences. Stereotypes are often based on misconceptions or incomplete information.[2]

Stigma: The Canadian Mental Health Association views stigma as "the negative stereotype and discrimination is the behaviour that results from this negative stereotype. For example, a woman with a mental illness may experience discrimination due to both sexism and her illness, and a racialized individual may experience discrimination due to both racism and their mental illness. In addition, living with discrimination can have a negative impact on mental health."[55]

Tokenism: The practice of making perfunctory or symbolic efforts to engage communities, patients, citizens, or members of other groups in an activity, especially by recruiting a small number of people from underrepresented groups to give the appearance of equality.[56]

Trauma: The Centre for Addiction and Mental Health defines trauma as "the lasting emotional response that often results from living through a distressing event. Experiencing a traumatic event can harm a person's sense of safety, sense of self, and ability to regulate emotions and navigate relationships. Long after the traumatic event occurs, people with trauma can often feel shame, helplessness, powerlessness and intense fear."[57]

Key Terms

The words we use matter.

Part of taking an intersectional approach is being aware of the language we are using.

The previous list of definitions is not an exhaustive list of key terms, and key terms and definitions may change over time. Review other living glossaries, such as the following:

Ontario Human Rights Commission Appendix 1: Glossary of human rights terms: https://tinyurl.com/y4bsaasm[58]

Learning Network Terminology: https://tinyurl.com/yydtqv3u[44]

The 519 Glossary of Terms: https://tinyurl.com/y4l5kzol[49]

For further reading

- Collins PH. Black feminist thought: Knowledge, consciousness, and the politics of empowerment. 2d ed. New York: Routledge, 2000.
- Crenshaw K. Mapping the margins: Intersectionality, identity politics, and violence against women of color. Stanford Law Review. 1991; 43(6): 1241-1299. doi: 10.2307/1229039
- Hankivsky O, Grace D., Hunting G., et al. An intersectionality-based policy analysis framework: Critical reflections on a methodology for advancing equity. International Journal for Equity in Health. 2014;13(119). doi: 10.1186/s12939-014-0119-x
- Hankivsky O. Women's health, men's health and gender and health: Implications of intersectionality. Social Science and Medicine. 2012; 74(11): 1712-1720.
- Newman DM. Identities & inequalities: Exploring the intersections of race, class, gender, and sexuality. 2d ed. New York: McGraw-Hill, 2012.
- Rice C, Harrison E, Friedman M. Doing justice to intersectionality in research. Cultural Studies ←→ Critical Methodologies. 2019; 00(0): 1-12.

Appendix A: Project Limitations

We acknowledge that the work of our Canadian Institutes of Health (CIHR)-funded team grant was conducted on unceded lands that were the traditional territories of many people, including the Algonquin, Cree, Dakota, Dene, Huron-Wendat, Mississaugas of the Credit River, and the Musqueam Peoples, and on the homeland of the Métis Nation. We acknowledge the harms of the past and the harms that are ongoing. We are grateful for the generous opportunities to conduct work on these lands.

In 2017, the CIHR launched an opportunity for team grants in gender and KT. This opportunity (sponsored by the Institute of Gender and Health) was developed to recognize that the field of KT had yet to thoughtfully integrate gender into its research agenda. The objectives of the CIHR team grant competition were to generate evidence about whether applying sex- and gender-based analysis to KT interventions involving human participants improves effectiveness, thereby contributing to improved health outcomes; contribute to a broader knowledge base on how to effectively and appropriately integrate gender into KT interventions; and facilitate the consideration and development of gender-transformative approaches in KT interventions.

In response to this call, we submitted a grant aimed at helping KT intervention developers use an intersectional approach when designing and implementing interventions to address the needs of older adults. We received feedback from the CIHR peer review committee that substantial concern was raised about our focus on intersectionality. In particular, the Scientific Officer's notes described that the focus on intersectionality would dilute the focus on gender and needed to be reconsidered. A meeting was subsequently held with the successfully funded team and this issue was raised again. We acknowledge the limitation that our intersectional approach comes at the expense of a minimized focus on gender. However, because intersecting categories, such as gender and age, are experienced together, we ultimately elected to use an intersectional approach as it encapsulates the lived experience of those we aim to impact.

A more significant limitation of our work is that we did not include First Nations, Inuit, and Métis community members in the grant proposal. As such, their needs and perspectives were not included in the research grant and, consequently, funded activities. Our team did not have established relationships or expertise in this area and as such, we felt it was inappropriate for our team to work on a grant in this area.

We strongly believe that consideration of gender and KT for Indigenous peoples should be a primary focus of a distinct team grant.

There are established best practices for community engagement with First Nations, Inuit, and Métis Peoples that begin with principles of collaboration, which take time to develop and must not be tokenistic. The principles for collaboration should ensure authentic engagement, shared respect, trust, and commitment to ensure long-term, mutually empowered relationships. These principles should also ensure that the research-related priorities meet the needs, perspectives, and expectations of the First Nations, Inuit, and Métis Peoples. Indigenous peoples have a long history of conducting research, and this tradition continues today with many Indigenous healers and scholars leading research in various areas. Indeed, there are many Indigenous scholars working in the KT field.

Because the team's work did not include First Nations, Inuit, and Métis Peoples and involve adhering to the principles that guide their engagement in research, the needs and considerations of these Peoples were not included in the work conducted in this team grant. As such, anyone who is considering using the outputs of this team grant needs to know that **they cannot be broadly applied to these Peoples and there may be other more culturally appropriate models/theories/frameworks that are useful to consider.** Similarly, because this research focused on older adults (and in particular, chronic disease management in older adults) **it does not apply to children and youth**.

We believe that any KT intervention work needs to begin with engaging the appropriate community and is only applicable when those communities are engaged throughout the research enterprise. Moreover, intersectionality involves deep immersion in the lived experiences and priorities of those communities. As a result, KT work requires immersive work with various populations and not just key informants to ensure the work meets the needs of the relevant populations.

We thank and acknowledge Dr. Lisa Richardson, Co-Lead, Indigenous Health Education, Faculty of Medicine, University of Toronto, for her time and expertise in reviewing this statement.

Appendix B: Activity - Consider Your Own Diversity[59]

Please note that the original reference activity has been modified for the purposes of this guide

What is the purpose of the activity?

To help individuals explore areas where they experience advantages and/or disadvantages in their lives. Do not force team members to disclose their responses.

Consider your own diversity:

Use the wheel diagram (below) to explore areas where you have experienced advantage or disadvantage in your life.

Circle the factors that bring you **ADVANTAGE**

Underline the factors that bring you **DISADVANTAGE**

In some cases it may be both!

The inner circle contains social factors that influence the extent to which we experience advantages or disadvantages in our lives. It is the intersection of these factors that influences the way we experience life in our municipality. These factors include sexual orientation, Indigenous ancestry, age, social class, education, sex, race and ethnicity, length of time in the community, gender identity, religion and spirituality, place of origin, marital or family status, geographical location, disabilities, language, income, immigration status, and other factors.

The middle circle contains positions or statuses we may fill within the organizations that carry varying amounts of power and influence. It is often the intersection of these positions, statuses, and social factors that determine our opportunities. These might include being a council or board member; being a manager/supervisor/staff member/student; type of occupation/ profession; length of service; union affiliation; department/ unit; and whether you are full-time, part-time, contract, casual, or volunteer employee.

The outer circle contains the ways in which people are discriminated against. Most of us experience more than one form of discrimination. These factors interact with wider social forces, such as history and the legacies of colonialism, patriarchy, economic exploitation, level of education, inaccessible legal systems, and racist immigration policies. Some forms of discrimination include ableism, racism, heterosexism, sexism, classism, ethnocentrism, transphobia, ageism, and homophobia.

Please see Appendix A for our project limitations.

Intersectionality Wheel Diagram

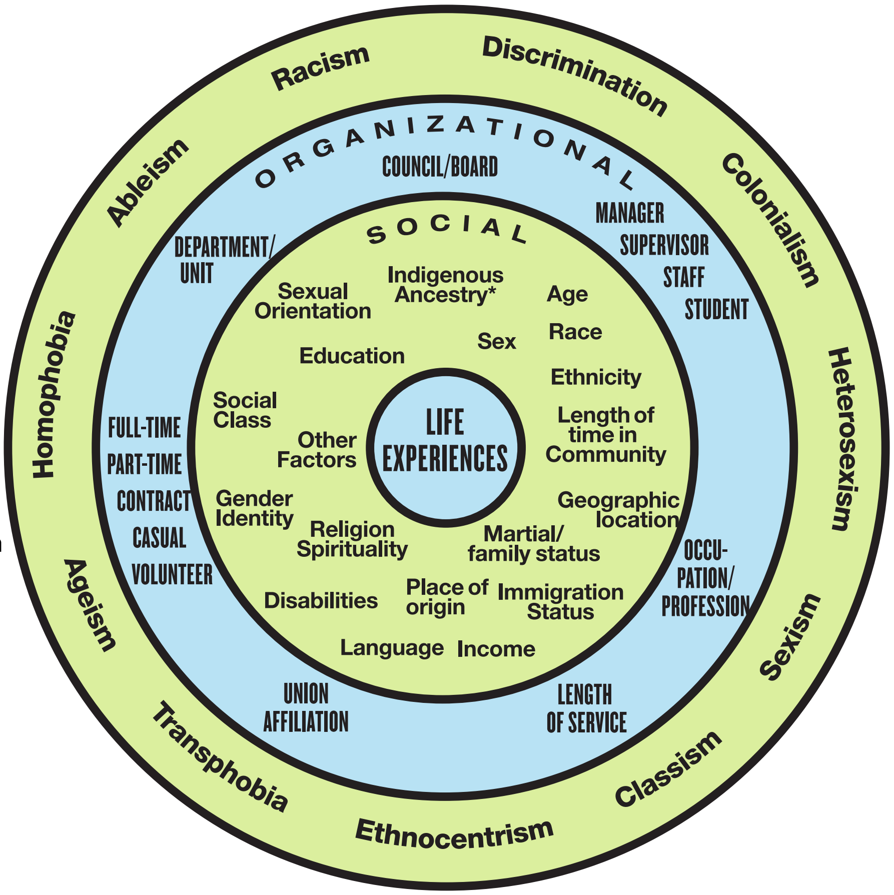

Appendix C: Activity - Self-Reflexive Practice Questions[60]

Please note that the original reference activity has been modified for the purposes of this guide

What is the purpose of the activity?

- To help individuals pay attention to power through critical self-reflective practice.
- To help team members work collaboratively to become more aware of power and take advantage of opportunities to challenge assumptions and renegotiate power.

Questions for the KT project team

1. **What are my personal values, experiences, interests, beliefs, and political commitments in the area of health we will be researching?**
2. **How do these personal experiences relate to social and structural locations** (e.g., gender identity, race, ethnicity, indigeneity, socioeconomic status, sexuality, gender expression, age, sexual orientation, immigrant status, religion) **and processes of oppression** (e.g., patriarchy, colonialism, capitalism, racism, heterosexism, ableism) **in the area of health we will be researching?**
3. **What are my personal values, assumptions, perspectives, and experiences related to people living with the health condition(s) or issue(s) we will be researching?**
4. **From your perspective, what current health inequities** (i.e., avoidable and unjust inequalities in health between and within groups of people) **exist related to the area of health we will be researching?**
5. **How do you think people with lived experience in this area of health would prefer to be involved in research and why?** What types of challenges would need to be addressed to make it easier for people living with this health condition or issue and their families and communities to become involved in research?
6. **Working together, how can we become more aware of and take advantage of opportunities where we can challenge each other's ideas and renegotiate power within our project team?** What does building resilience look like, feel like, and sound like to you?
7. **How do you think the issue of trauma may impact the area of health we will be researching?** (Remember to think about it both on the level of violence within relationships and on the larger level of colonialism, racism, sexism, homophobia, capitalism, ableism, etc.)
8. **What do you think are some of the ways in which we can make sure everyone feels safe when working together on this research project?** What does physical safety mean to you? Look like to you? Feel like to you? What does emotional/psychological safety mean to you? Look like to you? Feel like to you? What are some of the best ways we can work together to address trauma? (This will be discussed in the practice section too.)

Appendix D: Group Activity[40]

Please note that the original reference activity has been modified for the purposes of this guide

Small Group Exercise
Barriers to Health

What is the purpose of the activity?

To help individuals pay attention to power through critical self-reflective practice. To help team members work collaboratively to become more aware of power and take advantage of opportunities to challenge assumptions and renegotiate power.

Time to complete: 10 to 15 minutes,
plus 20 minutes to debrief

Preparation:

- If desired, review the terms and glossaries outlined in the "Key Terms" section of this guide to be sure you are familiar with the terms used in the activity.
- Retrieve copies of the **"Patient Profiles"** (see Handout 1 below). Distribute one copy to each small group.
- Provide one clinic intake form per group. (If your organization does not use intake forms, consider another type of commonly used form or tool [e.g., survey or interview guide].)
- Create copies of the **"Reflection Directions"** handout (see Handout 2 below) and distribute one to each person.

Set up:
Arrange participants into small groups of up to five members each, depending on the size of the group. Hand out copies of the Reflection Directions exercise. Give participants a moment to look at the questions.

Preface the exercise by saying "We are looking at the ways in which our organization may inadvertently create barriers for older adults in our community. But keep in mind that members of the older adult community come from every other community. So as you engage in this exercise, feel free to point out barriers to other groups because the first thing we know about an older adult might not be their (dis) abilities."

Give each group a clinic intake form (or other organizational form or tool). Direct everyone's attention to the instructions on the Barriers to Health handout. Read the instructions aloud. Tell the group they have 5 minutes to look at their intake form (or other organizational form or tool) and answer the questions on the sheet.

Hand out the Patient Profiles and explain that this is a brief outline about a service user or stakeholder who has just come in to meet you for the first time. Have them examine the intake form (or other organizational form or tool) again with this person in mind and consider if they notice any new issues.

Give the group an additional 5 minutes to wrap up.

Appendix D: Activity - Group Activity[40]

Please note that the original reference activity has been modified for the purposes of this guide

Small Group Exercise
Barriers to Health

Handout 1

Patient Profiles

Trevor: Hearing impaired, Black, 70, in a relationship with a woman, also has sex with men, comes to community health centre with a sore throat.

Anita: Trans woman, 65, wheelchair user, hearing loss, long-term female partner, attends women's group for survivors of domestic violence, comes to community health centre with a broken arm.

Raymond: Straight, white, trans man, mid-60s, long-term committed relationship with a woman, doesn't associate with LBGTI community, not visibly transgender, comes to community health centre for incontinence and referral for a mobility aid.

JC: Filipina, 40s, lesbian, single mother of two, caring for aging parent with dementia, comes to parenting group for families with children under 6.

Appendix D: Activity - Group Activity[40]

Please note that the original reference activity has been modified for the purposes of this guide

Small Group Exercise
Barriers to Health

Handout 2

Reflection Directions

Imagine you work for a broad-based health agency that provides a number of health and wellness services to a diverse community. The organization collects crucial information at each point of access to allow them to provide more holistic services. Critically examine the intake form samples (or a form or tool that your organization uses), and then answer these questions as a small group.

Take note of how language is used and also what language is not used.

Can you identify any barriers to health care access that might be present?

What social identity group(s) might be marginalized from a service that uses this form?

Look at your patient profile. Can you identify any reasons/issues that may prevent this client from comfortably accessing the service?

Look at your form again. Do you notice any new/different areas that may be barriers for this individual?

What form could that marginalization take?

What is the impact of these barriers on the following:
- a) The client's physical health?
- b) The client's mental health/well-being?
- c) The client's relationship with your organization?
- d) The client's relationship with the service provider?

Appendix D: Activity - Group Activity[40]

Please note that the original reference activity has been modified for the purposes of this guide

Please note that the common responses to this exercise below will vary based on the specific intake forms (or organizational forms or tools) being used.

Ask the group: "What things stood out immediately or seemed glaring?"

Common Responses for Patient Profiles:

- Intake form is written in English and on paper – marginalizes the blind community; the deaf community (whose first language might be American Sign Language [ASL]); and people who are not functionally literate, including non-English speakers and possibly English as a Second Language (ESL) speakers/newcomers.
- "Male" and "female" checkboxes only.
- Health cards necessary.
- Checkboxes for race/ethnicity, which are not usually representative/inclusive.
- Sexual orientation information not elicited.
- Sexual behaviour information not elicited.
- Privacy not available in waiting room/area while people are filling out very sensitive information.

Ask what other barriers became evident after looking at the patient profiles.

Trevor:

- An assumption that older adults are not sexually active may lead health care providers to ask Trevor different questions than they would ask a younger person.
- Because client identifies as straight, health care providers may not inquire about same-sex activities/relationships.
- Client is Black and may be assumed to be straight and cis-gender as a result of the tendency to equate queer identities/behaviours with whiteness/White culture.
- If intake forms do not elicit sexual behaviour information, person might feel awkward about bringing it up face-to-face or may feel judged if they do.
- Trevor may be misdiagnosed if the service provider (a) doesn't know that he engages in same-sex sexual activities or assumes that his sexual behaviours are heterosexual encounters and (b) isn't aware of health risks for men who have sex with men.
- This may lead to everything from a worsening of his condition to being prescribed and taking the wrong medication, which might have other effects on his physical health.
- Trevor would most likely search for a new health care provider.

Anita:

- Participants may comment on the level of physical accessibility of their organization; many buildings that can be accessed by wheelchair users do not have accessible washrooms or have other barriers inside the locations. These barriers may include internal doors without accessible buttons, bathroom fixtures that are out of reach, etc.
- Anita may be assumed to be straight because there are notions that (a) abuse does not happen in same-sex relationships and (b) people with disabilities are not sexual, and if they are, they somehow cannot be queer.
- Anita may be assumed to be cis-gender.
- Language use/climate in the group may prevent her from identifying as trans in this setting and may make her feel left out; no accommodations in group for those with hearing difficulties.
- Unable/unwilling to come out in the environment, which will impact the benefit that the program will have for her.
- She may stop attending if she feels she cannot be open about her relationships in a space designed for healing from bad relationships.

Appendix D: Activity - Group Activity[40]

Please note that the original reference activity has been modified for the purposes of this guide

Raymond:

- Because Raymond is not visibly transgender, he will be assumed to be cis-gender, especially if forms have options for sexual orientation but only M/F for gender/sex (as many do). That is, because he's straight, people may assume that he cannot be trans if they confuse gender and sexual identity/behaviour.
- Cis-gender identity may be reinforced once he reveals that he has a female partner, again because people do not often understand that gender identity and sexual orientation are different.
- Service providers may assume that biology is behind his incontinence.
- Service providers may incorrectly prioritize discussions around incontinence and forget to write the referral for the mobility aid. This requires Raymond to keep reminding the service provider that he has multiple needs.
- Some organizations may refuse to provide the service due to lack of training, information, or fear. Alternatively, they may turn this procedure into a training exercise for all of their staff, impacting on level of service, right to privacy, and dignity of the client.
- If any of these events occurred, Raymond could rightfully choose to file a complaint under the organization's anti-discrimination policy or even a human rights complaint if a satisfactory resolution is not reached.

JC:

- JC may be assumed to be straight because she has children and/or because she is from a non-White background.
- Because sexual orientation is assumed to be fixed, people may assume that she is not the biological mother of the kids because she has never had sex with a man or that she had children through a sperm donor.
- People may assume that she has a poor relationship with her parents because of her sexual orientation. Therefore, they may assume that she is not responsible for the care of her parents.
- Other people may assume she was previously married and came out after having children with her husband.
- If people realize that she is a lesbian, service providers or other parents may express open disapproval of her attending the location with her children.
- Some parents may go so far as to want their children isolated from her/hers.
- If the children pick up on the adults' sentiments, JC's kids could get bullied or teased at school or daycare.
- JC may not see herself and her family reflected in the materials if only items/curricula depicting hetero-normative families are displayed. The materials also do not speak about multigenerational caregiving duties (i.e., JC is responsible for both her children and her parent).

Guide References

1. Tannenbaum, C., Greaves, L. & Graham, I.D. Why sex and gender matter in implementation research. BMC Med Res Methodol 2016;16:145. doi:10.1186/s12874-016-0247-7

2. City of Ottawa and City for All Women Initiative. Advancing Equity and Inclusion – A Guide for Municipalities. CAWI. http://www.cawi-ivtf.org/sites/default/files/publications/advancing-equity-inclusion-web.pdf. Published June 2015.Accessed February 6, 2019.

3. Public Health Agency of Canada. Key Health Inequalities in Canada: A National Portrait – Executive Summary. https://www.canada.ca/en/public-health/services/publications/science-research-data/key-health-inequalities-canada-national-portrait-executive-summary.html. Published August 2018. Accessed February 6, 2019.

4. The Association for Women's Rights in Development. Intersectionality: A Tool for Gender and Economic Justice. Women's Rights and Economic Change. https://lgbtq.unc.edu/sites/lgbtq.unc.edu/files/documents/intersectionality_en.pdf. Published August 2004. Accessed February 4, 2019.

5. Graham ID, Logan J, Harrison MB. Straus SE, Tetroe J, Caswell W, Robinson N. Lost in Knowledge Translation: Time For A Map? J Contin Educ Health Prof. 2006; 26(1): 13.

6. Government of Canada. Knowledge Translation in Health Care: Moving from Evidence to Practice - CIHR. 2010. http://www.cihr-irsc.gc.ca/e/40618.html. Accessed May 28, 2019

7. Collins, P. H. Black feminist thought: knowledge, consciousness, and the politics of empowerment. Routledge. 1990. https://trove.nla.gov.au/version/21207078. Accessed February 4, 2019.

8. Crenshaw, K. Demarginalizing the intersection of race and sex: A black feminist critique of antidiscrimination doctrine, feminist theory and antiracist politics. U. Chi. Legal F. 1989;139.

9. Crenshaw, K. A Black feminist critique of antidiscrimination law and politics. The politics of law: A progressive critique. 1990;195.

10. Crenshaw, K. Mapping the Margins: Intersectionality, Identity Politics, and Violence against Women of Color. Stanford Law Review. 1991;43(6), 1241-1299. doi:10.2307/1229039

11. The Learning Network. Issue 15: Intersectionality. 2015. http://www.vawlearningnetwork.ca/sites/vawlearningnetwork.ca/files/Intersectioanlity_Newsletter_FINAL2.pdf. Accessed February 7, 2019.

12. Hankivsky, O., Grace, D., Hunting, G. et al. An intersectionality-based policy analysis framework: critical reflections on a methodology for advancing equity. Int J Equity Health. 2014; 13(119) doi:10.1186/s12939-014-0119-x

13. Hankivsky, O. Intersectionality 101. The Institute for Intersectionality Research & Policy, SFU. http://vawforum-cwr.ca/sites/default/files/attachments/intersectionallity_101.pdf. Published April 2014. Accessed February 7, 2019.

14. Kimberlé Crenshaw: The urgency of intersectionality [Video]. TED Women 2016. https://www.ted.com/talks/kimberle_crenshaw_the_urgency_of_intersectionality. Published October 2016. Accessed February 7, 2019.

15. #RaceAnd: Jamia Wilson [Video]. Youtube. https://www.youtube.com/watch?v=4o-qnznd10Y. Published May 11, 2016. Accessed February 7, 2019.

16. PROGRESS-Plus. Cochrane Equity Methods. https://methods.cochrane.org/equity/projects/evidence-equity/progress-plus. Accessed November 12, 2019.

17. O'Neill, J., Tabish, H., et al. Applying an equity lens to interventions: Using PROGRESS ensures consideration of socially stratifying factors to illuminate inequities in health. Journal of Clinical Epidemiology. 2014;67:56-64.

18. Office of Diversity and Outreach. Unconscious Bias. UCSF. N.d. https://diversity.ucsf.edu/resources/unconscious-bias. Accessed May 7, 2019.

19. Multicultural Centre for Women's Health. Intersectionality Matters: A new resource for preventing violence against women. http://www.mcwh.com.au/downloads/Intersectionality-Matters-Guide-2017.pdf. Published 2017. Accessed February 4, 2019.

20. Guinier, L., Torres, G., & Guinier, L. The Miner's Canary: enlisting race, resisting power, transforming democracy. Harvard University Press. 2009.

21. Hankivsky, O., & Cormier, R. Intersectionality: Moving Women's Health Research and Policy Forward. Vancouver: Women's Health Research Network. Published 2009. Accessed February 4, 2019.

22. The Opportunity Agenda. Ten Tips for Putting Intersectionality into Practice. https://opportunityagenda.org/explore/resources-publications/ten-tips-putting-intersectionality-practice. Published 2017. Accessed February 4, 2019.

23. Government of Canada, S. of W. C. Gender-based Analysis Plus (GBA+) - Take the GBA+ course - the Department for Women and Gender Equity. 2018. https://cfc-swc.gc.ca/gba-acs/course-cours-en.html. Accessed February 4, 2019.

24. Weber, L. Fostering Positive Race, Class, & Gender Dynamics in the Classroom. Women's Studies Quarterly 18 (Spring/Summer 1990). 1990;126-134.

25. CRIAW ICREF. Diversity through inclusive practice - the toolkit checklist. http://fnn.criaw-icref.ca/images/publications/41949d448c12f9a3b3055413e3ce04c9.pdf. Published 2014. Accessed February 4, 2019.

26. Ontario Council of University Libraries. Accessibility Information Toolkit for Libraries https://ocul.on.ca/accessibility/sites/default/files/OCUL%20Accessibility%20Toolkit%20-%20ENG%20-%20v2.0%20%28May%202014%29.pdf. Published May 2014. Accessed February 4, 2019.

27. Ryerson University. Hosting accessible events and meetings. N.d. https://www.ryerson.ca/accessibility/guides-resources/hosting-accessible-events-and-meetings/#pre-event-planning. Accessed February 4, 2019.

28. Thuerbach, C. This IS your grandfather's project management: Management Institute Building a trust-based team. PMI® Global Congress. Published 2014. Accessed February 4, 2019.

29. Mason, C. N. Leading at the Intersections: An Introduction to the Intersectional Approach Model for Policy & Social Change. New York: Women of Color Policy Network, New York University Robert F. Wagner. 2010. http://www.intergroupresources.com/rc/Intersectionality%20primer%20-%20Women%20of%20Color%20Policy%20Network.pdf. Accessed February 4, 2019.

30. Ontario Living Wage Network. Living Wage by Region. N.d. http://www.

Guide References

ontariolivingwage.ca/living_wage_by_region. Accessed November 22, 2019.
31. Becu, A. & Allan, L. Peer payment standards for short-term engagement. BC Centre for Disease Control. http://www.bccdc.ca/resource-gallery/Documents/Educational%20Materials/Epid/Other/peer_payment-guide_2018.pdf. Published February 2018. Accessed February 4, 2019.
32. Canadian AIDS Society. Peerology: A guide by and for people who use drugs on how to get involved. http://librarypdf.catie.ca/PDF/ATI-20000s/26521E.pdf. Published June 2015. Accessed August 15, 2019.
33. Strategy for Patient-Oriented Research. Considerations when paying patient partners in research. http://www.cihr-irsc.gc.ca/e/51466.html. Updated May 27, 2019. Accessed August 15, 2019.
34. CARFAC-RAAV. Minimum Recommended Fee Schedule. https://www.carfac.ca/tools/fees/. Accessed October 3, 2019.
35. Hamilton, C., et al. Workbook to guide the development of a Patient Engagement In Research (PEIR) Plan. Arthritis Research Canada. http://www.arthritisresearch.ca/wp-content/uploads/2018/06/PEIR-Plan-Guide.pdf. Published 2018. Accessed February 4, 2019.
36. Deverka, P. A., Lavallee, D. C., Desai, P. J., Esmail, L. C., Ramsey, S. D., Veenstra, D. L., & Tunis, S. R. Stakeholder participation in comparative effectiveness research: defining a framework for effective engagement. Journal of Comparative Effectiveness Research. 2012;1(2), 181-194.
37. Frank, F. & Smith, A. The Community Development Handbook: A Tool to Build Community Capacity. Human Resources Development Canada. 1999;71-72. http://publications.gc.ca/collections/Collection/MP33-13-1999E.pdf. Accessed February 4, 2019.
38. TV 2 Danmark. All that we share [Video]. Youtube. https://www.youtube.com/watch?v=jD8tjhVO1Tc. Published January 27, 2017. Accessed February 4, 2019.
39. Teaching Tolerance. Intersectionality 101 [Video]. Youtube. https://www.youtube.com/watch?v=w6dnj2IyYjE. Published May 18, 2016. Accessed February 4, 2019.
40. Rainbow Health Network. Training for Change: Practical Tools for Intersectional Workshops.
http://www.oaith.ca/assets/files/Publications/Intersectionality/Practical-tools-intersectional-workshops.pdf. Published 2010. Accessed February 21, 2019.
41. Government of Canada. Trauma and violence-informed approaches to policy and practice. Public Health Agency of Canada. https://www.canada.ca/en/public-health/services/publications/health-risks-safety/trauma-violence-informed-approaches-policy-practice.html. Updated February 2018. Accessed February 4, 2019.
42. Lavizzari, A. Intersectionality Toolkit. IGLYO. https://www.iglyo.com/wp-content/uploads/2017/04/Intersectionality-Research-Report.pdf. Published 2015. Accessed February 21, 2019.
43. Youth Mental Health Canada. People first language. https://ymhc.ngo/resources/people-first-language/. Accessed September 21, 2019.
44. Violence Against Women Lexicon. 2013. http://www.vawlearningnetwork.ca/learning-network-resources/violence-against-women-lexicon. Accessed February 21, 2019.
45. The UN convention on rights of persons with disabilities. 2012. https://www.un.org/disabilities/documents/convention/convention_accessible_pdf.pdf Accessed February 21, 2019.
46. Braveman P, Gruskin S. Defining equity in health. J Epidemiol Community Health. 2003;57(4):254–258. doi:10.1136/jech.57.4.254
47. Bhopal, R. Glossary of terms relating to ethnicity and race: For reflection and debate. Journal of Epidemiology and Community Health. 2003;58:441-445.
48. Definitions of sex and gender. http://www.cihr-irsc.gc.ca/e/47830.html. Updated June 2015. Accessed February 21, 2019.
49. The 519 Glossary of Terms n.d. https://www.the519.org/education-training/glossary. Accessed February 21, 2019.
50. Disability Studies. Defining Impairment and Disability. n.d. https://disability-studies.leeds.ac.uk/wp-content/uploads/sites/40/library/Northern-Officers-Group-defining-impairment-and-disability.pdf. Accessed September 21, 2019.
51. Social Justice - Privilege and Oppression. n.d. https://career.arizona.edu/sites/career.arizona.edu/files/social_justice/story_html5.html. Accessed February 21, 2019
52. McIntosh, P. White privilege: Unpacking the invisible knapsack. 1988. https://files.eric.ed.gov/fulltext/ED355141.pdf?utm#page=43. Accessed February 21, 2019.
53. Wildman, S. M., & David, A. D. Language and silence: Making systems of privilege visible. Santa Clara L. Rev. 1994;35:881.
54. Johnson, A. G. Privilege, power, and difference. (2nd ed.). McGraw-Hill. 2006.
55. Canadian Mental Health Association. Stigma and Discrimination. https://ontario.cmha.ca/documents/stigma-and-discrimination/. Accessed September 21, 2019.
56. Centre for Addiction and Mental Health. Trauma. https://www.camh.ca/en/health-info/mental-illness-and-addiction-index/trauma. Accessed September 21, 2019.
57. Tokenism in patient engagement | Family Practice | Oxford Academic. n.d. https://academic.oup.com/fampra/article/34/3/290/2503177. Accessed February 21, 2019.
58. Ontario Human Rights Commission. Appendix 1 : Glossary of human rights terms. http://www.ohrc.on.ca/en/teaching-human-rights-ontario-guide-ontario-schools/appendix-1-glossary-human-rights-terms. Accessed February 4, 2019.
59. Simpson, J. Everyone Belongs. A Toolkit for Applying Intersectionality. Canadian Research Institute for the Advancement of Women (CRIAW). Published 2009. Accessed February 4,2019.
60. Shimmin C, Wittmeier K.D.M., Lavoie J.G., Wicklund E.D., Sibley K.M. Moving towards a more inclusive patient and public involvement in health research paradigm: the incorporation of a trauma-informed intersectional analysis. BMC Health Services Research. 2017;17:539.

Intersectionality & Knowledge Translation

Guide Sheet

Intersectionality

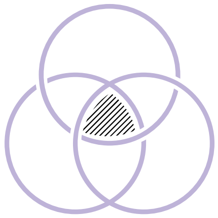

What is intersectionality?

Intersectionality* is a way of looking at the world that recognizes that people's experiences are shaped by a combination of social factors, including their gender, racialization, age, among others.[1-7] These experiences occur within and interact with connected systems and structures of power, such as sexism and racism.[1-7]

Note that there are various definitions of intersectionality and that they are evolving.

Taking an intersectional approach involves being inclusive and considering the unique experiences of those on your teams and in your communities.

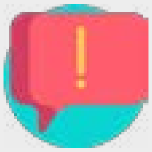

Please note: Taking an intersectional approach is needed to recognize the importance of individuals' social identities within the greater context of systems and structures of power. These reflect macro systems of privilege and oppression. Keep in mind that recognizing areas of advantage, disadvantage, and oppression may bring up feelings of confusion, guilt, distress, among others. It is okay to feel uncomfortable. There is a difference between feeling uncomfortable and unsafe.

What are intersecting categories?

Intersecting categories include age, gender identity, sex, and other aspects of one's lived experience. These aspects interact to form a person's identity (See Figure 1).[6-8] One's intersecting identities reflect larger systems of oppression/ privilege (e.g., sexism, ageism).[6-8]

A person's social identities shape their experiences in the world and how they view it (including the conscious and unconscious biases they hold).

You can learn more about how to consider your biases as you start to look at the knowledge translation project management steps.

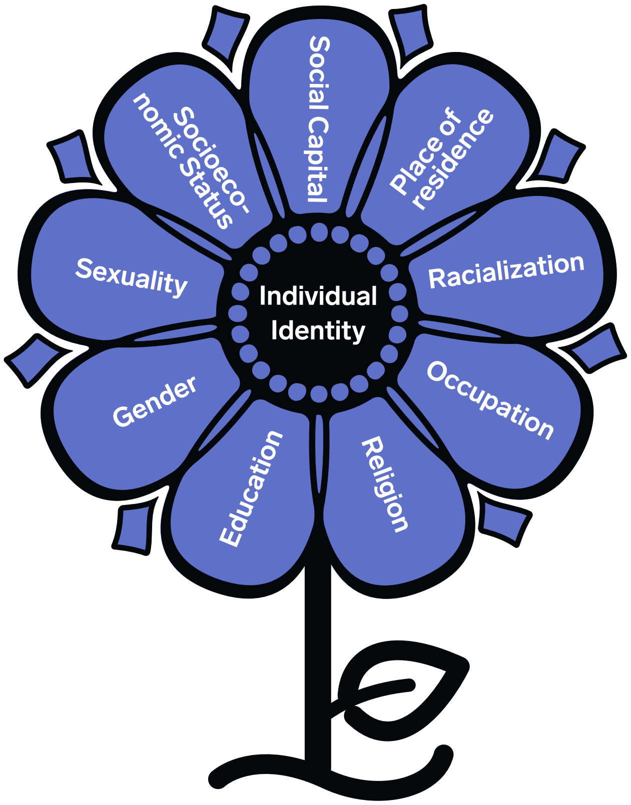

Figure 1. Visual representation of some intersecting categories.[6,9,10] The categories mentioned in this figure are not an exhaustive list.

Knowledge Translation

What is knowledge translation?

Knowledge translation (KT) is the process of moving evidence into health care practice.[11]

KT intervention developers are people who create KT interventions designed to improve health care.

For example, a KT intervention developer may design a KT intervention to change how often nurses encourage patients to exercise in long-term care homes. The KT intervention may include restructuring nurses' workflow and delivering in-person education sessions.

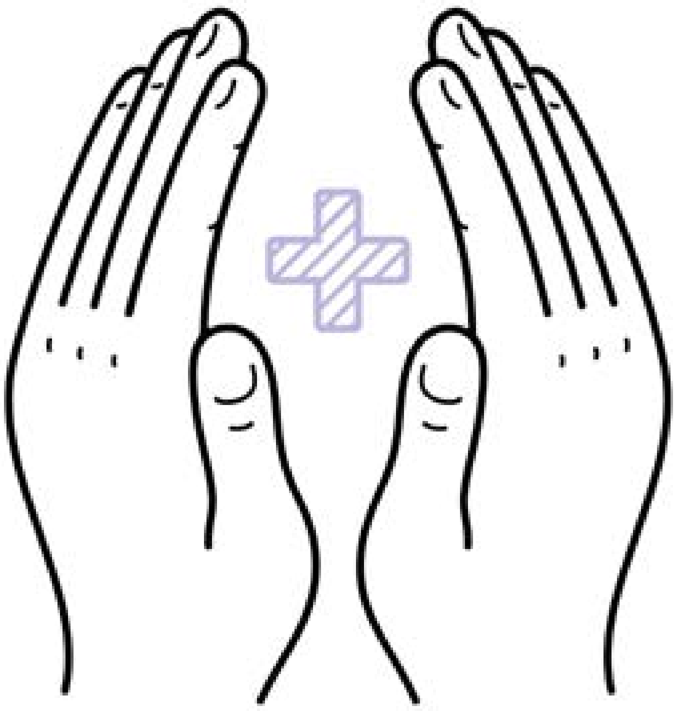

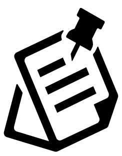

Before starting your KT project, consider the biases you may have as an individual and as a team. A bias is a preconceived judgment for or against a particular individual or group.[12]

How do you take an intersectional approach to KT?

Intervention developers can take an intersectional approach to KT by considering the dynamic nature of social identities and their interactions with social structures and systems that may oppress or privilege different groups.

These social identities and their interactions can be considered at all stages of the KT process. When doing this, you can think about the people designing (e.g., KT intervention developers) and receiving (e.g., clinicians) the KT intervention and those affected by it (e.g., patients).

References

1. Collins, P. H. Routledge. 1990. https://trove.nla.gov.au/version/21207078
2. Crenshaw, K. U. Chi. Legal F. 1989;139.
3. Crenshaw, K. The politics of law: A progressive critique. 1990;195.
4. Crenshaw, K. Stanford Law Review. 1991;43(6), 1241-1299. doi:10.2307/1229039
5. The Learning Network. Issue 15: Intersectionality. 2015. Accessed February 7, 2019.
6. Hankivsky, O., Grace, D., Hunting, G. et al. Int J Equity Health 13, 119 (2014) doi:10.1186/s12939-014-0119-x
7. Hankivsky, O. The Institute for Intersectionality Research & Policy, SFU. 2014;1-34. Accessed February 7, 2019.
8. City of Ottawa and City for All Women Initiative. CAWI. 2015. Accessed February 6, 2019.
9. PROGRESS-Plus. Cochrane Equity Methods. Accessed November 12, 2019.
10. O'Neill, J., et al. Journal of Clinical Epidemiology. 2014;67:56-64.
11. Government of Canada. 2010. http://www.cihr-irsc.gc.ca/e/40618.html. Accessed May 28, 2019
12. Office of Diversity and Outreach. UCSF. https://diversity.ucsf.edu/resources/unconscious-bias. Accessed May 7, 2019.

Intersectionality & Knowledge Translation (KT)

Reflection Workbook

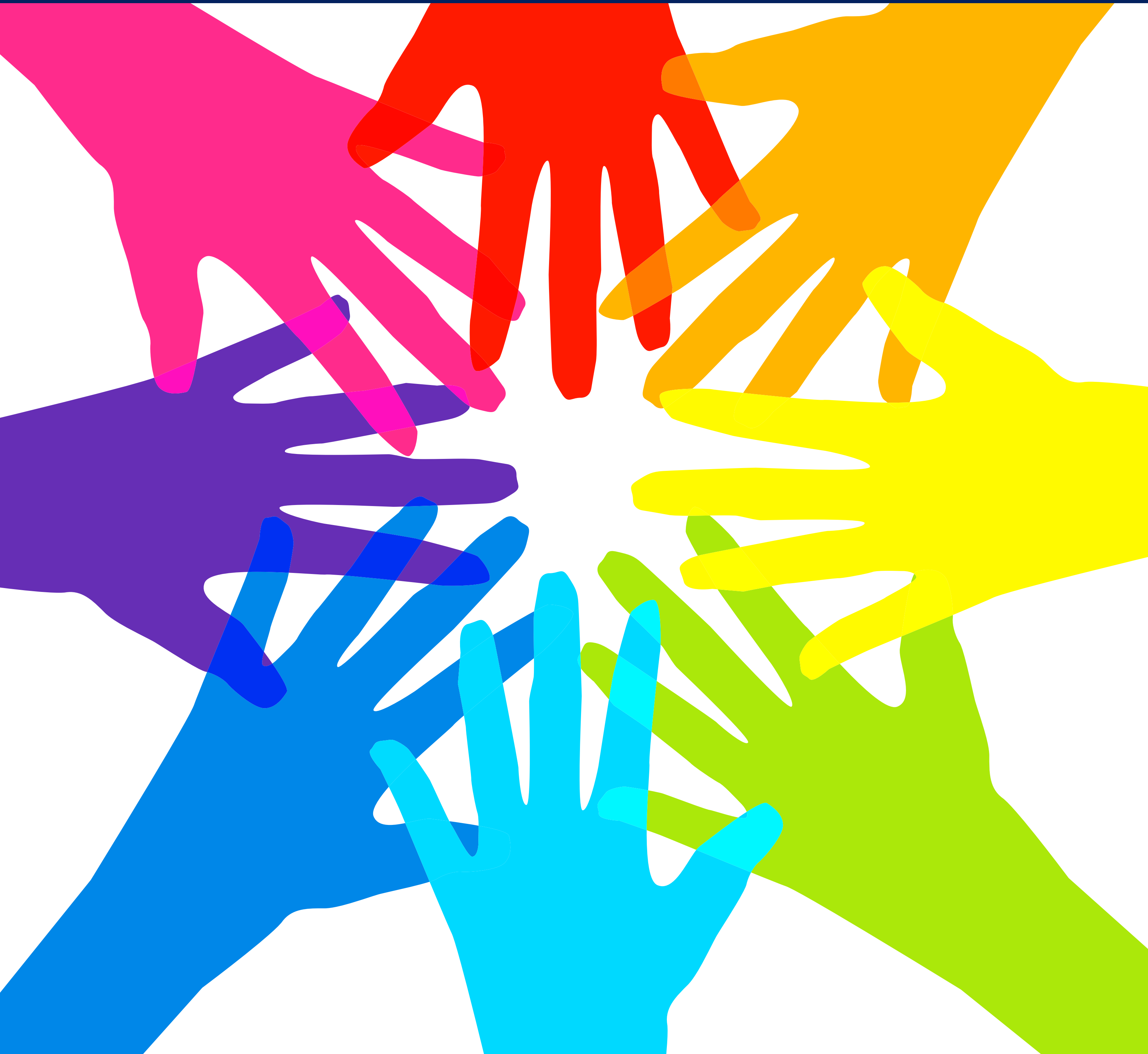

Table of Contents

Introduction 3
Summary of background on the reflection workbook

Self-Reflection: Where am I situated? 5
Reflections on your beliefs and behaviours

Activity: Exploring unconscious bias 6

Who is on the implementation team? 7
Considerations for including voices that reflect a range of intersecting categories

Activity: What are we talking about? Who are we talking with? 8

Resources for team engagement 10

Reflection: Problem, gap, and practice change 12
Overview of how to navigate the next steps of reflections

Identifying the problem 13
Find the difference between the current state and the desired state

Defining the evidence-to-practice gap 14
Determine whose voices are prioritized and how decisions are made

Selecting the practice change 15
Identify who is expected to change their behaviour

Reflection: Appraising evidence 16
Evaluate the quality of the data used in this process

Optional activity: Adapting critical appraisal tools 18

Next steps 19

Appendix A: Project limitations 20

Appendix B: Optional activity 21
Reflecting on surveying intersecting categories

References 22

Introduction

Summary of background on the reflection workbook

Who is this workbook for?

This workbook is for knowledge translation (KT) intervention developers. KT is the process of moving evidence into health care practice.[1] KT intervention developers are people who create KT interventions designed to improve health care.

For example, an intervention developer may design a KT intervention to encourage physiotherapists to use a patient physical activity program. The KT intervention may include restructuring physiotherapists' workflow and delivering in-person education sessions.

KT intervention developers come from many different fields. To design more effective interventions, they can take an intersectional approach.

Why should I use this workbook?

This workbook guides KT intervention developers through reflection questions about intersectionality.

By applying an inclusive and equitable lens to KT interventions, you can design more effective interventions that address the complex realities of the people you work with.[2-5]

What is the purpose of this workbook? When do I use this workbook?

This workbook can be completed throughout a KT project's life cycle but is most applicable to the initial stages of a KT project, as conceptualized in the Knowledge-to-Action (KTA) Cycle[6]:

- Identifying a problem;
- Defining evidence-to-practice gap(s); and
- Selecting practice change(s)

You can use this workbook with any of the models, theories, and frameworks you would use to guide a KT project, making it easy to integrate.

This tool is part of a set of tools that help us take an intersectional approach when doing KT. Consult the tools below for more information on key topics.

- Running a KT project with an intersectional approach: Intersectionality Guide.
- Conducting an intersectional barriers and facilitators assessment: Guide for Common Approaches to Assessing Barriers and Facilitators to Knowledge Use.
- An intersectional approach to selecting and tailoring KT interventions using the results of a barriers and facilitators assessment: Selecting and Tailoring KT Interventions Workbook.

How do I use this workbook?

This workbook contains reflection questions that are meant to be completed individually. Fill in the blank boxes following each question with your thoughts on the reflection question.

It also contains activities and resources. It is meant to prompt reflection; it is not meant to be prescriptive.

The time it takes to respond to each question will vary will vary from person to person. In general, each question takes approximately 10–25 minutes to answer. This may seem like a long time, but this work will help us create KT interventions that consider diverse human experiences.

Revisit your responses as you work through a project. Once the project is complete, reflect on your previous responses. Consider how these insights can apply to other and future KT projects.

If everyone on the team is comfortable with it, individuals may choose to share their responses to the reflection questions. Before deciding to share responses, teams should reflect on power and team dynamics (see resources in the Intersectionality Primer). If the team shares responses, consider pooling responses to keep individual responses anonymous.

Who made this workbook?

This tool was collaboratively developed in an iterative fashion by an interdisciplinary team of KT scholars, KT intervention developers, intersectionality scholars, and adult education experts.

Project limitations

See Appendix A for a project limitation statement.

This tool cannot be broadly applied to Indigenous Peoples, and there may be more culturally appropriate models, theories, and frameworks that are useful to consider when conducting projects that involve Indigenous communities.

Key terms

Intersectionality* is a way of looking at the world that recognizes that people's experiences are shaped by a combination of social factors, including their gender, racialization, age, among others.[7-13] These experiences occur within and interact with connected systems and structures of power, such as sexism and racism.[7-13]

Note that there are various definitions of intersectionality and that they are evolving.

Intersecting categories include age, gender identity, sex, and other aspects of one's lived experience. These aspects interact to form a person's identity (See Figure 1).[3,12,13] One's intersecting categories reflect larger systems of oppression/privilege (e.g., sexism, ageism).[3,12,13]

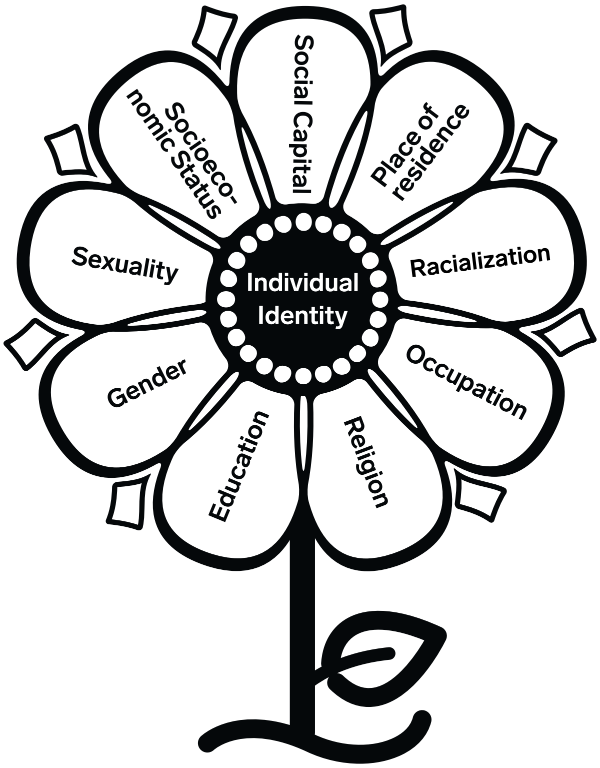

Figure 1. Visual representation of some intersecting categories.[12,14,15] The categories mentioned in this figure are not an exhaustive list.

How do I take an intersectional approach to KT?

Intervention developers can take an intersectional approach to KT by considering the dynamic nature of intersecting categories and their interactions with social structures and systems. Their interactions with social structures and systems may oppress or privilege different groups.

These intersecting categories and their interactions can be considered at all stages of the KT process. When doing this, you can think about the people designing (e.g., KT intervention developers) and receiving (e.g., clinicians) the KT intervention and those affected by it (e.g., patients).

By taking an intersectional approach in your work, you can identify the root causes of inequities, overcome conceptual gaps, and consider complex factors together to create an effective KT intervention.[2-5]

> **"There is no such thing as a single-issue struggle because we do not live single-issue lives."[16]**
>
> - Audre Lorde, 1984, p.138

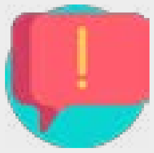

Please note: Taking an intersectional approach is needed to recognize the importance of individuals' social identities within the greater context of systems and structures of power which reflect macro systems of privilege and oppression. Keep in mind that recognizing areas of advantage, disadvantage, and oppression may bring up feelings of confusion, guilt, distress, among others. It is okay to feel uncomfortable. There is a difference between feeling uncomfortable and unsafe.

Self-Reflection: Where am I situated?

Reflections on your beliefs and behaviours

Before starting the project, it is important to reflect on your own beliefs and behaviours. Like the people you work with and the populations you support, your individual identity and perceptions are shaped by your intersecting categories and their interaction with systems and power structures.[13]

This self-reflection is designed to be completed by everyone on the implementation team, including those who join the team throughout various project stages.

An individual's place in society is based on their identity. One's identity includes intersecting categories like age, gender identity, socioeconomic status, disability, and geographic location among other intersecting categories. This place in society relates to processes of privilege (e.g., socioeconomic privilege) and disadvantage/oppression (e.g., sexism)[13]:

- Privilege is a special right or advantage available to a particular person or group of people.[17] It can be earned or unearned.[17]
- Disadvantage is when a special right or advantage is unavailable to a particular person or group.[17]
- Oppression occurs when a person or a group faces systematic disadvantages, mistreatment, exploitation, and abuse.[17]
- An individual can simultaneously experience privilege and disadvantage/oppression.[17] For example, a Canadian physician who self-identifies as a person of colour may simultaneously experience privilege (through their respected role as a physician) and disadvantage/oppression (through racism).

Question 1: What intersecting categories make up your identity?[18]

Use the empty intersectionality flower below to identify the intersecting categories that you think make up your identity.

> **"Reflexivity acknowledges the importance of power at the micro level of the self and our relationships with others, as well as the macro level of society. It recognizes the multiple truths and a diversity of perspectives, while giving extra space to voices typically excluded from 'expert' roles"[19,20]**
>
> - Shimmin et al. (2017)

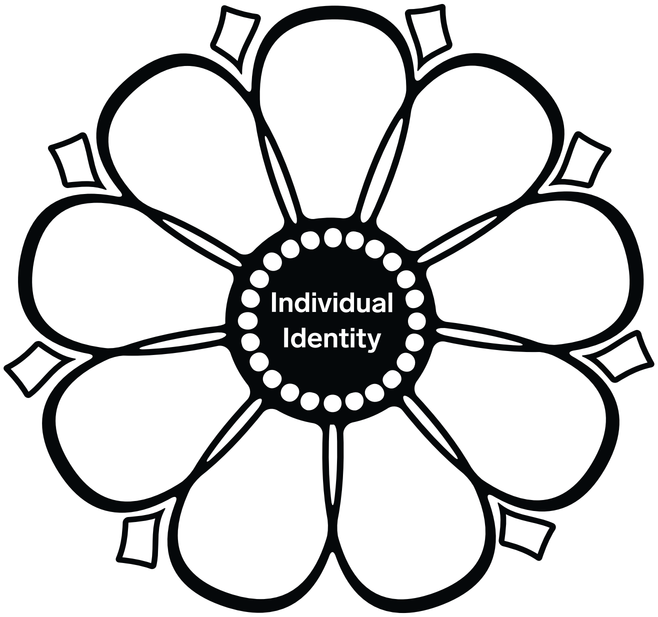

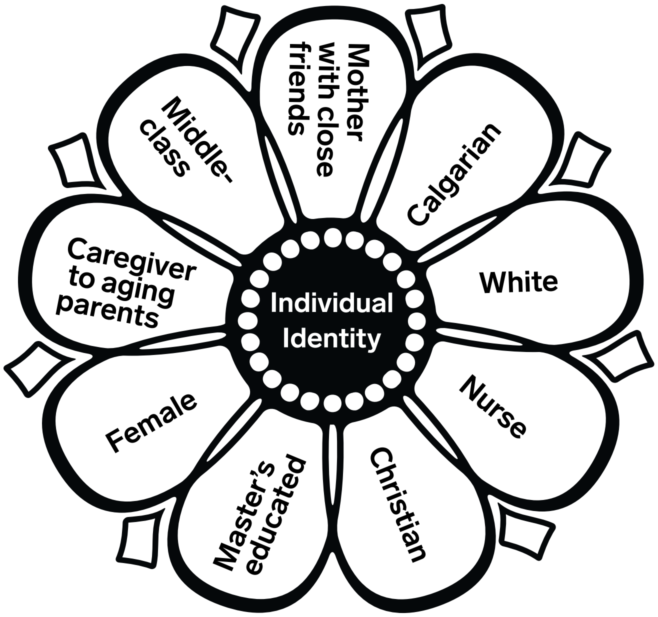

Example response to reflection Question 1

Activity: Exploring unconscious bias

Complete the following self-reflection questions individually.

Bias is a preconceived judgment for or against a particular individual or group.[21] There are multiple types of bias:

- Conscious bias (also known as explicit bias) is within one's conscious awareness[21];
- Unconscious bias (also known as implicit bias) is beyond one's conscious awareness.[21]

Everyone holds biases; biases about identities and social groups stem from the tendency for people to categorize individuals.[21]

An example of an unconscious bias would be the unintentional differences in how a clinician interacts with patients depending on their age, race, and whether they speak English as a first language.

To explore and try to mitigate your biases, review these free tests and courses:

- **Harvard Project Implicit**
  https://tinyurl.com/6yyyc[22]
- **Government of Canada - Unconscious Bias Training Module**
  https://tinyurl.com/yacj5ao3[23]
- **EdX - Unconscious Bias: From Awareness to Action**
  https://tinyurl.com/yxk5lmb2[24]

Question 2: Reflecting on your response to question 1, how do your intersecting categories impact your place in society?[18]

Question 3: How do your identities relate to the project's topic area? How might your place in society impact your work on this project?[18]

For example, consider your personal and professional experiences, values, and interests.[18,19]

Who is on the implementation team?

Considerations for including voices that reflect a range of intersecting categories

An implementation team is the group of people responsible for designing and delivering a KT intervention. You may not be able to select members of an implementation team. However, you can reflect on the voices that are and are not represented on the team. You can also reflect on how to better incorporate voices that represent a range of intersecting categories.

In addition, you can reflect on who is on the team throughout the project's life cycle (see Questions 4–8).

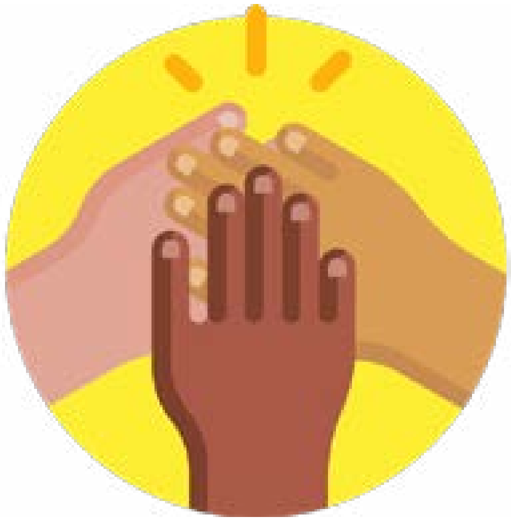

Question 4: What does an inclusive approach mean to you? What inclusive approaches have been used by your team, in your organization, or in other organizations? What about these approaches worked well and what did not work well?[18] Note that not all teams or organizations take an inclusive approach.

- Instead of the term "inclusive," terms like "diversity" and "equity" may be more appropriate for your context. Refer to the Intersectionality Guide for a more detailed understanding of these terms.
- "Diversity" refers to all differences within, between, and among a population.[25]
- "Equity" is a process whereby individuals are given different supports appropriate to their needs so that they have access to equal opportunities.[26]
- The purpose of reflecting on what you and the project team consider to be an "inclusive approach" is to explore what it means to work together by respecting different perspectives, experiences, and backgrounds.
- Consider using the following toolkits in your reflection: **Canadian Centre for Diversity and Inclusion Toolkits:** https://tinyurl.com/yxkoqd4z[27]

Activity: What are we talking about? Who are we talking with?

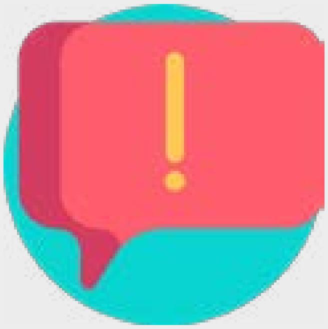

The following table can be completed individually by project team members.

Individual results may be shared with the team, but do not force team members to share their responses. Before beginning the exercise, it is important for the team to set ground rules for sharing these results and to clarify what actions may or may not be taken based on the information shared.

Think about the project team and rate your level of agreement with the following prompts[28]*:*

Gender idenities are regularly discussed/ considered as part of our work.					
Our team has links with organizations doing anti-oppression work relating to gender (e.g., The Canadian Centre for Gender and Sexual Diversity).					
Racial and ethnic identities are regularly discussed/considered as part of our work.					
Our team has links with organizations working in a range of racial and ethnic communities (e.g., Calgary Multicultural Centre).					
Disabilities are regularly discussed/ considered as part of our work.					
Our team has links with organizations working in the disability space (e.g., Tangled Art + Disability).					
Socioeconomic statuses are regularly discussed/considered as part of our work.					
Our team has links with organizations working to address socioeconomic disparities (e.g., Ontario Living Wage Network).					
Include additional prompts for categories that are relevant to a KT project, team, and community.					

Question 5: Who is the patient, healthcare provider, and community population affected by the project topic area? What would they want to get out of the project topic area? How do you plan to get them involved?[29]

Here are examples of how team members can balance power [29,30]:

- ☐ Include multiple individuals to represent a particular group (e.g., include five patient partners instead of one) [29,30]
- ☐ Employ trained moderators to focus on deliberation of ideas (i.e., ensure that no one voice is prioritized over the rest of the group) [29,30]
- ☐ Provide a range of supports (e.g., information support, training) [29,30]
- ☐ Create space for informal social interaction (i.e., build relationships among team members) [29,30]

Question 6: What are the real and perceived power differences on the team?[19,29]

- Consider how the team can become more aware of potential power differences or inequities (e.g., are there perceived power differences between team members who have many years of work experience compared to those who do not? Are there perceived power differences between those who speak with an accent and those who do not?).
- Consider how I can encourage team members to challenge ideas or renegotiate power.[19]
- Consider your response to question 3 on defining an "inclusive approach".

Resources for team engagement

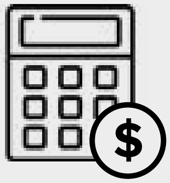

Team engagement takes time and resources. To estimate a budget for team engagement, consult the free downloadable budget tool available here:

George & Fay Yee Centre for Healthcare Innovation - Budgeting for Engagement:
https://tinyurl.com/y2x78oww[31]

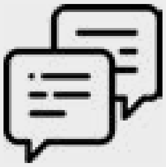

To learn more about engaging patients and members of the public, consult this resource:

George & Fay Yee Centre for Healthcare Innovation - How to Engage Patients & Public in Health Research:
https://tinyurl.com/y25nz3f7[32]

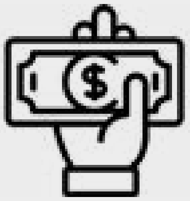

For more considerations when paying patient partners, please visit the following:

Canadian Institutes of Health Research - Considerations when paying patient partners in research:
https://tinyurl.com/y6ktlxgd[33]

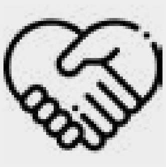

To explore an interactive guide to developing a meaningful patient/community engagement strategy, visit the following:

Arthritis Research Canada - Workbook to guide the development of a Patient Engagement In Research (PEIR) Plan:
https://tinyurl.com/yxhj2h3f[29]

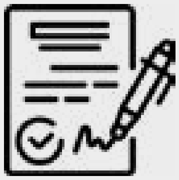

As an example of how to create policies on team engagement, please visit:

SPOR Evidence Alliance- Policies and Procedures:
https://tinyurl.com/vtk3teg[34]

Question 7: Reflect on whether everyone who could be on the team has been asked if and how they would like to be involved. Think about how different perspectives that represent a range of intersecting categories have been examined.

- What are the intersecting categories of the health care providers who work in this area of health? Are they reflected on the implementation team?[18]
- If the intersecting categories of the health care providers and community are not diverse, consider the reasons for this (e.g., because the health topic affects only certain people, because gender roles have specified who are considered "leaders" in the organization).
- Consider ways to engage people with a range of perspectives, such as patients, families, caregivers, communities, policy makers, trainees, project funders, and organizational leadership.

Question 8: Does your team reflect the makeup of the patient, community, and health care providers that experience the project topic?[30]

- [ ] What are some potential challenges people with lived experience on this topic (including families and communities) might face when getting involved in project work? Consider how these challenges may be mitigated.[20]
- [ ] Consider your response to question 3 on defining an "inclusive approach." What does the implementation team need to do to create an inclusive environment for everyone involved, including patients and community members? For example, budget for accommodations and supports for all team members (e.g., translators, caregiving, meals) in advance.

Reflection: Problem, gap, and practice change

Overview of how to navigate the next steps of reflections

Problems are discrepancies between a current state and a desired state.

For example, in the late 2000s, the Division of Geriatric Medicine at the University of Toronto, along with collaborators, identified challenges related to geriatric care.[35] Through quality improvement initiatives, the group discovered that the current state of geriatric care fell short of the desired state. The identified problem was hospital-induced immobility in older adults.

Once you identify a problem area, you can define the evidence-to-practice gap.

An evidence-to-practice gap is the difference between what we know works and what happens in practice. For example, the Geriatric Medicine team reviewed evidence and found that without mobilization, older patients lose 1% to 5% of their muscle strength each day they spend in the bed and in the hospital.[35] However, the team discovered that current rates of mobilization were very low for older patients admitted to acute care hospitals.[35] Accordingly, there was an evidence-to-practice gap between the evidence in favour of mobilization and the current practice, which did not readily incorporate mobilization.

Once you define an evidence-to-practice gap, you can identify the practice change that can minimize the gap.

A practice change is something that can minimize the identified evidence-to-practice gap.

For example, to increase rates of mobilization among older patients, practice changes (identified from credible evidence by the Geriatric Medicine team) can include using progressive, scaled mobilization and assessing mobility within 24 hours of a patient's admission.[35]

To read more about this example, visit
MOVE Canada - The MOVE Program:
https://tinyurl.com/y2kd974z[35]

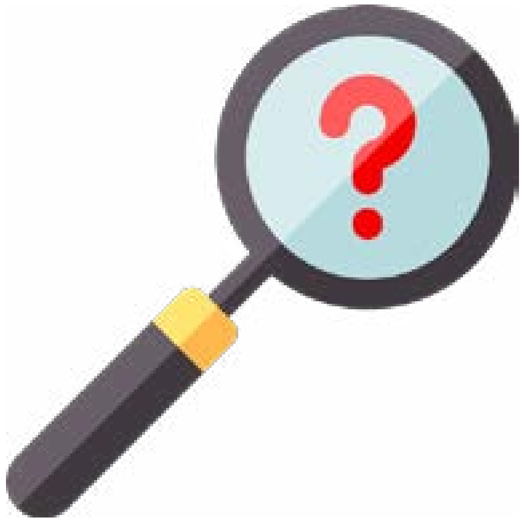

Identifying the problem

Find the difference between the current state and the desired state

Question 9: Whose point of view is reflected when defining the problem? For example, is it the Chief Executive Officer or the nurse who has prioritized a specific problem as the focus of the KT project?

- ☐ Is the problem a priority for the population affected (e.g., adults 65+ years)? Consider the KT project as a co-creation activity with all team members.
- ☐ Who may gain and who may lose if this problem is addressed?[30]
- ☐ Refer to your definition of an "inclusive approach." Consider how you can incorporate different perspectives at this stage.

Question 10: What are the information gaps in the problem area? How can these gaps be filled? Information gaps are areas where you do not have complete knowledge.

- ☐ Speak with those who experience the area of health (e.g., patients) and those who work in it (e.g., providers). Do I have knowledge about their lived experience with the topic?
- ☐ Consider if information gaps are similar for different demographic groups. Do people of different ethnicities experience the problem at similar rates? Is there information regarding some intersecting categories but not others?
  - Document and disseminate information about knowledge gaps pertaining to underrepresented perspectives. Disseminating what information you do not know can help other practitioners identify what assumptions you have made and the limitations of your work. Communicating this information is also helpful for researchers to identify research questions.
- ☐ Review quantitative and qualitative data to seek evidence on potential information gaps.

Defining the evidence-to-practice gap

Determine whose voices are prioritized and how decisions are made

Question 11: Who decides which evidence-to-practice gaps is prioritized?

- ☐ Be clear about whose behaviour an evidence-to-practice gap is reflecting: is it the health professional, the patient, community and/or another group?
- ☐ Consider again, who may gain and who may lose if a particular gap is prioritized?[30]
- ☐ Consider the potential for bias depending on who is defining the problem (e.g., a CEO and a nurse may identify different problems to prioritize).
- ☐ How will decisions be made? What methods will you and the team use in the prioritization process so that all voices are heard?
- ☐ Have those who will be affected by the practice change been involved in decision-making?

> “**The application of an intersectionality lens at this stage helps our team really think about the population we are supporting… before getting lost in the complex decision-making steps that take place in the early stages of intervention development. We see limitations in what we don’t know from the research on how the problem is being experienced across the diverse populations we support. This prompts us to plan for how we will fill these gaps moving forward.**”
>
> - Andrea Chaplin, Evaluation Specialist,
> Public Health Ontario

Selecting the practice change

Identify who is expected to change their behaviour

There is often a range of practice changes available to bridge an evidence-to-practice gap. Each practice change will affect those who are expected to change their practice/behaviour (e.g., nurses) and those who are affected by the change in practice/behaviour (e.g., patients) in various ways.

Question 12: Of the practice changes under consideration, who is expected to change their behaviour and "do" the practice changes? This "who" could be a health professional the patient, the community, and/or another group.

- ☐ Consider general barriers and facilitators to the practice change at this stage.

Question 13: a) Think about the group expected to change their behaviour (e.g., nurses). What intersecting categories might group members have? What intersecting categories may be relevant to the practice change? Write each relevant intersecting category within each petal of the blank flower below.

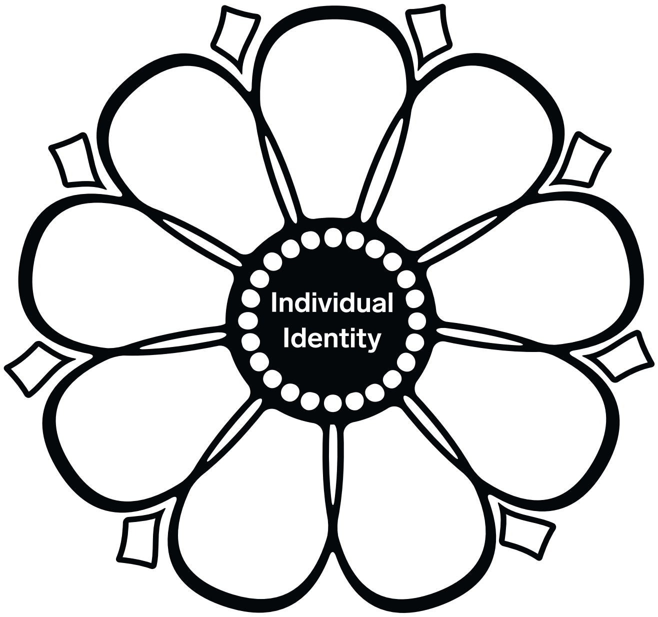

Question 13: b) Think about the group affected by the practice change (e.g., patients). What intersecting categories might group members have? What intersecting categories may be most relevant? Write each relevant intersecting category within each petal of the blank flower below.

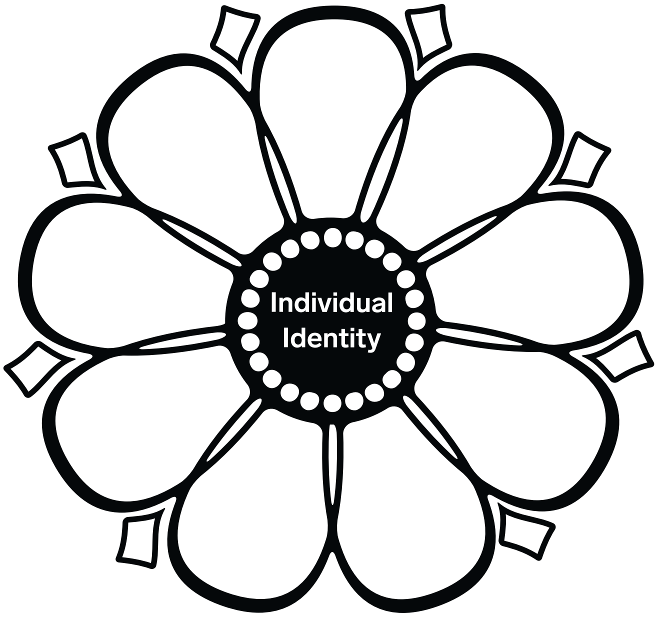

Reflection: Appraising evidence

Evaluate the quality of the data used in this process

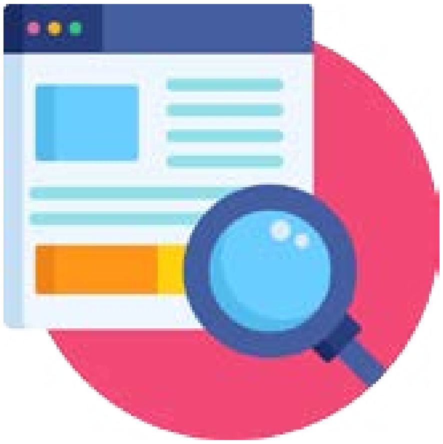

Research gaps are areas with insufficient or absent information. These gaps limit the ability to reach reasonable conclusions or decisions.

You can critique the research used to identify the problem, define the evidence-to-practice gap, and select the practice change.

Relevant evidence includes published evidence and the experience of those living and interacting with the problem, the evidence-to-practice gap, and the practice change. Be sure to evaluate lived-experience research, grey literature, and commentaries in addition to evidence syntheses.

When appraising evidence, consider the following:

Question 14: What information do I have? What information do I wish I had? Who might have this information? Who should I talk to about this?

- ☐ Consider supplementing available data with additional indicators from other sources (e.g., program evaluations, qualitative studies, lived-experience commentaries, strategic reports) to better understand different perspectives.
  - Look for ways to avoid categorizing groups with binaries (e.g., man or woman).[26]
- ☐ What do I consider to be "credible" evidence? From our team's perspective, what makes evidence "credible"?
- ☐ Document and disseminate information on knowledge gaps about underrepresented perspectives. Communicating this information is helpful for researchers to identify questions they can help answer.

Question 15: Critically assess the data

- ☐ Compare data from an internal organization survey to data from a national public database).
- ☐ How old are the data?
- ☐ What is the data source?
- ☐ Are the data reliable?
- ☐ Are the data valid?
- ☐ Does the data include binaries?
    - For example, are the data presented as “females” and “males”?
- ☐ If people are excluded, does it make sense why?
- ☐ Does the evidence identify and consider intersecting categories in a fair and sensitive way?
    - Does that data include stereotypes or assumptions?

Optional activity: Adapting critical appraisal tools

Critical appraisal is a process of assessing evidence by examining its source, study design quality, risk of bias, trustworthiness, relevance to a particular context, and other characteristics. Critical appraisal can be done individually or as a group. Individual results may be shared with the team.

Example adaptation of Kuper et al.'s qualitative research critical appraisal questions with an intersectional approach.[36] Intersectionality enhancements are italicized.

- ☐ Was the sample used in the study appropriate for the research question?[36]
  - ***Who asked the research question? Were those impacted by the research (e.g., people with lived experiences) involved in defining the research question?***
- ☐ Were the data collected appropriately?[36]
  - ***What would be "appropriate" for the population impacted by the research?***
  - ***Was the sample size sufficiently large to capture the intersection of multiple intersecting categories?***[19]
- ☐ Were the data analyzed appropriately?[36]
  - ***Does the research identify and consider intersecting categories in a fair and sensitive way?***
  - ***Is the methodology based on stereotypes or assumptions?***
- ☐ Can I transfer the results of this study to my own setting?[36]
  - ***Are the intersecting categories represented in the study similar to those in the population we are working with?***
- ☑ Does the study adequately address potential ethical issues, including reflexivity?[36]
- ☐ Overall, is it clear what the researchers did?[36]

We can adapt existing critical appraisal tools to incorporate an intersectional approach. If you or your organization prefers to use a certain critical appraisal tool, please consider how intersectionality could be incorporated into this tool.

Examples of critical appraisal tools (without intersectionality enhancements):

Joanna Briggs Institute Critical Appraisal Tools:
https://tinyurl.com/y32xg8ln[37]

Critical Appraisal Skills Programme - CASP Appraisal Checklists:
https://tinyurl.com/y7qx99mq[38]

Next steps

Take this moment to reflect on your responses. Think about how you can apply these considerations to other projects you are working on or any future ones.

You may choose to share your responses with other team members as long as everybody is comfortable with sharing their own. Reflecting on how others perceive the KT project helps you understand different perspectives that you did not originally consider.

For other stages of a KT project's life cycle, use these tools to integrate an intersectional approach:

- ☐ Running a KT project with an intersectional approach: Intersectionality Guide.
- ☐ Conducting an intersectional barriers and facilitators assessment: Guide for Common Approaches to Assessing Barriers and Facilitators to Knowledge Use
- ☐ An intersectional approach to selecting and tailoring KT interventions using the results of a barriers and facilitators assessment: Selecting and Tailoring KT Interventions Workbook.

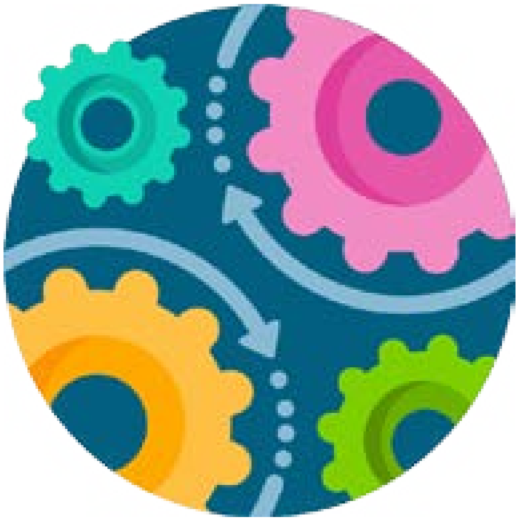

Appendix A: Project limitations

We acknowledge that the work of our Canadian Institutes of Health (CIHR)-funded team grant was conducted on unceded lands that were the traditional territories of many people, including the Algonquin, Cree, Dakota, Dene, Huron-Wendat, Mississaugas of the Credit River, and the Musqueam Peoples, and on the homeland of the Métis Nation. We acknowledge the harms of the past and the harms that are ongoing. We are grateful for the generous opportunities to conduct work on these lands.

In 2017, the CIHR launched an opportunity for team grants in gender and KT. This opportunity (sponsored by the Institute of Gender and Health) was developed to recognize that the field of KT had yet to thoughtfully integrate gender into its research agenda. The objectives of the CIHR team grant competition were to generate evidence about whether applying sex- and gender-based analysis to KT interventions involving human participants improves effectiveness, thereby contributing to improved health outcomes; contribute to a broader knowledge base on how to effectively and appropriately integrate gender into KT interventions; and facilitate the consideration and development of gender-transformative approaches in KT interventions.

In response to this call, we submitted a grant aimed at helping KT intervention developers use an intersectional approach when designing and implementing interventions to address the needs of older adults. We received feedback from the CIHR peer review committee that substantial concern was raised about our focus on intersectionality. In particular, the Scientific Officer's notes described that the focus on intersectionality would dilute the focus on gender and needed to be reconsidered. A meeting was subsequently held with the successfully funded team and this issue was raised again. We acknowledge the limitation that our intersectional approach comes at the expense of a minimized focus on gender. However, because intersecting categories, such as gender and age, are experienced together, we ultimately elected to use an intersectional approach as it encapsulates the lived experience of those we aim to impact.

A more significant limitation of our work is that we did not include First Nations, Inuit, and Métis community members in the grant proposal. As such, their needs and perspectives were not included in the research grant and, consequently, funded activities. Our team did not have established relationships or expertise in this area and as such, we felt it was inappropriate for our team to work on a grant in this area.

We strongly believe that consideration of gender and KT for Indigenous Peoples should be a primary focus of a distinct team grant.

There are established best practices for community engagement with First Nations, Inuit, and Métis Peoples that begin with principles of collaboration, which take time to develop and must not be tokenistic. The principles for collaboration should ensure authentic engagement, shared respect, trust, and commitment to ensure long-term, mutually empowered relationships. These principles should also ensure that the research-related priorities meet the needs, perspectives, and expectations of the First Nations, Inuit, and Métis Peoples. Indigenous Peoples have a long history of conducting research, and this tradition continues today with many Indigenous healers and scholars leading research in various areas. Indeed, there are many Indigenous scholars working in the KT field.

Because the team's work did not include First Nations, Inuit, and Métis Peoples and involve adhering to the principles that guide their engagement in research, the needs and considerations of these Peoples were not included in the work conducted in this team grant. As such, anyone who is considering using the outputs of this team grant needs to know that **they cannot be broadly applied to these Peoples and there may be other more culturally appropriate models, theories, and frameworks that are useful to consider**. Similarly, because this research focused on older adults (and in particular, chronic disease management in older adults) **it does not apply to children and youth**.

We believe that any KT intervention work needs to begin with engaging the appropriate community and is only applicable when those communities are engaged throughout the research enterprise. Moreover, intersectionality involves deep immersion in the lived experiences and priorities of those communities. As a result, KT work requires immersive work with various populations and not just key informants to ensure the work meets the needs of the relevant populations.

We thank and acknowledge Dr. Lisa Richardson, Co-Lead, Indigenous Health Education, Faculty of Medicine, University of Toronto, for her time and expertise in reviewing this statement.

Appendix B: Optional activity

Reflecting on surveying intersecting categories

To assess the project team members' intersecting categories, consider surveying the team.
Be mindful of the following:

- Why you are asking team members for this information. Commit to using this data for the purpose of recognizing which voices are at the table and which ones are not.
- How data will be used and not used (i.e., it will not be used in staffing decisions beyond the implementation project).
- The language/terminology you use to ask questions.
- How data will be collected.
- How data will be stored.
- Who has access to data (including risks of data breaches).
- Including "I prefer not to answer" as an option for each question. Do not force team members to disclose information they are not comfortable disclosing.

Curious about how other organizations survey the intersecting categories of their team members and project partners?

See the **Canadian Institutes of Health Research - Equity and Diversity Questionnaire**: https://tinyurl.com/yyq9ugad[39]

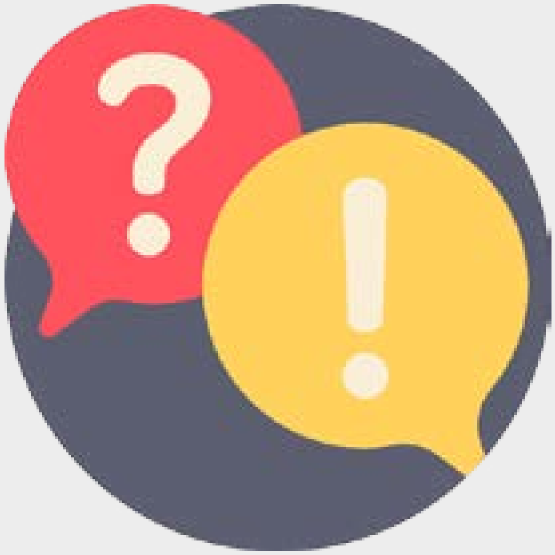

References

1. Government of Canada. Knowledge Translation in Health Care: Moving from Evidence to Practice - CIHR. 2010. http://www.cihr-irsc.gc.ca/e/40618.html. Accessed May 28, 2019
2. Tannenbaum, C., Greaves, L., & Graham, I.D. Why sex and gender matter in implementation research. BMC Med Res Methodol 2016;16:145. doi: 10.1186/s12874-016-0247-7
3. City of Ottawa and City for All Women Initiative. Advancing Equity and Inclusion – A Guide for Municipalities. CAWI. 2015. http://www.cawi-ivtf.org/sites/default/files/publications/advancing-equity-inclusion-web.pdf. Accessed February 6, 2019.
4. Public Health Agency of Canada. Key Health Inequalities in Canada: A National Portrait – Executive Summary. 2018. https://www.canada.ca/en/public-health/services/publications/science-research-data/key-health-inequalities-canada-national-portrait-executive-summary.html. Accessed February 6, 2019.
5. The Association for Women's Rights in Development. Intersectionality: A Tool for Gender and Economic Justice. Women's Rights and Economic Change. 2004. https://lgbtq.unc.edu/sites/lgbtq.unc.edu/files/documents/intersectionality_en.pdf. Accessed February 4, 2019.
6. Graham I.D., Logan J., Harrison M.B., Straus S.E., Tetroe J., Caswell W., Robinson N. Lost in Knowledge Translation: Time for a Map? J Contin Educ Health Prof. 2006; 26(1):13.
7. Collins, P. H. Black feminist thought: knowledge, consciousness, and the politics of empowerment. Routledge. 1990. https://trove.nla.gov.au/version/21207078
8. Crenshaw, K. Demarginalizing the intersection of race and sex: A black feminist critique of antidiscrimination doctrine, feminist theory and antiracist politics. U. Chi. Legal F. 1989;139.
9. Crenshaw, K. A Black feminist critique of antidiscrimination law and politics. The politics of law: A progressive critique. 1990;195.
10. Crenshaw, K. Mapping the Margins: Intersectionality, Identity Politics, and Violence against Women of Color. Stanford Law Review. 1991;43(6), 1241-1299. doi:10.2307/1229039
11. The Learning Network. Issue 15: Intersectionality. 2015. http://www.vawlearningnetwork.ca/sites/vawlearningnetwork.ca/files/Intersectioanlity_Newsletter_FINAL2.pdf. Accessed February 7, 2019.
12. Hankivsky, O. Intersectionality 101. The Institute for Intersectionality Research & Policy, SFU. 2014;1-34. http://vawforum-cwr.ca/sites/default/files/attachments/intersectionallity_101.pdf. Accessed February 7, 2019.
13. Hankivsky O, Grace D, Hunting G, et al. An intersectionality-based policy analysis framework: critical reflections on a methodology for advancing equity. International Journal for Equity in Health. 2014;13:119. doi:10.1186/s12939-014-0119-x.
14. PROGRESS-Plus. Cochrane Equity Methods. https://methods.cochrane.org/equity/projects/evidence-equity/progress-plus. Accessed November 12, 2019.
15. O'Neill, J., Tabish, H., et al. Applying an equity lens to interventions: Using PROGRESS ensures consideration of socially stratifying factors to illuminate inequities in health. Journal of Clinical Epidemiology. 2014;67:56-64.
16. Lorde, A. "Learning from the 60s," in SISTER OUTSIDER: ESSAYS & SPEECHES. Trumansburg, NY: Crossing Press, 1984: 138.
17. Grace D. When oppressions and privilege collide: a review of research in health, gender and intersectionality in late (post) modernity. Can J Hum Soc Sci. 2010;1(1):20-24.
18. Hankivsky O, Grace D, Hunting G, et al. ntersectionality-based policy analysis. An intersectionality-based policy analysis framework, 2012;33-45.
19. Shimmin C, Wittmeier KDM, Lavoie JG, Wicklund ED, Sibley KM. Moving towards a more inclusive patient and public involvement in health research paradigm: the incorporation of a trauma-informed intersectional analysis. BMC Health Serv Res. 2017;17(1):539. doi:10.1186/s12913-017-2463-1
20. Bolzan N, Heycox K, Hughes L. From pillar to post: women and social work studies in the 21st century. Aust Soc Work. 2001;54(1):67–79. doi: 10.1080/03124070108415265.
21. Office of Diversity and Outreach. Unconscious Bias. UCSF. N.d. https://diversity.ucsf.edu/resources/unconscious-bias. Accessed May 7, 2019.
22. Harvard EDU. Project Implicit. N.d. from https://implicit.harvard.edu/implicit/. Accessed March 7, 2019.
23. Government of Canada, I. C. Canada Research Chairs. 2018. http://www.chairs-chaires.gc.ca/program-programme/equity-equite/bias/module-eng.aspx?pedisable=false. Accessed March 7, 2019.
24. EdX. Unconscious Bias: From Awareness to Action. 2017. https://www.edx.org/course/unconscious-bias-awareness-action-catalystx-ub1x. Accessed March 7, 2019.
25. SGBA e-Learning Resource. Define Diversity. N.d. http://sgba-resource.ca/en/concepts/diversity/define-diversity/. Accessed May 13, 2019.
26. Government of Canada, Status of Women Canada. Gender-based Analysis Plus (GBA+) - Take the GBA+ course. September 26, 2018. https://cfc-swc.gc.ca/gba-acs/course-cours-en.html. Accessed March 7, 2019.
27. Canadian Centre for Diversity and Inclusion Toolkits. N.d. https://ccdi.ca/toolkits/. Accessed March 7, 2019.
28. The International Lesbian, Gay, Bisexual, Transgender, Queer & Intersex Youth and Student Organization (IGLYO). Intersectionality Toolkit. 2014. Available at: https://www.luthercollege.edu/public/images/Intersectionality_Tookit_and_other_resources.pdf. Accessed March 7, 2019
29. Arthritis Research Canada. Workbook to guide the development of a Patient Engagement in Research (PEIR) Plan. 2018. Available at: https://www.arthritisresearch.ca/wp-content/uploads/2018/06/PEIR-Plan-Guide.pdf. Accessed June 7, 2019.
30. Kothari A, & Graham I. Thinking deeper about integrated knowledge translation: What comes next? Presentation at: Knowledge Translation Canada Annual Scientific Meeting; May 2019; Winnipeg, MN https://ktcanada.org/wp-content/uploads/2019/05/KT-Canada_Annual-Scientific-Meeting-Program_May-2019_v7_FD.pdf. Accessed June 7, 2019.
31. George & Fay Yee Centre for Healthcare Innovation. Considerations When Building a Budget for Public and Patient Engagement. N.d. https://chimb.ca/sub-sites/1-patient-engagement?page=79-budgeting-for-engagement. Accessed June 7, 2019.
32. George & Fay Yee Centre for Healthcare Innovation. How to Engage Patients & Public in Health Research. N.d. https://chimb.ca/sub-sites/1-patient-engagement?page=75-how-to-engage. Accessed June 7, 2019.
33. Government of Canada, C. I. of H. R. Considerations when paying patient partners in research - CIHR. 2019. http://cihr-irsc.gc.ca/e/51466.html. Accessed June 7, 2019.
34. SPOR Evidence Alliance. Patient Partner Appreciation Policy and Procedure. N.d. https://sporevidencealliance.ca/about/policies-procedures/. Accessed December 29, 2019.
35. MOVES. The MOVE Program. https://www.movescanada.ca/. Accessed May 2, 2019.
36. Kuper, A., Lingard, L., & Levinson, W. Critically appraising qualitative research. BMJ. 2008;337:a1035.
37. Joanna Briggs Institute. Critical Appraisal Tools. N.d. https://joannabriggs.org/critical_appraisal_tools. Accessed March 7, 2019.
38. Critical Appraisal Skills Programme. CASP Appraisal Checklist. 2018. https://casp-uk.net/casp-tools-checklists/. Accessed March 7, 2019.
39. Government of Canada, C. I. of H. R. Equity and Diversity Questionnaire for applicants. 2018. http://www.cihr-irsc.gc.ca/e/50956.html. Accessed March 7, 2019.

Reflection Worksheet

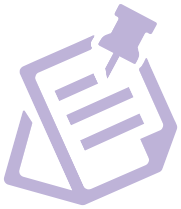

Where am I situated?

- ☐ What intersecting categories make up your identity?[1]
- ☐ Reflecting on your response to the question above, how do your intersecting categories impact your place in society?[1]
- ☐ How do your identities relate to the project's topic area? How might your place in society impact your work on this project?[1]

Who is on the implementation team?

- ☐ What does an inclusive approach mean to you?[1]
- ☐ What inclusive approaches have been used on your team, in your organization, or in other organizations? What is good or bad about these approaches? Note that not all teams or organizations take an inclusive approach.[1]
- ☐ Who is the patient, healthcare provider, and community population affected by the project topic area? What would they want to get out of the project topic area? How do you plan to get them involved?[2]
- ☐ What are the real and perceived power differences on the team?[2,3]
- ☐ Reflect on whether everyone who could be on the team has been asked if and how they would like to be involved. Think about how different perspectives that represent a range of intersecting categories have been examined.
- ☐ Does your team reflect the makeup of the patient, community, and health care providers that experiences the project topic?[2]

Identifying the Problem

- ☐ Whose point of view is reflected when defining the problem? For example, is it the Chief Executive Officer or the nurse who has prioritized a specific problem as the focus of the KT project?
- ☐ What are the information gaps in the problem area? How can these gaps be filled? Information gaps are areas where you do not have complete knowledge.

Defining the Evidence-to-Practice Gap

- ☐ Who decides which evidence-to-practice gaps is prioritized?

Selecting the Practice Change

- ☐ Of the practice changes under consideration, who is expected to change their behaviour and "do" the practice changes? This "who" could be a health professional the patient, the community, and/or another group.
- ☐ Think about the group expected to change their behaviour (e.g., nurses). What intersecting categories of group members can we reflect on? Think about the group affected by the practice change (e.g., patients). What intersecting categories of group members can we reflect on?

Appraising Evidence

- ☐ What information do I have? What information do I wish I had? Who might have this information? Who should I talk to about this?
- ☐ Critically assess the data

1. *Hankivsky O, Grace D, Hunting G, et al. ntersectionality-based policy analysis. An intersectionality-based policy analysis framework, 2012;33-45.*
2. *Arthritis Research Canada. Workbook to guide the development of a Patient Engagement in Research (PEIR) Plan. 2018.*
3. *Shimmin C., et al. Moving towards a more inclusive patient and public involvement in health research paradigm. 2017;17(1):539.*

Intersectionality & Knowledge Translation (KT)

Guide for Common Approaches to Assessing Barriers & Facilitators to Knowledge Use

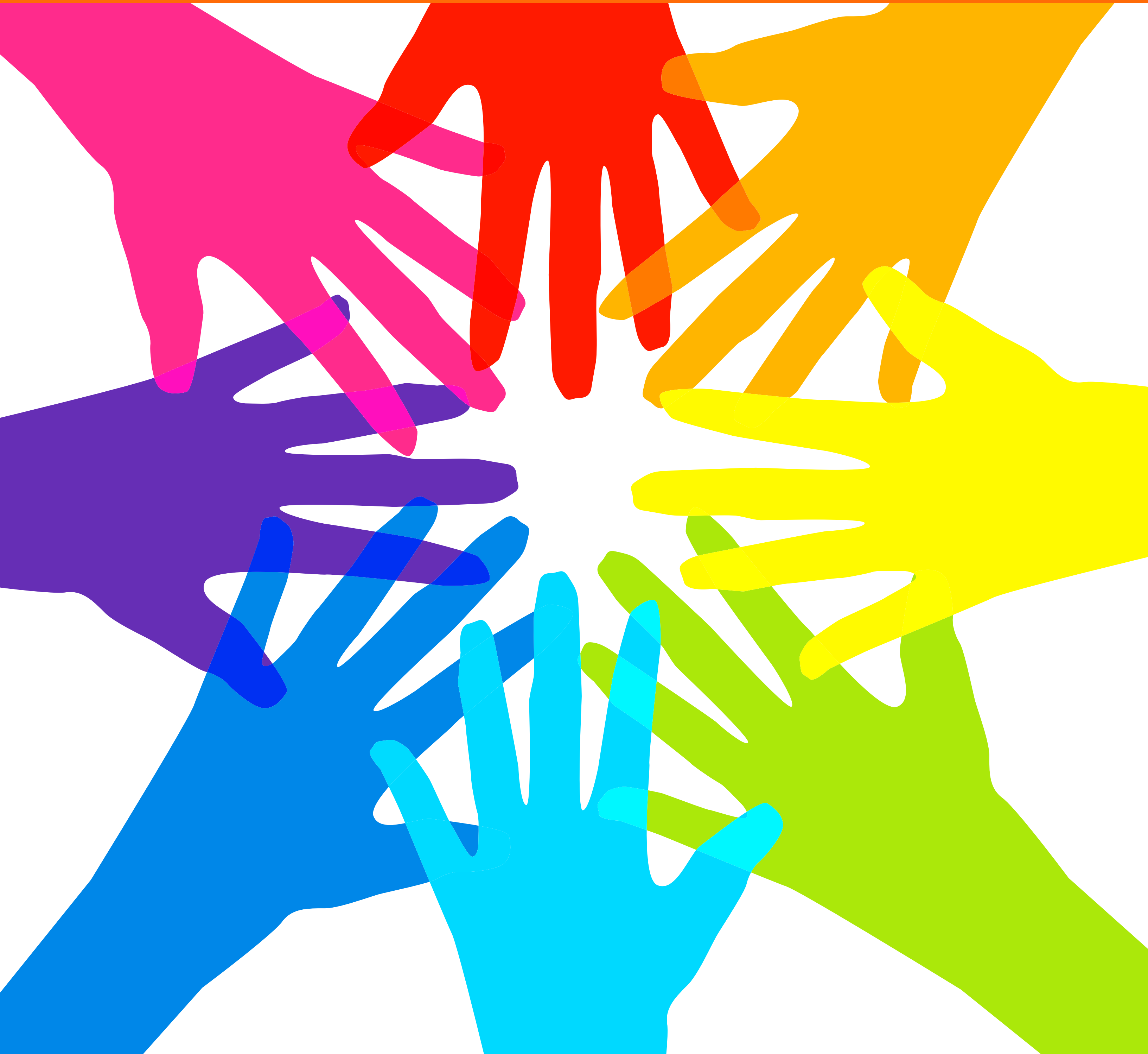

Table of Contents

**Introduction**	**3**
**Considerations for all approaches**	**6**
**Knowledge synthesis**	**7**
**Conversations with stakeholders**	**10**
**Interviews and focus groups**	**12**
**Surveys**	**17**
**Observation**	**20**
**Once the barriers and facilitators assessment is complete:**	**22**
**Appendix A: Project Limitations**	**23**
**References**	**24**

Introduction

Who is this guide for?

Knowledge translation (KT) intervention developers. KT is the process of moving evidence into health care practice.[1] KT intervention developers are people who create KT interventions designed to improve health care.

For example, a KT intervention developer may design a KT intervention to change how often nurses encourage patients to exercise in long-term care homes. The KT intervention may include restructuring nurses' workflow and delivering in-person education sessions.

Project managers responsible for conducting a barriers and facilitators assessment would find this tool particularly useful.

Why should I use this guide?

By applying an inclusive and equitable lens to KT interventions, you can design more effective interventions that address the complex realities of the people you work with.[2-5]

Intersectionality considerations are complex. This tool is meant to prompt individuals to be more thoughtful about intersectionality when conducting barriers and facilitators assessments. Please explore the included resources for more comprehensive information.

Why are certain approaches featured in this guide?

This guide covers considerations for popular approaches for assessing barriers and facilitators to knowledge use. A group of KT practitioners highlighted these approaches as ones they frequently used.

Knowledge synthesis is the first approach highlighted in this tool because KT interventions should first look at established evidence. These are not the only approaches to assessing barriers and facilitators. In practice, any mix of these approaches and others may be used.

What is the purpose of this guide?

This tool outlines prompts to consider when designing, conducting, and analyzing barriers and facilitator assessments. It relates to the "assess barriers and facilitators to knowledge use" stage of the Knowledge-to-Action (KTA) Cycle.[6]

When do I use it?

Use this tool to assess barriers and facilitators whether you are using the KTA or any other model that makes sense for your project. This tool is part of a set of tools that help us take an intersectional approach when doing KT:

- Running a KT project with an intersectional approach: Intersectionality Guide.
- An intersectional approach to problem identification, defining knowledge-to-practice gaps, and identifying practice changes to fill these gaps: Reflection Workbook.
- An intersectional approach to selecting and tailoring KT interventions using the results of a barriers and facilitators assessment: Selecting and Tailoring KT Interventions Workbook.

How do I use this guide?

Use it as a reference guide. It is meant to prompt reflection; it is not meant to be prescriptive. Everyone on the implementation team can review these considerations individually and as a team.

Who made this guide?

This tool was collaboratively developed in an iterative fashion by an interdisciplinary team of KT scholars, KT intervention developers, intersectionality scholars, and adult education experts.

Project limitations

See Appendix A for a project limitation statement.

This tool cannot be broadly applied to Indigenous Peoples, and there may be more culturally appropriate models, theories, and frameworks that are useful to consider when conducting projects that involve Indigenous communities.

Key terms

Please note that the key terms discussed on this page provide only a quick overview of intersectionality and KT. For more information, refer to the Intersectionality Guide, which outlines the following:

- Resources to reflect on power and team dynamics
- Intersectionality and intersecting categories
- Knowledge translation and the Knowledge-to-Action (KTA) Cycle

Intersectionality* is a way of looking at the world that recognizes that people's experiences are shaped by a combination of social factors, including their gender, racialization, age, among others.[7-13] These experiences occur within and interact with a context of connected systems and structures of power, such as sexism and racism.[7-13]

Note that there are various definitions of intersectionality and that they are evolving.

Intersecting categories include age, gender identity, sex, and other aspects of one's lived experience. These aspects interact to form a person's identity (See Figure 1).[3,12,13] One's intersecting categories reflect larger systems of oppression/privilege (e.g., sexism, ageism)[3,12,13]

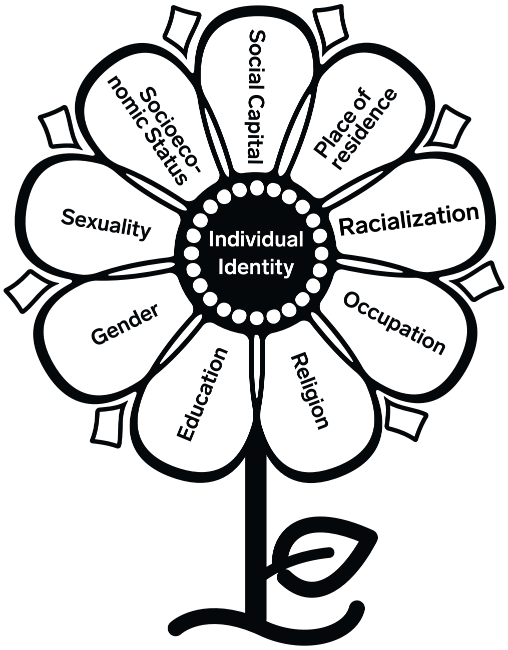

Figure 1. Visual representation of some intersecting categories.[12,14,15] The categories mentioned in this figure are not an exhaustive list.

Knowledge translation is the process of getting evidence used in practice.[1]

Barriers and facilitators are factors that impede or enable knowledge uptake, respectively.

Evidence is broadly defined in this tool as information that originates from research and is derived through scientific evaluation. As a group, you may define evidence differently. As an implementation team, reflect on the following:

- ☐ What your team defines as evidence
- ☐ What your team defines as high-quality evidence
- ☐ What your target audience defines as credible evidence

How do I take an intersectional approach to KT?

Intervention developers can take an intersectional approach to KT by considering the dynamic nature of social identities and their interactions with social structures and systems that may oppress or privilege different groups.

These social identities and their interactions can be considered at all stages of the KT process. When doing this, you can think about the people designing (e.g., KT intervention developers) and receiving (e.g., clinicians) the KT intervention and those affected by it (e.g., people with lived experiences).

By taking an intersectional approach in your work, you can identify the root causes of inequities, overcome conceptual gaps, and consider complex factors together to create an effective KT intervention.[2-5]

General resources

Consult the resources below for more information on key topics.

For more on building a business case for a barriers and facilitators assessment as part of a full KT project, visit **RNAO (2012) Toolkit:** https://tinyurl.com/yyu5zq7z[16]

Budgeting resources

Determine what resources are available in the project budget for assessing barriers and facilitators to knowledge use. Aim to execute what is reasonable for your budget.

Key resource-related factors to consider[17]:

- ☐ People
- ☐ Money
- ☐ Time
- ☐ Equipment, supplies, and technology
- ☐ Space
- ☐ Supports for taking an inclusive approach
  - (e.g., translators, caregiving, and meals)

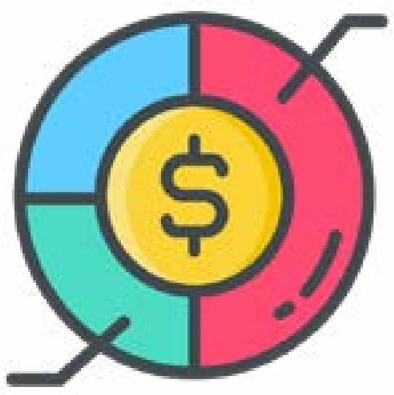

Team engagement takes time and resources. To help estimate a budget for team engagement, consult this free downloadable budget tool: https://tinyurl.com/y2x78oww[17]

For a free, downloadable tool for budget estimates for interviews and focus groups, visit https://tinyurl.com/y5hch7ye[18] You can expand on this budget tool when using other approaches.[18]

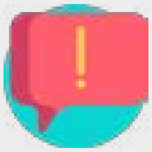

Please note: Taking an intersectional approach is needed to recognize the importance of individuals' social identities within the greater context of systems and structures of power which reflect macro systems of privilege and oppression. Keep in mind that recognizing areas of advantage, disadvantage, and oppression may bring up feelings of confusion, guilt, distress, among others. It is okay to feel uncomfortable. There is a difference between feeling uncomfortable and unsafe.

Considerations for all approaches

This guide covers considerations for popular approaches for assessing barriers and facilitators to knowledge use, including: knowledge synthesis; conversations with stakeholders; interviews and focus groups; surveys; and observations.

Why are all these common considerations important?

When undertaking any assessment of barriers and facilitators to knowledge use, it is important to reflect on who we are, who is on our team, and the context of our assessment. By taking time to reflect on these elements, we can be more aware of strengths and limitations of our assessment.

In taking time to reflect, you can design more effective interventions that work to address the complex realities of the people you work with.[2-5]

When using any approaches found in this tool, be sure to dedicate time for team members and project participants to tell their stories. Use the following tips to do this:

- ☐ Provide open space and support for story sharing.
- ☐ Reach out to the group in advance to identify the supports they need to share their story.
- ☐ Do not cut people off while they are sharing their story. This can be traumatizing.
- ☐ Provide a list of low-cost supports in the community.

Have a team member do some training on taking a trauma-informed approach. For more information on such approaches, visit:

- **Shimmin et al. - Moving towards a more inclusive patient and public involvement in health research paradigm:** https://tinyurl.com/yb5sh3ed[19]
- **Alberta Health Services - Trauma Informed Care:** https://tinyurl.com/v4dbzyx[20]

All of the considerations outlined in this tool are important to think about.[19] Taking an intersectional approach does not mean you can account for every consideration.[19] Balance these considerations with budget, time, and other resource constraints. However, there are aspects you can always reflect on:

- ☐ Who is on your team?
- ☐ What experiences and perspectives do you bring?
- ☐ How can you involve individuals with lived experiences on the topic?
- ☐ Who has power on your team?
- ☐ How can you ensure that everyone on the team has an opportunity to share?

When planning, you should always work to enable participation from individuals from a range of intersecting categories. You can use a model, theory, or framework to guide a barriers and facilitators assessment. For example, you can use the Intersectionality-Enhanced Consolidated Framework for Implementation Research (CFIR).[21]

When discussing consent with participants, outline risks of data breaches and how these risks are mitigated. In preparing for this discussion:

- ☐ Reflect on power dynamics.
- ☐ How would survey participants feel if someone in power was able to identify their responses?
- ☐ What risks exist from their perspective?
- ☐ Consider how international data storage may impact privacy risks.
    - For example, data stored in the US may be accessed through the US Patriot Act.[22]

When collecting information to assess barriers and facilitators, you should ensure that the implementation team is committed to respecting the privacy and confidentiality of the information. As with any KT project, you should refrain from sharing any identifying information without participant consent.

Knowledge synthesis

Knowledge synthesis:

A comprehensive assessment of evidence related to barriers and facilitators to behaviour change in a particular topic area. The purpose is to uncover major barriers and facilitators across multiple studies or projects.[23,24]

Please note that this tool discusses how to search for knowledge syntheses. For more information on how to conduct knowledge syntheses, please consult the resources below.

To learn more about general recommendations on how to conduct systematic reviews or knowledge syntheses, visit: **Tricco, A.C., et al. The art and science of knowledge synthesis:** https://tinyurl.com/yyvx6x8r[25]

For more information on types of knowledge synthesis methods, visit **Knowledge Translation Program JCE Series - Knowledge Synthesis Methods:** https://tinyurl.com/y6nstgjw[26]

For guidance on identifying the type of review best suited for a project needing knowledge synthesis, visit **Knowledge Translation Program - What Review is Right for You?:** https://tinyurl.com/y2mvenwd[27]

You should use a reporting guideline to accurately and transparently report your work. Here are examples of relevant reporting guidelines:

- **Welch et al. - PRISMA-Equity Extension: Reporting Guidelines for Systematic Reviews with a Focus on Health Equity:** https://tinyurl.com/yxwh629n[28]
- **The Campbell and Cochrane Equity Methods Group - Equity Checklist for Systematic Review Authors:** https://tinyurl.com/y3lmyk5h[29]

Considerations for designing

Who is designing the search for knowledge syntheses?

You should involve those with lived experience with the topic area. They can help identify relevant areas of inquiry and terms.

If available, you can engage information scientists (e.g., a librarian) who have extensive knowledge on best practices for literature searches.[30]

Specify the topic. Define a research question.

To avoid having too much material returned in your searches, you can use the following prompts to develop your research question[31,32]:

- ☐ Who is changing their behaviour?
  - Investigate whether the reviews you find excluded certain populations. If so, why? Consider exploring the original studies contained within a review to understand who may have been excluded.
- ☐ What is the desired behaviour change (e.g., the “clinical intervention” or the “practice change”)?
- ☐ What is the current behaviour?
- ☐ What are the barriers and facilitators to behaviour change (e.g., memory, fear, lack of skill)?
- ☐ How were barriers and facilitators to behaviour change assessed?

What are you searching for?

Again, reflect with the team on the following:

- ☐ What your team defines as evidence
- ☐ What your team defines as high-quality evidence
- ☐ What your target audience defines as credible evidence

Consider the inclusion/exclusion criteria of the knowledge syntheses:

- ☐ **Timeline of sources** (i.e., how far back do you want to review? Are there outdated stereotypes or perceptions in the sources to be mindful of?)[33]
- ☐ **Types of sources:** articles, books, reports, letters to the editor, newspaper articles, diary entries, etc.[33]
- ☐ **Types of phrases and keywords:** ask those with a range of experiences for search terms and relevant phrasing for barriers and facilitators. Consider how other health care systems (e.g., US, UK) may phrase barriers and facilitators information.

Once you have developed your search strategy, check to see if preliminary results match your original question.

Where to search?

Investigate the following sites to check for reviews that are published or in progress. You can critically appraise reviews using an intersectional approach. Please see the Reflection Workbook for more information on critical appraisal.

- **Joanna Briggs Institute:** https://tinyurl.com/yxcsyad5[34]
- **Trip:** https://tinyurl.com/htbetre[35]
- **Cochrane Library:** https://tinyurl.com/y3cbw5br2[36]
- **PROSPERO International:** https://tinyurl.com/y52kpaof[37]
- **PubMed:** https://tinyurl.com/472npj[38]
- **Implementation Science (journal):** https://tinyurl.com/yy4u2rfz[39]

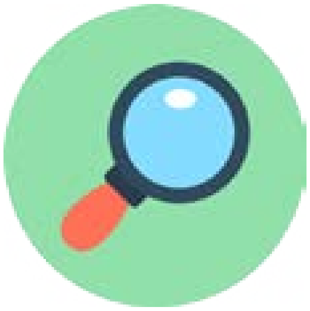

Share the load, share perspectives and approaches

Include team members with a range of experiences and perspectives when reviewing and appraising the literature.

- A quality assessment and risk of bias assessment should be done independently by at least two individuals. This may require additional training.

As a team, discuss themes that arise related to barriers and facilitators assessments. How might these themes interact with barriers and facilitators in your project?[16] For example, are there themes of childcare and gender roles present across the syntheses?

Work with those with lived experience in the area to understand these themes.

Reflect on search results

As a team, reflect on these considerations while reviewing the literature:

- ☐ What does your team consider to be “expertise” on a topic?
- ☐ Consider many forms of expertise, including academic credentials and lived experience.
- ☐ Is the author of a source an expert in their field?
- ☐ Consider the power dynamics of those who wrote the publication (e.g., if looking at a government document, consider the biases and motivations that could have impacted the conclusions).
- ☐ Were the original studies exclusionary? Were the perspectives of certain groups of people excluded from participating?
- ☐ When was the investigation conducted and published (e.g., are you looking for literature where data was collected prior to a major legislation change)?

It is key to evaluate the quality and risk of bias of each piece of evidence.[23,32,40] A quality assessment and risk of bias assessment should be done independently by at least two individuals.

- ☐ This may require additional training
- ☐ Assess the quality of a completed knowledge synthesis.[25] Look at the Reflection Workbook to learn how to enhance existing appraisal tools with an intersectional approach.
- ☐ Ask those with lived experience on the topic whether the results of the synthesis resonate with them. Are there elements of the review (e.g., rural location) that align or do not align with your project?

What to do when no information is discovered

There are situations when the information you are looking for is not available. If this happens, look beyond peer-reviewed research and consider the following sources:

- ☐ Content from industry leaders
- ☐ Internal checklists
- ☐ Different types of evidence (e.g., lived experience commentaries)
    - Reflect on whether there are organizations you work with that have looked into a similar intervention? Did they report any results of a barriers and facilitators assessment?

It is common to not discover any information during this process. This is okay. You can use other methods to assess barriers and facilitators.

Examples of quality and risk of bias appraisal tools (without intersectionality enhancements):

- **AMSTAR team – Assessing the Metholodological Quality of Systematic Reviews (AMSTAR 2):** https://tinyurl.com/y2qzv86a[41]
- **Joanna Briggs Institute – Critical appraisal tools:** https://tinyurl.com/y32xg8ln[42]
- **Critical Appraisal Skills Programme – CASP Appraisal Checklists:** https://tinyurl.com/y7qx99mq[43]
- **Bridget O'Brien – Standards for reporting qualitative research:** https://tinyurl.com/y4av7whx[44]

Considerations for analyzing data and reporting

Include all relevant components of the story

Share the results of the search and the team's process to conduct and appraise the results. You can also report intersectionality considerations related to your team:

- What biases may team members and the team as a whole hold? How did your team try to mitigate these biases?
    - – What perspectives were missing from the team?

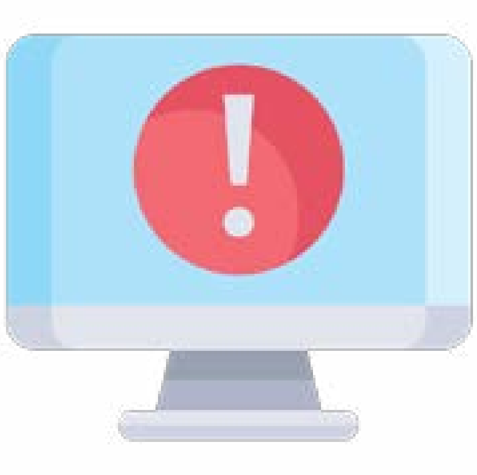

Conversations with stakeholders

Collect information from informal discussions with participants on barriers and facilitators to knowledge use. These casual conversations typically occur in an unstructured setting (e.g., before a project meeting begins).[45]

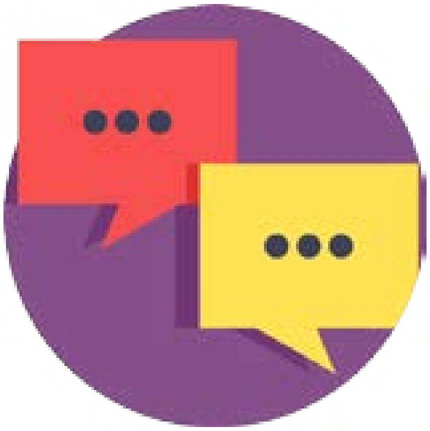

General resources

For more on informal conversations

with stakeholders, visit **RWJF - Informal Interviews:** http://tinyurl.com/yxnfrcmm[46]

For more on how to structure open-ended questions in conversations with stakeholders, visit **Changing Minds - Open and Closed Questions:** http://tinyurl.com/nvfq8n[47]

For key tips on active listening, visit **Garzon, J. - Key Tips for Active Listening:** http://tinyurl.com/y5ywhmcw[48]

Considerations for designing/set-up

Mapping stakeholders

Before you talk to stakeholders, you need to reflect on your own biases. Consider the following as a team:

- ☐ Who is affected by the project?
- ☑ How does your team define who is a stakeholder?
- ☐ Who are the key individual stakeholders at different levels of the organization?
- ☐ Who is managing the relationship with stakeholders?
- ☐ Would anyone on the team with lived experience with the topic like to share their perspective?
- ☐ Why are you planning to have conversations with specific individuals?
- ☐ Who are you not speaking with? Why not?

Map all the stakeholders involved, their types of expertise, and the conversations you would want to have.

Exploring consent

Because these conversations arise naturally, the individual or group you are speaking with is not explicitly consenting to sharing information for the purposes of a barriers and facilitators assessment.

If key information is shared, ask your conversation partner if you can use the information in a de-identified way in a barriers and facilitators analysis. If the information is sensitive and there are repercussions for the person sharing it, carefully reflect on whether to use this information. In particular, consider the following:

- ☐ Do not assume consent.
- ☐ Do not use any recording devices because they breach the sense of trust in the conversation.[50]
- ☐ Do not use an interview guide or any materials during the conversation.[51]
- ☐ Do perform conversations "on the fly" so that respondents see it as just a natural conversation.
- ☐ Do find opportunities to communicate frequently in an informal setting with key stakeholders.[49]
- ☐ Do follow organizational guidelines on consent even if you are not in a formal research setting.

Considerations for conducting conversations

Who are you speaking with?

Reflect again after conversations have begun. Consider who you are speaking with:

- ☐ Are conversation partners actively participating in conversations?
- ☐ Are you speaking with enough people to capture a range of perspectives?

Build relationships and ensure safety (emotional, psychological, and spiritual) to those you are speaking with:

- ☐ Have a conversation on safety and power with partners with an open mind
- ☐ Highlight that they can share only what they are comfortable with
- ☐ Provide context on intersectionality (e.g., recognize your intersecting categories in the conversation)
- ☐ Emphasize trust and safety before exploring anything else
- ☐ Ask participants what consent means to them

For information on building trust and partnerships, visit **Jagosh, J. - A realist evaluation of community-based participatory research:** https://tinyurl.com/y627k6m6[49]

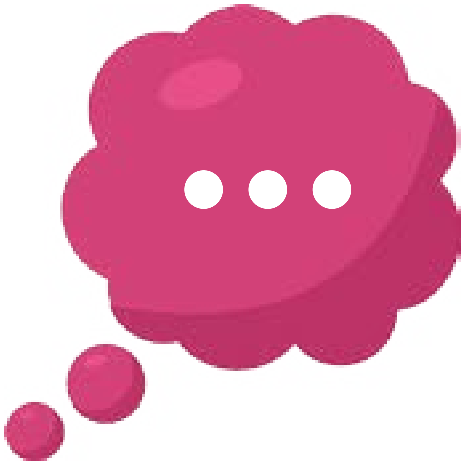

Conducting the conversation

- ☐ Focus on key barriers and facilitators identified by the stakeholders (i.e., choose quality of barriers and facilitators over quantity)
    - If the conversation deviates from a key barrier or facilitator, refocus the conversation.[52]
- ☐ Talk with the participant to understand their way of understanding the barriers and facilitators[51]:
    - See how they react to certain topics, issues, and themes.[53]
    - Ask them to contextualize their barrier/facilitator based on their social histories. Ask if there is important historical knowledge you need to understand the barrier/facilitator.[54]
- ☐ Discuss risk or safety concerns by outlining which conversations are private and your duties to report.
- ☐ Use active listening techniques and ask follow-up questions.
- ☐ Provide your contact information to the conversation partners so they can follow up with any thoughts. Highlight that you are happy to speak by phone, email, or another medium.

Record rough notes immediately after the conversation.[46]

- ☐ Do not use names or identifiers when recording notes
- ☐ Aim to record all notes within 24 hours of the interaction
- ☐ Consider how your perspectives, biases, and experiences may impact what you record

Data storage

Although the informal conversation may not constitute formal data collection, store notes in a safe and secure location.

Interviews and focus groups

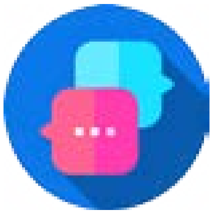

Interview

A one-to-one conversation between a trained interviewer and a participant about the participant's experience with barriers and facilitators to knowledge use. Interviews may be semi-structured such that the interviewer's questions are predetermined. However, follow-up questions can follow the natural flow of the conversation.[55]

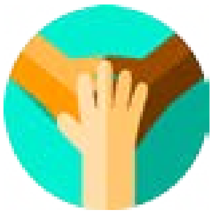

Focus group

A small group discussion run by a trained facilitator that explores barriers and facilitators to knowledge use, typically in response to open-ended questions from the facilitator.[56] The group size is typically 8–10 participants.[56] Focus groups often include a note taker, who records the content of the discussion.[56]

Learn more about conducting qualitative research:

For a more in-depth discussion on conducting qualitative research, visit **Medecins Sans Frontieres - A Guide to Using Qualitative Research Methodology:** https://tinyurl.com/zevdv93[57]

For additional information on intersectionality-informed analysis, visit **The Institute for Intersectionality Research & Policy, SFU - Intersectionality-Informed Qualitative Research:** https://tinyurl.com/y2u5o4kk[58]

For an example of how to incorporate intersectionality into interviews, visit: http://tinyurl.com/y4486cpu[59]

For more information on intersectional approaches to interviews and focus groups, visit http://tinyurl.com/y4llu6jk[60]

For more on conducting a focus group, visit http://tinyurl.com/gmmnjye[61]

Considerations for designing/set-up

Training

☐ If appropriate for your project, consider having interviewers with similar intersecting categories to those populations you are assessing

- E.g., if your study is concerned with a particular language group or practice area
- This may not be appropriate for every project. You should reflect on how biases may affect this approach.

☐ Ask the interviewer and note taker to reflect on their intersecting categories and the complexity of their identity within the research context (explore resources in the Intersectionality Guide).

- Reflect on power dynamics between interviewers and participants (e.g., if the interviewer is a cis white male and participants are trans women of colour, what power dynamics might exist?)
- Reflect on power dynamics between participants (e.g., if staff members and management are included in the same focus group, will staff feel comfortable voicing barriers related to management?)

☐ Investigate and execute processes to work appropriately with marginalized groups (e.g., educational resources, historical context, protocols, interpersonal interactions).

☐ Ensure that the facilitation process will respect everyone's strengths and contributions.

☐ Craft open-ended questions that do not contain assumptions or binaries (e.g., instead of asking "how might being a man or woman influence how this task is done," ask "how might someone's identity influence how the task is done?").[54]

Practice

Run a practice interview with members of the implementation team. In other words, practice with colleagues who are helping to design the intervention before conducting the interviews with your target audience.

The trained interviewer and note taker can complete a mock interview with team members. Team members can provide feedback to the interviewer on format, flow, effectiveness, and inclusivity for participants with a range of intersecting categories.

Less experienced interviewers can sit in as note takers until they are ready to begin interviewing/facilitating themselves.

Practice responding to frequently asked questions in multiple ways. Remember that participants will have a range of intersecting categories and communication styles.

Recruiting

- Explore in-person and online recruiting strategies (e.g., recruiting at a staff education day, using a mailing list).
- See recruiting resources in the Intersectionality Guide.
- Tailor recruitment materials to a range of audiences. Make efforts to include marginalized groups.
- Use samples sizes that are large enough to capture multiple intersecting categories.[43]
- It is not feasible to speak to everyone. However, you should make a particular effort to speak with those you typically do not hear from.
- You can budget for large enough sample sizes up front when planning projects.
- When considering compensation, reflect on how compensation can influence who responds to recruitment materials
- Outline compensation terms and methods up front (e.g., immediately after the interview, a cheque will be available for pickup at this location).

Preparing participants

- If appropriate, consider sending background material and the interview questions to participants before the session (i.e., include plain language definitions of key terms related to your project).
- Offer to convey this information in multiple formats (e.g., mail, phone, email, in-person) tailored to the needs of the participant.
- Inquire about the participant's preferred choice of communication (e.g., in-person, phone, or video).
- Reflect that everyone has different experience with technology. Offer participants the chance to test any technology before the interview.

To learn more about participant compensation, visit **Canadian Institutes of Health Research - Considerations when paying patient partners in research:** https://tinyurl.com/y6jvlta5[62]

Preparation

- Provide a consent form to the participant in advance. In addition, provide a printed version during an in-person interview or focus group. Offer multiple opportunities and venues for answering participant questions.
- Plan for and budget to accommodate needs and preferences (e.g., microphones, religious or cultural days, childcare, support companion).
- Select a fully accessible venue if conducting an in-person interview or focus group.
- Arrive at the interview or focus group location at least 15 minutes before the start time. Ensure audio/video equipment is working.
- Have plain-language background information about the study and a script ready for the facilitator and note taker.
- Highlight important questions or probes to prioritize in case of time constraints. Ask those with lived experiences to help prioritize key questions.

How can you prepare interview/ focus group materials?

National Implementation Research Network - Interview tips (see pages 5 – 13)

https://tinyurl.com/y266ypej[63]

National Implementation Research Network Interview Video Examples:

https://tinyurl.com/y43n494q[64]

For more on patient engagement, visit **Arthritis Research Canada's Workbook to guide the development of a Patient Engagement In Research (PEIR) Plan:**

https://tinyurl.com/yyrugmc5[65]

Access Alliance - Everyone can do research (see pages 51 – 54 for interview related tips):

https://tinyurl.com/accessalliance[66]

Considerations for conducting interviews and focus groups

General interview/focus group procedures

The following are common principles for conducting interviews/focus groups:

- [ ] When possible, ensure that each interview includes a facilitator and the note taker.
    - At the beginning of the interview/focus group, you should introduce yourself and state your pronouns.
- [ ] Review the purpose of the interview/focus group and the terms of consent with each participant. Be prepared to answer any questions they may have.
    - Individuals may have different questions and responses about consent, especially given historical relationships between researchers and certain groups. Your team should be sensitive to these histories and possible questions.
- [ ] Create a space where the interviewee feels they can stop the discussion if necessary or ask clarification questions if they require more information.
- [ ] For focus groups, consider using an approach where each participant has a chance to answer the question presented before group discussion begins. This is sometimes called a round robin approach
- [ ] When following up on a line of inquiry, continue to ask open-ended questions, such as "can you explain further?" or "can you give me an example?"
- [ ] Monitor the time to determine the pace of the interview/focus group.
- [ ] At the end of the interview/focus group, thank the participants for their time and, if applicable, provide them with information on follow-up support services (e.g. help lines).
- [ ] Where possible, provide the opportunity for participants to follow up with any stories or reflections that they did not get to share in the interview/focus group.
- [ ] Process participants' reimbursement and compensation in a timely manner.
- [ ] Follow up with participants about the project results.

Making sense of interviews and focus groups

There are many ways to go about interpreting the information shared in interviews and focus groups.[67] In general, analyses are iterative, follow a systematic process, and involve discussion with the groups you have interviewed.[67]

Coding

Coding is a process of organizing themes and concepts that emerge across interviews or focus groups.[68] You and your team will likely code word-for-word transcripts or detailed notes of interviews and focus groups.

For barriers and facilitators assessments, you will generally follow one of or a mix of two overarching approaches:

1. Establish themes first and then code transcripts with themes:
   - This approach may be preferred for areas where barriers and facilitators to behaviour change are well known from existing knowledge.[67]
   - Working with a group that represents a range of intersecting categories, discuss themes that you anticipate will arise in transcripts.
   - Consider further refining codes to reflect specific aspects of intersectional experiences (e.g., intersection of gender roles and age).[69]
2. Review transcripts first and then note themes that emerge:
   - This approach may be preferred for areas where barriers and facilitators are not well known.
   - At least two coders that represent a range of intersecting categories will review transcripts in a systematic way to identify themes. You can use Table 2 of Bradley et al.'s work to define code types and purposes.[67]
   - Reflect on and code for information in transcripts that reflects intersectional themes (e.g., power dynamics between nurses and doctors).

Adapting these approaches together is sometimes called multistage analysis or the Framework Approach.[67, 69, 70]

When using any coding approach, you and those on your team should consider:

- Before coding, first reflect on your own biases and experiences and how these might influence the coding process.
- Acknowledge and plan to manage power differences that may exist between coders.
- If one coder manages the other, set up discussion guidelines to ensure all voices are heard.
- When coding, consider how participants' identities are experienced and how their experiences were influenced by social and historical contexts.
- Write down notes throughout the review process to explain your reasoning and themes you are unsure about. Compare annotations with fellow coders.
- Two independent coders should review and code at least 20% of the full transcripts. For example, two people independently review two out of ten interview transcripts.
  - Hold consensus meetings to discuss and reconcile discrepancies between the two coders.
- Here are some examples of software programs you can use to code

  NVivo: https://tinyurl.com/y5alm69x[71]

  Raven's Eye: https://tinyurl.com/y5mkxlkh[72]

  Dedoose: https://tinyurl.com/yyg3lmdt[73]

  MAXQDA: https://tinyurl.com/y6x29o4s[74]

Considerations for analyzing data and reporting

Review results in context

Ask a sub-set of interview participants and/or other people with lived experience to review the results.

- Explore whether they think the themes uncovered in the interviews are reasonable.
- Discuss what language could be used to contextualize the results.

- Refer to the Reflection Workbook's section on self-reflection
- This process will likely require research ethics board approval. Plan for this in budgets and timelines.
- Consider who is included/excluded in the results to identify gaps.

Consider the power dynamics among interviewers, focus groups facilitators, and participants. For focus groups, also reflect on power dynamics amongst participants.

- Did participants feel comfortable sharing their thoughts with the facilitators?
- Are there power dynamics among participants to consider?

Report full results

You should report results accurately and transparently using the COnsolidated criteria for REporting Qualitative research (COREQ) checklist.[75]

- You can also report additional intersecting categories that are not listed in the COREQ checklist.

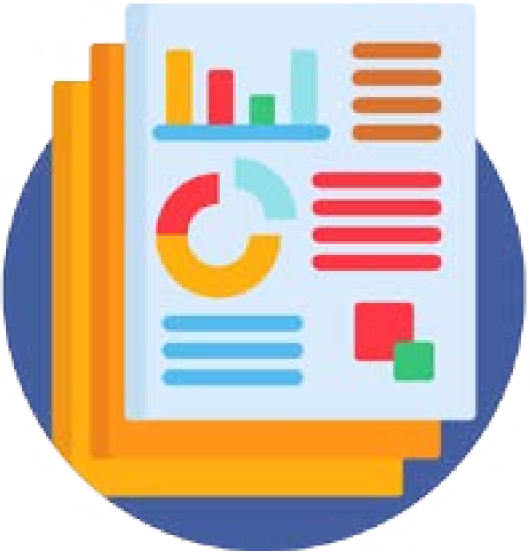

Surveys

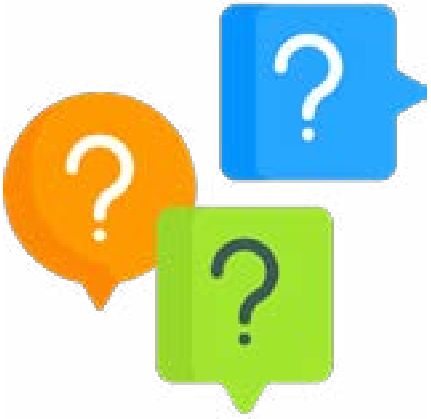

Survey

A method of gathering information on barriers and facilitators from individuals or groups through their responses to structured questions.[76-79]

Surveys can be conducted in multiple forms, such as face-to-face, by telephone, in writing, and online.

General resources

For example questions on intersectional concepts, including cultural diversity, visit **Columbia College - Cultural Diversity Self-Assessment survey:** http://tinyurl.com/y5tolxy9[79]

For suggestions on crafting surveys, see **Dillman's Principles for Questionnaire Construction:** http://tinyurl.com/yyylkdq7[80]

For more in-depth information on taking an intersectional approach in quantitative analysis:

- **Rouhani, S. - Intersectionality-informed Quantitative Research: A Primer:** http://tinyurl.com/y25qp87g[81]
- **Bauer, G.R. & Scheim, A.I. - Methods for analytic intercategorical intersectionality in quantitative research: Discrimination as a mediator of health inequalities:** http://tinyurl.com/y5w5suc9[82]

Considerations for designing/set-up

Development

Always refer to project objectives when designing surveys[83]:

- ☐ If a question is not related to project objectives, does it need to be included?
- ☐ Be respectful of participants' time and efforts by excluding unnecessary questions.
- ☐ Craft questions that are free of stereotypes and binaries.[54]
- ☐ Consider separating intersecting categories in demographic sections. Prompt respondents to "check all that apply" for identity factors that influence their experience.
- ☐ Give the option of selecting multiple answers (e.g., ethnicity, sex, gender identity, sexual orientation).
- ☐ Provide "N/A" and "I prefer not to answer" so participants are not forced to provide an answer.
- ☐ Avoid the term "other." Consider including a text box or blank line for participants to input their own response. For face-to-face or telephone surveys, create space for participants to provide their own responses. For example, you can ask "is there anything you would like to share that we have not discussed.
- ☐ Avoid questions that totalize people's experiences, such as agree/disagree questions.

Overall, reframe gender identity and other intersecting categories as "structural categories and social processes rather than primarily the characteristics of individuals."[84,85] This may lead you to explore how factors beyond an individual's control may combine with other variables at organizational and system levels.[84,85]

For more, visit **Status of Women Canada's Gender-Based Analysis Plus Research Guide:** https://tinyurl.com/y4bzqqgx[85]

Differentiate between ethnicity, culture, and race when

collecting data:

- ☐ Consider different ways ethnicity and race are categorized.
- ☐ Reflect on how you are planning to use this data and consider the pros and cons of collecting this information.

Collaborate with those with lived experiences for phrasing to use in surveys. Use terms that physiotherapists use in their practice if they are the survey's target audience.

Use questions that have been shown to be valid, particularly for the intersecting categories relevant to the project.[82]

Let participants complete the survey multiple sittings. Limit the survey length and the number of questions presented on each page.[83]

Consider how to make the survey accessible for everyone.

- ☐ Could you change the font size and colours so that those with visual impairments can more easily participate?
- ☐ Could you provide an audio version of the survey?
- ☐ Could you translate the survey into multiple languages?
- ☐ Is the survey in plain language?
- ☐ Is the survey compatible with screen readers for people with visual impairments?

Provide the contact information for one team member who can answer questions participants may have.[86]

Review the questions and consider how a participant may respond. Would they be able to easily record a response?[87]

- ☐ Consider sending reflection prompts before distributing the survey to give respondents time to contemplate their responses.

Reflect on your team's assumptions about where, when, how, and why someone will fill out the survey:

- ☐ Are they at work?
- ☐ Are they answering the survey out during work hours?
- ☐ Are they answering it online?
- ☐ Why are they filling out the survey?

Conduct usability testing on the survey before administering it.

- ☐ Ask colleagues or project partners with different backgrounds, experiences, and communication preferences to test the format, flow, and effectiveness of the survey.
- ☐ Consider the accessibility of the survey if it is in an online version

If you are using an online survey, are you assuming that participants are technologically literate and able to navigate to and through the survey?

- ☐ Are you assuming that participants have access to computers and smartphones?
  - If someone does not have or is not able to use a particular device, how can their voice be incorporated?
- ☐ Is the text large enough to be reasonably read by those with a range of vision abilities?
- ☐ How would people with a range of hearing abilities complete a telephone survey?

Consider different modes of sharing/completing a survey (e.g., in person, online, by mail, via social media, over the phone).[86,88] Advertise to potential participants that the survey can be completed in multiple modes based on their needs and preferences.

Where relevant, be sure to be compliant with your research ethics board requirements:

- ☐ E.g., consider how reimbursement is handled with deidentified responses.
  - Consider questions beyond privacy (e.g., risk for traumatization).

Considerations for conducting surveys

Survey administration

To maximize response rates, send reminder messages at 1-, 3-, and 7-week intervals after initial contact.[89]

- Use different means of follow-up communication that are tailored to participant needs.
- Are there groups who are not responding to the survey? Why not? How can you enable everyone's participation?

You can use these online resources for survey administration (free to use, web-based):

SurveyMonkey: https://tinyurl.com/a7u4ar[90]

Qualtrics: https://tinyurl.com/bqjvz5f[91]

REDCap: https://tinyurl.com/y294q54j[92]

Google Forms: https://tinyurl.com/lfqd9hl[93]

Data collection

Ensure that all data is protected and cannot be accessed by anyone other than designated team members.[76,83] Where appropriate, keep data organized by individual record or response so it is easy to facilitate comparisons between people from different groups across multiple intersecting categories.

Considerations for analyzing data

Data analysis

If demographic information is collected, consider barriers and facilitators experienced by those who have particular intersecting categories (e.g., gender identity, geographic location, and ethnicity).[54]

- Avoid large group categorizations that may miss intragroup differences.[94]
- Balance the need to report disaggregated results with the need to ensure participant anonymity.
- ☐ For example, do not report results with fewer than five individuals in a cell or grouping.

Plan for how to categorize/analyze data collected from open-ended questions.

If data was collected at multiple time points, consider the following:

- At what time point are you planning to analyze the data (e.g., after all data has been collected or at intervals)?
- What time points are relevant to those with lived experience?

☐ For more information on data analysis, visit http://tinyurl.com/y5w5suc9[82]

Reflect on modifications

If any changes were made to the survey after it was deployed, why did the changes happen?[95]

- E.g., did you change the size of a response box to allow participants to provide more background context?

How can you use these lessons learned in future surveys and projects?

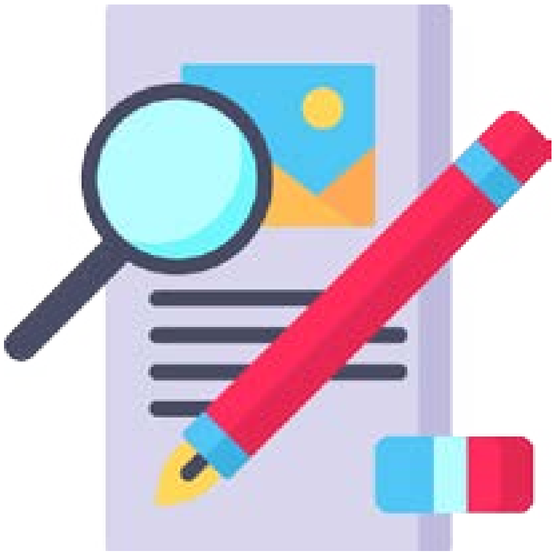

Observation

Observation

A method in which a trained observer collects information in an environment to better understand barriers and facilitators (e.g., barriers and facilitators on communication patterns and adherence to policies).[96] The observer typically uses a template to record field notes in a structured way.

Think outside the box

Consider alternative ways to conduct observations. When doing this, keep data security and participant privacy needs in mind.

For example, the Photovoice process empowers participants (usually with limited power due to language, culture, age, or other intersecting categories) to observe, identify, and discuss their barriers and facilitators through a specific photographic technique.[97] Note that because participants and personal identifiers may be photographed, participant data may not be protected through this method.

- Follow research ethics board principles on privacy, consent, and protection for participants.

For more information on assessing community needs and resources, visit https://tinyurl.com/y56lt3yg[98]

For an example of using Photovoice to understand cardiovascular health awareness in Asian elders, visit https://tinyurl.com/yyryq5rz[99]

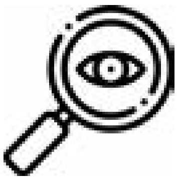

General resource

For more on participant observation, visit **Research Methods and Statistics 2.2. Participant Observation:**

http://tinyurl.com/yyx7ev5v [96]

Considerations for designing/set-up

Consider expectations

When participants are not aware that they are being studied, they must remain anonymous and the behaviour must occur in a public setting where people would not typically have an expectation of privacy.

Observe inwards

Have trained observers reflect on what they bring to the observation. What experiences have they had that may influence what they choose to record or not record?

- Use the Reflection Worksheet to guide self-reflection.
- When categorizing peoples' gender, race, or ethnicity, acknowledge the team's biases and the subjective nature of this process.

Setting up the observation template

- Leave blank spaces to record the time and date of the observation.[100]
- Use wide margins; record rough notes and thoughts here.[100]
- Include room for diagrams/maps of the physical setting (this can help us think through barriers and facilitators).[100]

Training

An observer in training can complete a mock observation with a more experienced rater (sometimes called a "gold standard rater") on the team. Trainees can complete observations individually, compare among one another, and determine if the notes were 10–20% in alignment.

- Use an assessment sheet when comparing results with others. This will help establish a baseline.

Considerations for conducting observations

Addressing biases

Participants may knowingly or unknowingly alter their behaviour as a result of being observed.[46] These are ways to mitigate this effect[101]:

- Make multiple visits.
- Consider having someone who works in the setting serve as the trained observer.
- Conduct observations in the least intrusive space in the setting so participants' typical behaviour is not disturbed.
- Do not share what you think may be happening (i.e., the barriers and facilitators) with those being observed.

Further, it is important that you acknowledge the biases you bring to this research so that you can recognize how they might influence the work you do.

During the observation period

- Do not speak with other members of the study team (e.g., another observer) about observations until all observations have been recorded.[100]
- Ensure notes include what happened, who was there, how it happened, and when it happened. Be specific about the barrier and facilitator (e.g., a specific software program on a specific computer was not working).[100]

☐ As an observer, are there aspects of the setting that you personally find physically, psychologically, or physiologically distracting? How does this affect your data collection?

- Keep track of small talk or routines that may appear to be insignificant at the time (they may be relevant).[100]

☐ Take notes of things that are mentioned at individual, organizational, and system levels.

  – Be open to writing quickly and recording messy notes. As long as an individual observer can interpret the notes, do not focus on spelling and grammar.
  – Avoid using language that makes judgements (e.g., "the desk was filthy"). Instead, be specific about what is observed (e.g., "the desk contains 10 separate stacks of papers and journals").[100]

Considerations for analyzing data

Consider conversations in context

Is there additional contextual information (e.g., body language, eye contact with other team members, sociocultural histories) that may help you further understand what has been observed?

- Consider reviewing observation notes with a participant to avoid any misinterpretations.

☐ Note that depending on the organization, this may require research ethics board approval. Include this approval process in project timelines.

Again, reflect as a team on the following:

☐ What is your team's prior knowledge or experience?

☐ How does your team's background experience influence the way situations are interpreted?

Data management and analysis

Once an observation site visit is complete, record rough field notes immediately and complete them within 24 hours.[102]

When your team has completed the analysis, consider reflecting on how data collection may have been similar or different across observers or different visits.

- Why may this have been the case?
- What can your team learn from this for future observations?

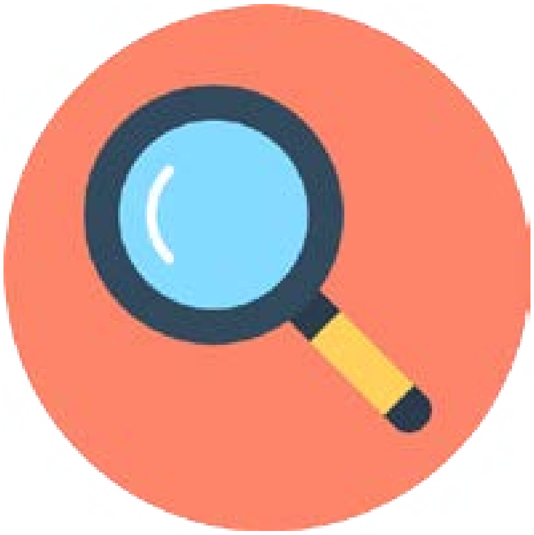

Once the barriers and facilitators assessment is complete

Next Steps

Disseminate results to other members of your implementation team, other members of your organization, and participants who provided information on barriers and facilitators.

- Consider evaluating how the barriers and facilitators assessment went:

☐ Were you able to assess barriers and facilitators?

☐ Did you allow space for all participants to share their stories?

☐ What voices were and were not represented in the barriers and facilitators assessment?

  – Once the barriers and facilitators assessment is complete, the implementation team will use the findings to select, tailor, and implement a KT intervention. For more information on selecting and tailoring KT interventions, see the Selecting and Tailoring KT Interventions Workbook.

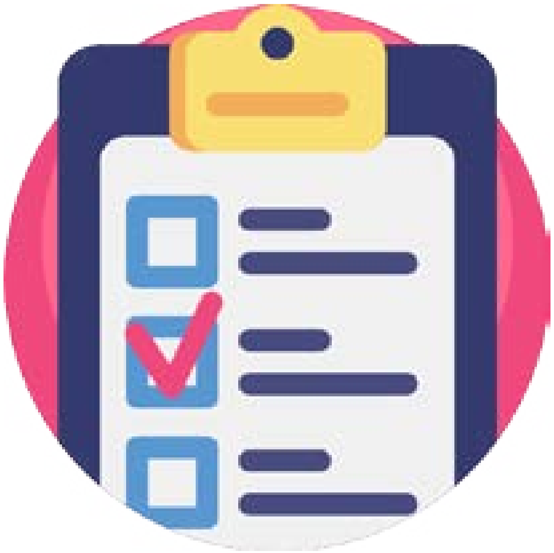

Appendix A: Project Limitations

We acknowledge that the work of our Canadian Institutes of Health (CIHR) - funded team grant was conducted on unceded lands that were the traditional territories of many people, including the Algonquin, Cree, Dakota, Dene, Huron-Wendat, Mississaugas of the Credit River, and the Musqueam Peoples, and on the homeland of the Métis Nation. We acknowledge the harms of the past and the harms that are ongoing. We are grateful for the generous opportunities to conduct work on these lands.

In 2017, the CIHR launched an opportunity for team grants in gender and KT. This opportunity (sponsored by the Institute of Gender and Health) was developed to recognize that the field of KT had yet to thoughtfully integrate gender into its research agenda. The objectives of the CIHR team grant competition were to generate evidence about whether applying sex- and gender-based analysis to KT interventions involving human participants improves effectiveness, thereby contributing to improved health outcomes; contribute to a broader knowledge base on how to effectively and appropriately integrate gender into KT interventions; and facilitate the consideration and development of gender-transformative approaches in KT interventions.

In response to this call, we submitted a grant aimed at helping KT intervention developers use an intersectional approach when designing and implementing interventions to address the needs of older adults. We received feedback from the CIHR peer review committee that substantial concern was raised about our focus on intersectionality. In particular, the Scientific Officer's notes described that the focus on intersectionality would dilute the focus on gender and needed to be reconsidered. A meeting was subsequently held with the successfully funded team and this issue was raised again. We acknowledge the limitation that our intersectional approach comes at the expense of a minimized focus on gender. However, because intersecting categories, such as gender and age, are experienced together, we ultimately elected to use an intersectional approach as it encapsulates the lived experience of those we aim to impact.

A more significant limitation of our work is that we did not include First Nations, Inuit, and Métis community members in the grant proposal. As such, their needs and perspectives were not included in the research grant and, consequently, funded activities. Our team did not have established relationships or expertise in this area and as such, we felt it was inappropriate for our team to work on a grant in this area.

We strongly believe that consideration of gender and KT for Indigenous Peoples should be a primary focus of a distinct team grant.

There are established best practices for community engagement with First Nations, Inuit, and Métis Peoples that begin with principles of collaboration, which take time to develop and must not be tokenistic. The principles for collaboration should ensure authentic engagement, shared respect, trust, and commitment to ensure long-term, mutually empowered relationships. These principles should also ensure that the research-related priorities meet the needs, perspectives, and expectations of the First Nations, Inuit, and Métis Peoples. Indigenous Peoples have a long history of conducting research, and this tradition continues today with many Indigenous healers and scholars leading research in various areas. Indeed, there are many Indigenous scholars working in the KT field.

Because the team's work did not include First Nations, Inuit, and Métis Peoples and involve adhering to the principles that guide their engagement in research, the needs and considerations of these Peoples were not included in the work conducted in this team grant. As such, anyone who is considering using the outputs of this team grant needs to know that **they cannot be broadly applied to these Peoples and there may be other more culturally appropriate models, theories, and frameworks that are useful to consider**. Similarly, because this research focused on older adults (and in particular, chronic disease management in older adults) **it does not apply to children and youth.**

We believe that any KT intervention work needs to begin with engaging the appropriate community and is only applicable when those communities are engaged throughout the research enterprise. Moreover, intersectionality involves deep immersion in the lived experiences and priorities of those communities. As a result, KT work requires immersive work with various populations and not just key informants to ensure the work meets the needs of the relevant populations.

We thank and acknowledge Dr. Lisa Richardson, Co-Lead, Indigenous Health Education, Faculty of Medicine, University of Toronto, for her time and expertise in reviewing this statement.

References

1. Government of Canada. Knowledge Translation in Health Care: Moving from Evidence to Practice - CIHR. 2010. http://www.cihr-irsc.gc.ca/e/40618.html. Accessed May 28, 2019
2. Tannenbaum, C., Greaves, L., & Graham, I.D. Why sex and gender matter in implementation research. BMC Med Res Methodol 2016;16:145. doi: 10.1186/s12874-016-0247-7
3. City of Ottawa and City for All Women Initiative. Advancing Equity and Inclusion – A Guide for Municipalities. CAWI. 2015. http://www.cawi-ivtf.org/sites/default/files/publications/advancing-equity-inclusion-web.pdf. Accessed February 6, 2019.
4. Public Health Agency of Canada. Key Health Inequalities in Canada: A National Portrait – Executive Summary. 2018. https://www.canada.ca/en/public-health/services/publications/science-research-data/key-health-inequalities-canada-national-portrait-executive-summary.html. Accessed February 6, 2019.
5. The Association for Women's Rights in Development. Intersectionality: A Tool for Gender and Economic Justice. Women's Rights and Economic Change. https://lgbtq.unc.edu/sites/lgbtq.unc.edu/files/documents/intersectionality_en.pdf. Published August 2004. Accessed February 4, 2019.
6. Graham I.D., Logan J., Harrison M.B., Straus S.E., Tetroe J., Caswell W., Robinson N. Lost in Knowledge Translation: Time for a Map? J Contin Educ Health Prof. 2006:26(1):13.
7. Collins, P. H. Black feminist thought: knowledge, consciousness, and the politics of empowerment. Routledge. 1990. https://trove.nla.gov.au/version/21207078
8. Crenshaw, K. Demarginalizing the intersection of race and sex: A black feminist critique of antidiscrimination doctrine, feminist theory and antiracist politics. U. Chi. Legal F. 1989;139.
9. Crenshaw, K. A Black feminist critique of antidiscrimination law and politics. The politics of law: A progressive critique. 1990;195.
10. Crenshaw, K. Mapping the Margins: Intersectionality, Identity Politics, and Violence against Women of Color. Stanford Law Review. 1991;43(6), 1241-1299. doi:10.2307/1229039
11. The Learning Network. Issue 15: Intersectionality. 2015. http://www.vawlearningnetwork.ca/sites/vawlearningnetwork.ca/files/Intersectioanlity_Newsletter_FINAL2.pdf. Accessed February 7, 2019.
12. Hankivsky, O. Intersectionality 101. The Institute for Intersectionality Research & Policy, SFU. 2014;1-34. http://vawforum-cwr.ca/sites/default/files/attachments/intersectionallity_101.pdf. Accessed February 7, 2019.
13. Hankivsky, O., Grace, D., Hunting, G et al. An intersectionlaity-based policy analysis framework: critical reflections on a methodology for advancing equity. Int J Equity Health 2014;13(119) doi:10.1186/s12939-014-0119-x.
14. PROGRESS-Plus. Cochrane Equity Methods. https://methods.cochrane.org/equity/projects/evidence-equity/progress-plus. Accessed November 12, 2019.
15. O'Neill, J., Tabish, H., et al. Applying an equity lens to interventions: Using PROGRESS ensures consideration of socially stratifying factors to illuminate inequities in health. Journal of Clinical Epidemiology. 2014;67:56-64.
16. Registered Nurses' Association of Ontario. Toolkit: Implementation of best practice guidelines. 2012. https://rnao.ca/sites/rnao-ca/files/RNAO_ToolKit_2012_rev4_FA.pdf. Accessed May 24, 2019.
17. George & Fay Yee Centre for Healthcare Innovation. Considerations When Building a Budget for Public and Patient Engagement. n.d. https://chimb.ca/sub-sites/1-patient-engagement?page=79-budgeting-for-engagement. Accessed July 2, 2019.
18. International Rescue Committee. Budget template for qualitative methods. 2018. https://www.rescue.org/resource/budget-template-qualitative-methods. Accessed May 24, 2019.
19. Shimmin C, Wittmeier K.D.M., Lavoie J.G., Wicklund E.D., Sibley K.M. Moving towards a more inclusive patient and public involvement in health research paradigm: the incorporation of a trauma-informed intersectional analysis. BMC Health Services Research. 2017;17:539.
20. Alberta Health Services. Trauma Informed Care. N.d. https://www.albertahealthservices.ca/info/Page15526.aspx. Accessed December 29, 2019.
21. Damschroder L, Aron D, Keith R, Kirsh S, Alexander J, Lowery J. Fostering implementation of health services research findings into practice: a consolidated framework for advancing implementation science. Implement Sci. (2009) 4:50. 10.1186/1748-5908-4-50
22. Department of Justice. The USA Patriot Act. https://www.justice.gov/archive/ll/highlights.htm Accessed June 19. 2019.
23. Lillvik, C. Research Guides: The Literature Review: A Research Journey: Overview. N.d. https://guides.library.harvard.edu/c.php?g=310271&p=2071512 Accessed April 11, 2019.
24. Cochrane. What are systematic reviews? 2016. https://www.youtube.com/watch?v=egJlW4vkb1Y. Accessed June 19, 2019.
25. Tricco, A.C., Tetzlaff, J., & Moher, D. The art and science of knowledge synthesis. Journal of Clinical Epidemiology. 2011; 64(1), 11-20.
26. Knowledge Translation Program. JCE Series: Knowledge Synthesis Methods. N.d. https://knowledgetranslation.net/portfolios/jce-series-knowledge-synthesis-methods/. Accessed July 2, 2019.
27. Knowledge Synthesis Team, Knowledge Translation Program. What Review is Right for You? Toronto, Canada. St. Michael's Hospital. 2019. https://whatreviewisrightforyou.knowledgetranslation.net/. September 30, 2019.
28. Welch V, Petticrew M, Tugwell P, Moher D, O'Neill J, Waters E, et al. PRISMA-Equity 2012 Extension: Reporting Guidelines for Systematic Reviews with a Focus on Health Equity. PLoS Med. 2012;9(10): e1001333. doi:10.1371/journal.pmed.1001333
29. Ueffing E, Tugwell P, Welch V, Petticrew M, Kristjansson E for the Campbell and Cochrane Equity Methods Group. Equity Checklist for Systematic Review Authors. Version 2012-10-02. http://equity.cochrane.org/sites/equity.cochrane.org/files/uploads/EquityChecklist2012.pdf Accessed on July 2, 2019.
30. Spencer, A.J., & Eldredge, J.D. Roles for librarians in systematic reviews: a scoping review. J Med Libr Assoc. 2018; 106(1): 46–56.
31. Lefebvre C, Manheimer E, Glanville J. Chapter 6: searching for studies. In: JPT H, Green S, editors. Cochrane Handbook for Systematic Reviews of Interventions. 2011.
32. The Literature Review: A Few Tips On Conducting It | Writing Advice. N.d. https://advice.writing.utoronto.ca/types-of-writing/

literature-review/ Accessed April 11, 2019.

33. Literature Reviews. N.d. https://writingcenter.unc.edu/tips-and-tools/literature-reviews/ Accessed April 11, 2019.

34. Joanna Briggs Institute. EBP. N.d. https://joannabriggs.org/ebp#database. Accessed September 30, 2019.

35. Trip. Medical Database. N.d. https://www.tripdatabase.com/. Accessed September 30, 2019.

36. Cochrane. Cochrane Library. https://www.cochranelibrary.com/cdsr/reviews. Accessed September 30, 2019.

37. NIHR. PROSPERO International prospective register of systematic reviews. N.d. https://www.crd.york.ac.uk/PROSPERO/. Accessed September 30, 2019.

38. PubMed. Home – PubMed – NCBI. https://www.ncbi.nlm.nih.gov/pubmed/. Accessed September 30, 2019.

39. Implementation Science. Implementation Science. https://implementationscience.biomedcentral.com/. Accessed September 30, 2019.

40. Skene, A. Writing a Literature Review. N.d. https://www.utsc.utoronto.ca/twc/sites/utsc.utoronto.ca.twc/files/resource-files/LitReview.pdf Accessed April 11, 2019.

41. Shea Beverley J, Reeves Barnaby C, Wells George, Thuku Micere, Hamel Candyce, Moran Julian et al. AMSTAR 2: a critical appraisal tool for systematic reviews that include randomised or non-randomised studies of healthcare interventions, or both BMJ 2017; 358 :j4008

42. Joanna Briggs Institute. Critical Appraisal Tools. N.d. https://joannabriggs.org/critical_appraisal_tools. Accessed July 2, 2019.

43. Critical Appraisal Skills Programme. CASP Appraisal Checklist. 2018. https://casp-uk.net/casp-tools-checklists/. Accessed July 2, 2019.

44. O'Brien, B. C., Harris, I. B., Beckman, T. J., Reed, D. A., & Cook, D. A. Standards for reporting qualitative research: a synthesis of recommendations. Academic Medicine, 2014;89(9), 1245-1251.

45. Mayo, E. The human problems of an industrial civilization. Routledge; 1933.

46. Cohen D, Crabtree B. "Qualitative Research Guidelines Project." Updated July 2006. http://www.qualres.org/HomeInfo-3631.html Accessed April 17, 2019.

47. Changing Minds. Open and Closed questions. N.d. http://changingminds.org/techniques/questioning/open_closed_questions.htm Accessed June 19. 2019.

48. Garzon, J. Key Tips for Active Listening. n.d. https://hr.mit.edu/learning-topics/comm/articles/active-listening Accessed June 19, 2019.

49. Jagosh, J., et al. A realist evaluation of community-based participatory research: partnership synergy, trust building and related ripple effects. BMC public health. 2015;15(1), 725.

50. Both, R., & Samuel, F. Keeping silent about emergency contraceptives in Addis Ababa: a qualitative study among young people, service providers, and key stakeholders. BMC women's health, 2014;14(1), 134

51. Jacob, W.J., & Alvarado, F. A Guide to Education Project Design Based on a Comprehensive Literature and Project Review. USAID. n.d. https://www.epdc.org/sites/default/files/documents/EQUIP2%20SOAK%20-%20Policy%20Dialogue.pdf Accessed April 17, 2019.

52. Gray DE. Doing Research in the Real World. 2nd ed. Thousand Oaks, California: Sage Publications; 2009.

53. FAO. Chapter 5: Personal Interviews. n.d. http://www.fao.org/3/w3241e/w3241e06.html Accessed April 23, 2019

54. Government of Canada, S. of W. C. Gender-based Analysis Plus (GBA+) - Take the GBA+ course - the Department for Women and Gender Equity. Updated September 26, 2018. https://cfc-swc.gc.ca/gba-acs/course-cours-en.html Accessed February 4, 2019.

55. Turner III, D. W. Qualitative interview design: A practical guide for novice investigators. The qualitative report, 2010;15(3), 754-760.

56. Kitzinger, J. Qualitative research: introducing focus groups. BMJ, 1995;311(7000), 299-302.

57. Medecins Sans Frontieres. A Guide to Using Qualitative Research Methodology. 2002. https://evaluation.msf.org/sites/evaluation/files/a_guide_to_using_qualitative_research_methodology.pdf. Accessed June 19, 2019.

58. Hunting, G. Intersectionality-informed Qualitative Research: A Primer. The Institute for Intersectionality Research & Policy, SFU. 2014.

59. Windsong, E.A. Incorporating intersectionality into research design: an example using qualitative interviews, International Journal of Social Research Methodology, 2018; 21(2), 135-147, DOI: 10.1080/13645579.2016.1268361

60. Equality Challenge Unit. Intersectional approaches to equality research and data. N.d. https://www.ecu.ac.uk/publications/intersectional-approaches-to-equality-research-and-data/ Accessed June 19, 2019.

61. Yale University. Fundamentals of Qualitative Research Methods: Focus Groups (Module 4). https://www.youtube.com/watch?v=cCAPz14yjd4. Accessed June 19, 2019.

62. Canadian Institutes of Health Research. Considerations when paying patient partners. 2019. http://www.cihr-irsc.gc.ca/e/51466.html. Accessed July 2, 2019.

63. National Implementation Research Network. Interview Tips. 2013. https://nirn.fpg.unc.edu/ai-hub Accessed June 19, 2019.

64. National Implementation Research Network. Interview Video Examples. N.d. https://tinyurl.com/y43n494q Accessed June 19, 2019.

65. Arthritis Research Canada. Workbook to guide the development of a Patient Engagement In Research (PEIR) Plan. 2018. http://www.arthritisresearch.ca/wp-content/uploads/2018/06/PEIR-Plan-Guide.pdf. Accessed on July 2, 2019.

66. Access Alliance. Everyone can do research. 2013. https://accessalliance.ca/wp-content/uploads/2016/12/Everyone-can-do-research-toolkit-May-2013.pdf. Accessed June 19, 2019.

67. Bradley, E.H., Curry, L.A., & Devers, K.J. Qualitative Data Analysis for Health Services Research: Developing Taxonomy, Themes, and Theory. Health Research and Educational Trust. 2007. Doi: 10.1111/j.1475-6773.2006.00684.x

68. Rozmovits, L. Coding Qualitative Data. Workshop presented at the Centre for Critical Qualitative Health Research in University of Toronto. September, 2011; Toronto, ON.

69. Hankivsky, O. & Grace, D. Understanding and emphasizing difference and intersectionality in mixed and multi method research. The Oxford Handbook of Mixed and Multimethod Research. 2015.

70. Gale et al. Using the framework method for the analysis of qualitative data in multi-disciplinary health research. BMC Med Res Method. 2013;13:117.

71. QSR International. NVivo. N.d. https://tinyurl.com/y5alm69x Accessed May 31, 2019.

72. Raven's Eye. 2018. https://tinyurl.com/y5mkxlkh Accessed May 31, 2019.

73. Dedoose. N.d. https://tinyurl.com/yyg3lmdt Accessed May 31, 2019.

74. MAXQDA. N.d. https://tinyurl.com/y6x29o4s Accessed May 31, 2019.

75. Tong A, Sainsbury P, Craig J. Consolidated criteria for reporting qualitative research (COREQ): a 32-item checklist for interviews and focus groups. International Journal for Quality in Health Care. 2007. Volume 19, Number 6: pp. 349 – 357

76. Hoddinott, S. N., & Bass, M. J. The Dillman total design survey method. Canadian family physician, 1986; 32, 2366.

77. Check, J., & Schutt, R. K. Survey research. Research methods in education, 2012; 159-185.

78. What is a Survey? Definition & Free Tool. Qualtrics. n.d. https://www.qualtrics.com/experience-management/research/survey-basics/ Accessed April 16, 2019.

79. Columbia College. Cultural Diversity Self-Assessment Survey. N.d. http://tinyurl.com/y5tolxy9 Accessed June 19, 2019.

80. Texas Department of State Health Services. Dillman's Principles for Questionnaire Construction. N.d. http://tinyurl.com/yyylkdq7 Accessed June 19, 2019.

81. Rouhani, S. Intersectionality-informed Quantitative Research: A Primer. The Institute for Intersectionality Research & Policy, SFU. 2014.

82. Bauer, G.R. & Schiem, A.I. Methods for analytic intercategorical intersectionality in quantitative research: Discrimination as a mediator of health inequalities. Social Science & Medicine. 2019;226, 236-245.

83. RAND. Guidelines for designing and implementing internet surveys. N.d. https://www.rand.org/content/dam/rand/pubs/monograph_reports/MR1480/MR1480.ch5.pdf. Accessed April 16, 2019.

84. Cole, E. R. Intersectionality and Research in Psychology, American Psychologist. 2009;64(3), 170-180.

85. Government of Canada, S. of W. C. Gender-based Analysis Plus (GBA+) – Research Guide - the Department for Women and Gender Equity. Updated July 18, 2017. https://cfc-swc.gc.ca/gba-acs/guide-en.html#section4 Accessed May 15, 2019.

86. Dillman D. A., Smyth J. D., Christian L. M. Internet, phone, mail, and mixed-mode surveys: The tailored design method. Hoboken, NJ: John Wiley & Sons, Inc; 2014.

87. Dillman, D. A. Mail and internet surveys: The tailored design method 2nd ed. John Wiley & Sons Inc; 2007.

88. Dillman D.A. The promise and challenge of pushing respondents to the Web in mixed-mode surveys. Survey Methodology. 2017;43(1), 3-30.

89. Dillman DA. Mail and telephone surveys: The total design method: Wiley; 1978.

90. SurveyMonkey. https://www.surveymonkey.com. Accessed September 30, 2019.

91. Qualtrics. https://www.qualtrics.com/. Accessed September 30, 2019.

92. REDCap. https://www.project-redcap.org/. Accessed September 30, 2019.

93. Google Forms. https://www.google.com/forms/about/. Accessed September 30, 2019.

94. The Learning Network. Issue 15: Intersectionality. N.d. http://www.vawlearningnetwork.ca/our-work/issuebased_newsletters/issue-15/index.html Accessed May 16, 2019.

95. Bowling, A. Research methods in health: investigating health and health services. UK. McGraw-Hill Education; 2014.

96. Research Methods and Statistics. 2.2. Participant Observation 2016. http://tinyurl.com/yyx7ev5v Accessed June 19. 2019.

97. Wang, C., & Burris, M. A. Photovoice: Concept, Methodology, and Use for Participatory Needs Assessment. Health Education & Behavior. 1997;24(3), 369–387.

98. Community Tool Box. Assessing Community Needs and Resources | Section 20. Implementing Photovoice in Your Community | Main Section | N.d. https://ctb.ku.edu/en/table-of-contents/assessment/assessing-community-needs-and-resources/photovoice/main Accessed May 24, 2019.

99. Fitzpatrick, A. L., Steinman, L. E., Tu, S.-P., Ly, K. A., Ton, T. G. N., Yip, M.-P., & Sin, M.-K.. Using Photovoice to Understand Cardiovascular Health Awareness in Asian Elders. Health Promotion Practice. 2012;13(1), 48–54. https://doi.org/10.1177/1524839910364381

100. Neuman, W.L. Social research methods qualitative and quantitative approaches. 4th Edition, Allyn & Bacon; 2000.

101. Office of the Vice-President, Research and Innovation. Site visit overview. University of Toronto. 2014. http://www.research.utoronto.ca/wp-content/uploads/documents/2014/09/PAR-Site-Visit-Overview.pdf Accessed April 16, 2019.

102. Keith RE, Crosson JC, O'Malley AS, Cromp D, Taylor EF. Using the Consolidated Framework for Implementation Research (CFIR) to produce actionable findings: a rapid-cycle evaluation approach to improving implementation. Implement Sci. 2017;12(1):15. doi:10.1186/s13012-017-0550-7.

Intersectionality & Knowledge Translation (KT)

Selecting and Tailoring KT Interventions Workbook

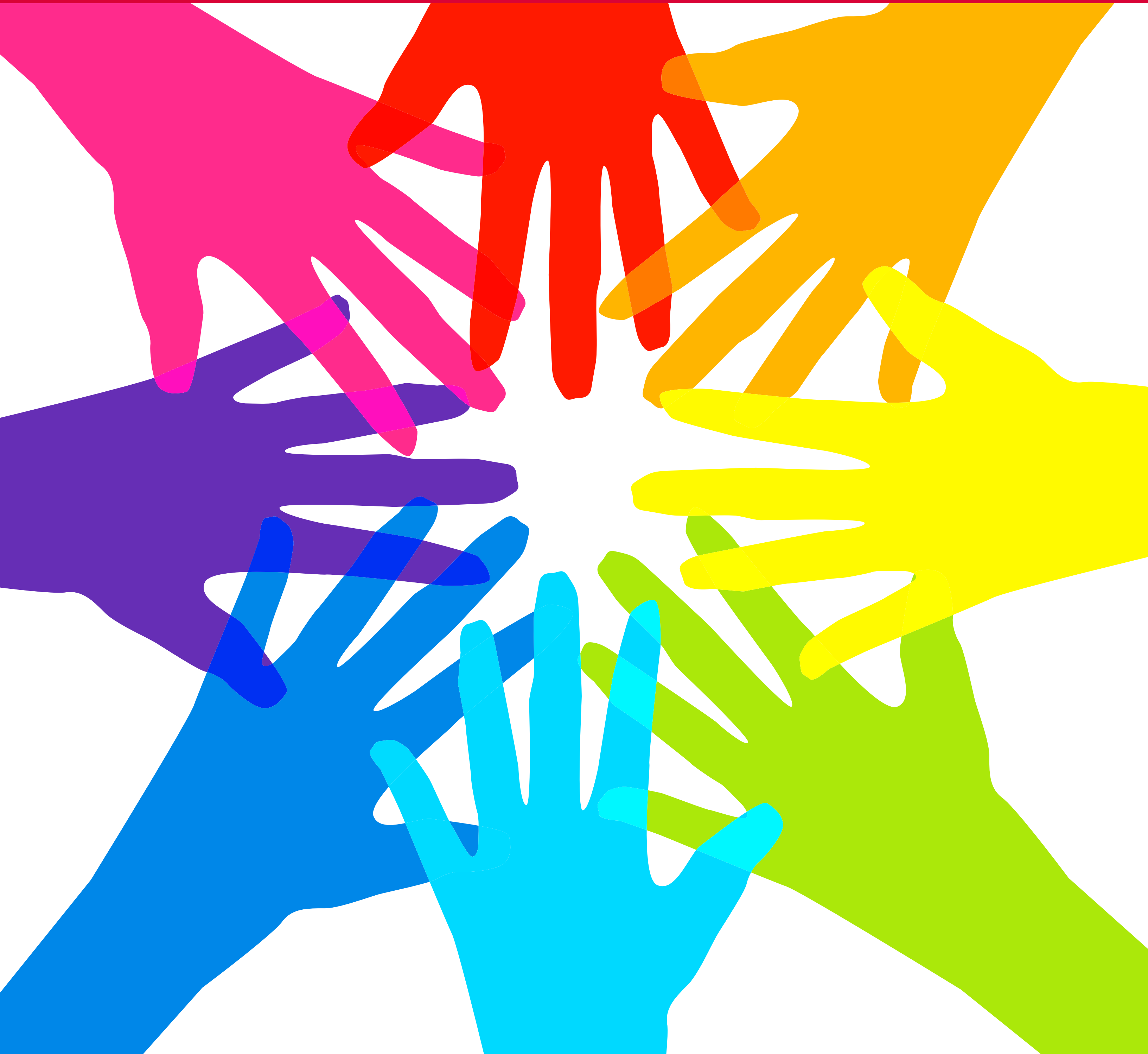

Table of Contents

**Introduction**	**3**
**Mobilization of Vulnerable Elders (MOVE) case example**	**6**
**Activity 1: Clarifying the "what" and "who" for your KT project with an intersectional lens**	**7**
**Selecting KT interventions**	**9**
**Activity 2: Summarizing barriers, facilitators, and intersectionality considerations for your KT project**	**11**
**When selecting a KT intervention, consider the APEASE criteria**	**12**
**Case example**	**13**
**Activity 3: Selecting KT interventions for your project using APEASE**	**14**
**Tailoring KT interventions**	**15**
**Case example**	**18**
**Activity 4: Tailoring KT interventions for your project**	**20**
**Once you have selected and tailored KT strategies:**	**21**
**Appendix A: Implementing KT interventions**	**22**
**Appendix B: Project limitations**	**23**
**References**	**24**

Introduction

Who is this workbook for?

Knowledge translation (KT) intervention developers. KT is the process of moving evidence into health care practice.[1] KT intervention developers are people who create KT interventions designed to improve health care.

For example, an intervention developer may design a KT intervention to encourage physiotherapists to use a patient physical activity program. The KT intervention may include restructuring physiotherapists' workflow and delivering in-person education sessions.

Project managers responsible for selecting and tailoring KT interventions would find this workbook particularly useful.

Why should I use this workbook?

This workbook is essential because it provides a structured way to consider key elements of selecting and tailoring a KT intervention with an intersectional lens.

By applying an inclusive and equitable lens to KT interventions, you can design more effective interventions that address the complex realities of the people you work with.[2-5] For more, see section on "Why should I consider intersectionality when selecting and tailoring KT interventions".

What is the purpose of this workbook? When do I use it?

This tool relates to the "Select, Tailor, and Implement KT Interventions" stage of the Knowledge-to-Action (KTA) Cycle.[6] In particular, it focuses on selecting and tailoring interventions. **Appendix A** outlines additional resources on implementing KT interventions.

When thinking about selecting, tailoring, and implementing KT strategies, you can also think about evaluating your efforts. This tool is part of a set of tools that will help you take an intersectional approach when doing KT.

Who made this workbook?

This tool was collaboratively developed in an iterative fashion by an interdisciplinary team of KT scholars, KT intervention developers, intersectionality scholars, and adult education experts.

How do I use this workbook?

This workbook should be used as a resource and discussion tool. It is meant to prompt reflection; it is not meant to be prescriptive.

Each person working on the KT project can work through their own copy of the workbook. If team members feel comfortable, they can share and discuss their responses with the team.

The workbook contains reference materials, examples, and activities.

As you work through this workbook, you will review the case example of the Mobilization of Vulnerable Elders (MOVE) project.[7] Although the overall MOVE project is a real-life example, the scenarios describing the experiences of "Unit 2A" are not real and have been crafted for the purposes of this workbook.

Project limitations

See Appendix B for a project limitation statement.

This tool cannot be broadly applied to Indigenous Peoples, and there may be more culturally appropriate models, theories, and frameworks that are useful to consider when conducting projects that involve Indigenous communities.

For more information

This tool is part of a set of tools that help us take an intersectional approach when doing KT. Consult the tools below for more information on key topics.

- Intersectionality, definitions, and running a KT project with an intersectional approach: Intersectionality Guide.
- An intersectional approach to identifying knowledge-to-practice gap(s) and practice change(s) to fill these gaps: Reflection Workbook.
- An intersectional approach to conducting a barriers and facilitators assessment: Guide for Common Approaches to Assessing Barriers & Facilitators to Knowledge Use.

Key terms

Intersectionality* is a way of looking at the world that recognizes that people's experiences are shaped by a combination of social factors, including their gender, racialization, age, among others.[8-14] These experiences occur within and interact with connected systems and structures of power, such as sexism and racismm.[8-14]

Note that there are various definitions of intersectionality and that they are evolving.

Intersecting categories include age, gender identity, sex, and other aspects of one's lived experience. These aspects interact to form a person's identity (See Figure 1)..[3,13,14] One's intersecting identities reflect larger systems of oppression/privilege (e.g., sexism, ageism).[3,13,14]

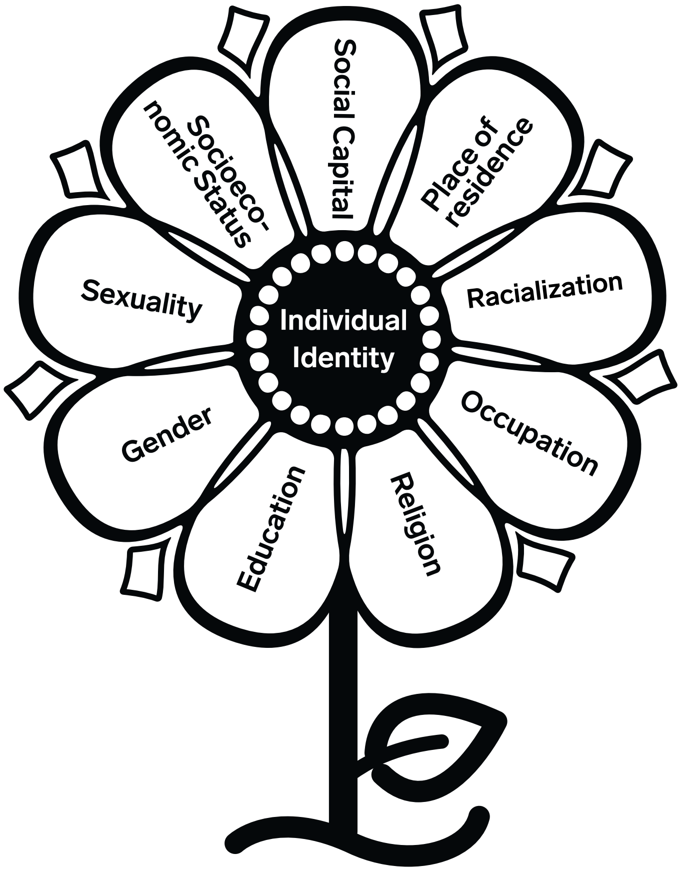

Figure 1. Visual representation of some intersecting categories.[13,15,16] The categories mentioned in this figure are not an exhaustive list.

Knowledge translation is the process of getting evidence used in practice.[1]

Selecting a KT intervention is the process of choosing which KT intervention(s) will be used to get evidence used in practice.

Tailoring a KT intervention is the process of adapting a KT intervention(s) to a specific context.

Reflections before using this tool

Consider the intersecting categories that make up your identity, your team's identity, and those of your target audience. Reflect on how acknowledging unique experiences is important in developing an effective KT intervention.

Explore your biases (your preconceived judgments for or against a particular individual or group).[17] See these free resources:

- **Harvard -** Project Implicit: https://tinyurl.com/6yyyc[18]
- **Government of Canada -** Unconscious bias training module https://tinyurl.com/yacj5ao3[19]
- **EdX -** Unconscious Bias: From Awareness to Action https://tinyurl.com/yxk5lmb2[20]

It is important to plan for sustainability when selecting and tailoring an intervention. Sustainability is the continuation of an intervention to achieve desired long-term change. For more on sustainability, visit https://tinyurl.com/y4vnwj5j[21]

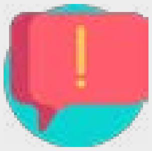

Please note: Taking an intersectional approach is needed to recognize the importance of individuals' social identities within the greater context of systems and structures of power. These reflect macro systems of privilege and oppression. Keep in mind that recognizing areas of advantage, disadvantage, and oppression may bring up feelings of confusion, guilt, distress, among others. It is okay to feel uncomfortable. There is a difference between feeling uncomfortable and unsafe.

Why should I consider intersectionality when selecting and tailoring KT interventions?

There are many intersectionality considerations that influence the success of a KT intervention. You can select and tailor KT interventions to be more effective by being mindful of these factors.

The factors and examples below are key elements to consider when building a KT intervention:

**Sociopolitical**[22]	• Laws (e.g., Canadian Human Rights Act)<br>• Historical context (e.g., slavery)<br>• Political context (e.g., what political group is in power)<br>• Societal/cultural norms (e.g., different health-seeking behaviours displayed by people from different cultures/countries)
**Organization/Setting**[22]<br>*each organization has its own culture*	• Available resources (e.g., funds, technology)<br>• Competing demands or mandates (e.g., pressure to cut costs)<br>• Billing constraints (e.g., ability to bill for certain International Classification of Disesases codes)<br>• Cultural norms (e.g., learning)
**Provider**[22]<br>*e.g., nurse, educator, physician*	Examples of intersecting categories:<br>• Ethnicity<br>• Gender identity<br>• Spoken languages<br>• Previous training and skills<br>• Religion<br>• Cultural beliefs
**Recipient**[22]<br>*e.g., patient group*	Examples of intersecting categories:<br>• Ethnicity<br>• Literacy<br>• Education level<br>• Spoken languages<br>• Immigration/legal status<br>• Crisis or emergent circumstances<br>• Religion<br>• Access to resources<br>• Disability<br>• Racialization<br>• Gender<br>• Sexuality<br>• Sex

Mobilization of Vulnerable Elders (MOVE) case example[7]

Identifying the Problem:

In the late 2000s, the Division of Geriatric Medicine at the University of Toronto, along with collaborators, reviewed evidence relating to successful aging.[7] The team noted that keeping older adults physically active while in hospital improved older adults' functional status after they left the hospital.[7]

After reviewing administrative data, the Geriatric Medicine team found that many elderly patients admitted to acute care hospitals in Ontario were confined to their beds or chairs while in the hospital.[7] Accordingly, the Geriatric Medicine team identified the problem of not keeping older adults physically active while in hospital.[7]

The Geriatric Medicine team, along with staff at four Ontario hospitals, formed a KT intervention development team to address this problem in different units across four hospitals.

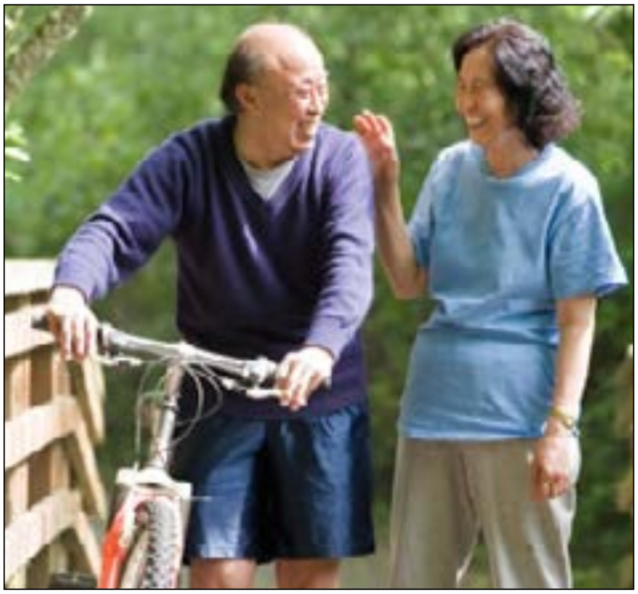

Two older adults walking in a park. Both have smiles on their faces and one is pushing a bike. In: Word, Microsoft Office Professional Plus, version 14.0.7232.5000, Microsoft, 2010.

Defining the Evidence-to-Practice Gap and Selecting Practice Changes on Unit 2A:

From a chart review of older adults admitted to Unit 2A, the KT intervention development team noted that 40% of older adults admitted to the unit were not physically active.

To increase rates of physical activity among older adults admitted to Unit 2A, the Geriatric Medicine team identified asessing mobility within 24 hours of a patient's admission as a practice change from credible evidence.[7]

Linking Barriers and Facilitators to KT Interventions on Unit 2A

At each of the four hospitals, the KT intervention development team investigated the barriers and facilitators for different practice changes on different hospital units. The KT intervention development team used surveys and interviews with relevant individuals, including nurses on different hospital units.

For Unit 2A at one hospital, the largest barrier to the practice change of assessing patients' mobility within 24 hours of admission was nurses' beliefs about what happens when older adults are mobilized. Specifically, nurses believed that if they mobilized older patients on their unit, the patients would be more likely to fall. The nurses did not want to cause harm and wanted to adhere to the hospital's falls prevention policies. Knowing this barrier, the KT intervention development team selected and tailored a KT intervention to target nurses' beliefs about the consequences of mobilization.

Within this workbook, you will explore the process the KT intervention development team used to select and tailor a KT intervention.

Although the KT intervention development team identified a suite of barriers and facilitators that need to be considered, this workbook will focus on one barrier - nurses' beliefs about what happens when older adults are physically active in hospitals.

Activity 1: Clarifying the “what” and “who” for your KT project with an intersectional lens[23]

Begin this workbook by reflecting on questions related to your current project. Fill in the blank third column, and be as specific as possible.

The MOVE case example is included for reference in the middle column on how you can answer the questions outlined in the first column.

**What is the current practice?**	Patients’ mobility is not assessed upon admission or within 24 hours of admission. Mobility may be assessed at a later point for specific clinical cases.	
**What is the behaviour you want to change?*** <br>**How will the current practice be changed:** • **Stopped** • **Replaced** • **Modified** • **Added to**	Assessing mobility within 24 hours of a patient’s admission. <br>The current practice will be modified.	

*A KT project may involve multiple practice changes. Fill out this table for each practice change.

Questions to Ask	Mobilization of Vulnerable Elders (MOVE) Case Example (Unit 2A)	Your KT Project
**Who is changing their behaviour?****	Nurses	
**What are key intersecting categories as identified by those expected to change their behaviour?**<br><br>Refer to Figure 1 for a conceptualization of intersecting categories.<br><br>*Note that the number of intersecting categories to consider will depend on the project.*<br><br>*For more information on exploring intersecting categories, please visit the* [Intersectionality Guide]	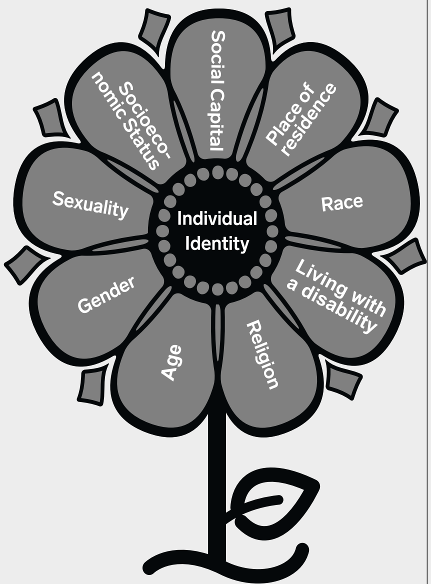	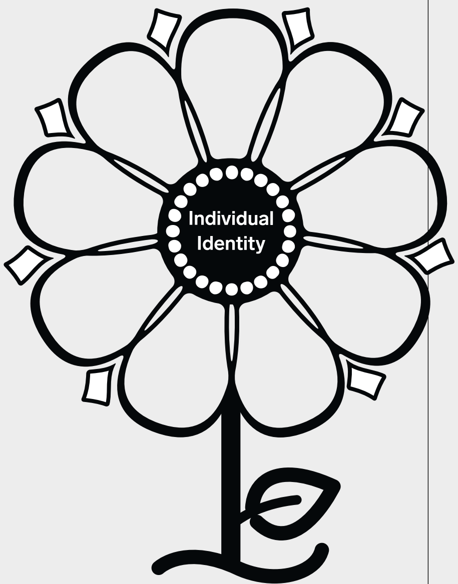<br>Fill in the blank petals with relevant intersecting categories of the indviduals changing their behaviour.

**There can be many “whos” (e.g., nurses, doctors, administrators, patients). Complete a table for each group that will be making a behaviour change.

Selecting KT interventions

The selected KT intervention(s) should directly address the barriers and facilitators to knowledge use that you have identified.

You can use a behaviour change theory to identify KT interventions that address the barriers and facilitators to knowledge use that you have identified.

For example, you can use the Intersectionality-Enhanced Behaviour Change Wheel (BCW; Figure 2).

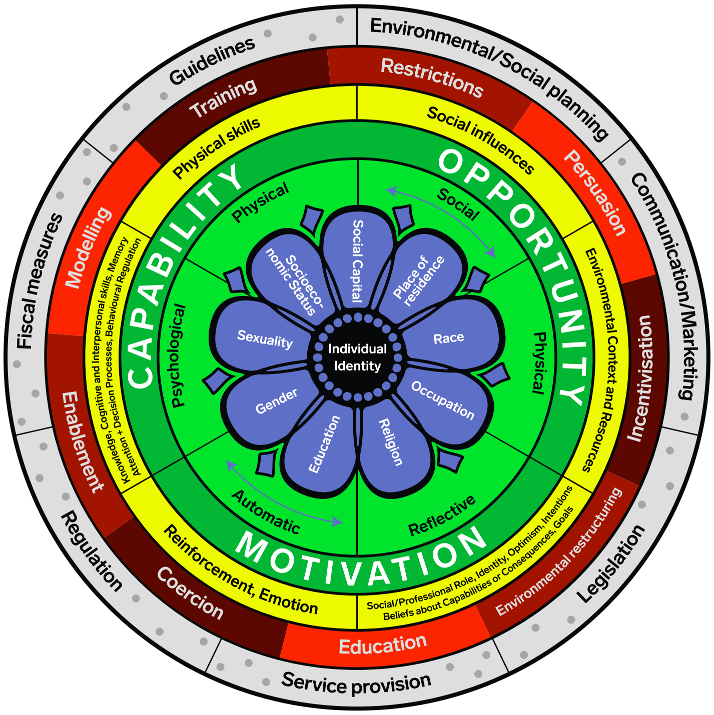

Behaviour Change Wheel Legend

●	Intersecting Categories of Individuals Expected to Change Their Behaviour*
●	Sources of Behaviour
●	Theoretical Domains Framework
●	Intervention Functions
●	Policy Categories

*Because these categories and their interactions are not static, only this layer rotates. The intersecting categories depicted in the flower are not an exhaustive list. Reflect on what intersecting categories may be relevant for each situation.

Figure 2. Intersectionality-Enhanced Behaviour Change Wheel[24,25]

You can use the BCW to select KT interventions that best target specific barriers and facilitators to behaviour change. When using the BCW, you first reflect on constructs in the middle of the wheel. Then, you move outwards.

The BCW has one mobile layer and four stationary layers, including the following:

1. The **intersecting categories** layer is placed at the center of the BCW. Because all layers of the BCW are influenced by intersecting categories, a user must first consider individual identities and intersecting categories.
   - The budding "petals" on the outside of the image signify that there are intersecting categories you may not be aware of.
   - Because intersecting categories and their interactions are not static, this layer rotates.
2. The **capability, opportunity, and motivation (COM-B)** layer outlines the central "behaviour system," which highlights the three essential factors that generate behaviour.
3. The **theoretical domains framework (TDF) layer** outlines the cognitive, affective, social, and environmental barriers and facilitators to behaviour change.
4. The **intervention functions** layer outlines KT interventions that are likely to target the barriers/facilitators established in previous layers.
5. The **policy categories** layer outlines the type of policies that can support the implementation of the selected KT intervention(s).

For example, from a barriers/facilitators assessment, you know that **education and disability** impact nurses' **physical capability** to do a behaviour. Moving from the center of the BCW outwards, you identify the TDF domain of **physical skills** as a barrier to behaviour change. Accordingly, you consider **training** as a KT intervention function that is likely to address this barrier. If you are investigating policy categories, you could consider **guidelines**.

For more information

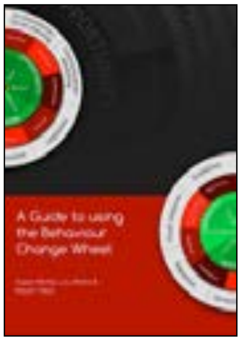

For more on selecting behaviour change technique(s), consult Michie, Atkins, and West (2014) The Behaviour Change Wheel: A guide to designing interventions.[24]

To read more about the creation of the Behaviour Change Wheel, visit http://tinyurl.com/yxvn26z9[26]

Note that as you work on selecting a KT intervention, you should also plan your evaluation and the sustainability of the intervention. For more information on evaluation, see the RE-AIM framework, a commonly used framework in evaluation: https://tinyurl.com/y5wsta5o[27]

Team Reflection

There are often multiple KT interventions you can select, take a moment to reflect:

- How will your team prioritize KT interventions?
- How wil your team consider the intersecting categories of the individuals changing their behaviour?
- How will your team consider systems and structures of power that impact the behaviour change?

For more information on popular prioritization techniques, visit: https://tinyurl.com/snwws6p[28]

Selecting KT Interventions: Mobilization of Vulnerable Elders (MOVE) Case Example[7]

Using the intersectionality-enhanced behaviour change wheel (Figure 2), Unit 2A's KT intervention development team mapped the barrier "beliefs about consequences" to the intervention functions "environmental restructuring" and "education."

From the barriers and facilitators interviews with nurses on the unit, the KT intervention development team also noted that the nurses' intersecting categories of age and education level were particularly important. Nurses who identified as younger held a strong belief that moving older adults would result in more falls. Nurses who had completed graduate school training were less likely to hold this belief.

Activity 2: Summarizing barriers, facilitators, and intersectionality considerations for your KT project

Fill in the blank column related to your KT project. Be as specific as possible.

**What barriers to behaviour change did you identify?**<br>These can be identified through knowledge syntheses, conversations with stakeholders, interviews/focus groups, surveys, and observations. For more information, see the Guide for Common Approaches to Assessing Barriers & Facilitators to Knowledge Use.	Belief that mobilizing patients will lead to more falls.	
**Who is changing their behaviour?***	Unit 2A nurses	
**What does an intersectional approach tell us about these barriers?**<br>Think through how you can identify barriers and their related context	The education system (e.g., undergraduate nursing education) and organizational context (e.g., falls prevention policies at the hospital) support the belief that mobilizing patients will lead to patients falling.<br>Middle-aged female nurses, who have historically held roles as caregivers to aging relatives, share stories of how mobilizing family members has led to falls.	
**What facilitators to knowledge use did you identify?**	Nurses' desire to improve patient outcomes. Nurses' desire to be in compliance with hospital's falls prevention policies.	
**What does an intersectional approach tell us about these facilitators?**	Nurses' motivation to provide quality care is driven by the intersection of their professional role, individual values, and societal norms.<br>Nurses' role as paid employees of the organization impacts their desire to comply with existing organizational mandates (e.g., falls prevention initiatives).	

*There can be many "whos" (e.g., nurses, doctors, administrators, people with lived experiences). Complete a table for each group that will be making a behaviour change.

When selecting a KT intervention, consider the APEASE criteria[29]

When reviewing and selecting KT interventions, you need to consider each intervention's affordability, practicability, effectiveness/cost-effectiveness, acceptability, side effects/safety, and equity (APEASE).[29]

You can also consider each of the APEASE criteria with an intersectional lens. Exploring these considerations may involve additional conversations with members of the implementation team; members of the group(s) changing their behaviour, which may include patients; and organizational leadership, among others.

**Affordability**	Can it be delivered on budget?[29]	Are budgets for accommodations (e.g., American Sign Language [ASL] interpreters, bus tickets, and translators) included in the budget?
**Practicability**	Can it be delivered as designed?[29]	Is the intervention suitable for participants with a range of intersecting categories (e.g., living with a disability)? Do these individuals perceive the intervention as practical?
**Effectiveness and Cost-Effectiveness**	Does it work (is the effect to cost ratio worth it)?[29]	Is the intervention effective for individuals who represent a range of intersecting categories?
**Acceptability**	Is it appropriate from the perspective of stakeholders (including those changing their behaviour, patients, and organizational leadership)?[29]	Can the intervention be tailored to individuals with a range of intersecting categories?
**Side Effects/Safety**	Are there unintended side effects or safety concerns?[29]	Are there safety concerns for individuals from certain groups?
**Equity**	Will it impact disparities in health and well-being? Is the impact likely to be positive or negative?[29]	Has the impact of the intervention been considered for individuals with different intersecting categories (e.g., how the intervention impacts a racialized queer woman vs. a male with a physical disability)?

Case example

Selecting KT interventions using APEASE: Mobilization of Vulnerable Elders (MOVE)

After the intervention function "education" was established as important in forming beliefs about the consequences of mobilization, the KT intervention development team explored a range of education intervention options.

By speaking with a colleague who worked on the MOVE KT intervention development team for another unit, the KT intervention team for Unit 2A learned about online evidence-based educational materials (including learning modules, handouts, and educational materials) on mobilization and its benefits.

The KT intervention development team for Unit 2A applied the APEASE criteria for the available e-learning modules (see table below). Four nurses were interviewed to understand the APEASE criteria through an intersectional lens.

**Affordability**	Because the e-learning modules had already been created, the KT intervention fit within the KT intervention development team's budget. There are two nurses who have vision difficulties. The e-learning module is provided in large font size and compatible with screen readers, which enables their participation.
**Practicability**	E-learning modules can be reasonably completed in the number of professional development hours allotted for nurses. Some nurses work flexible schedules to meet caregiving needs (e.g., leave one hour early every other shift to care for a family member). Modules can be stopped and started to fit these flexible schedules. The e-learning modules are also compatible with the computers on Unit 2A.
**Effectiveness and Cost-Effectiveness**	By contacting the MOVE Canada research coordinators, the KT intervention development team for Unit 2A discovered that the e-learning module had been shown to be effective for changing beliefs about the consequences of movement among individuals working in other units. Other units also identified that hospital administrators had found the modules to be a cost-effective strategy.
**Acceptability**	Nurses on Unit 2A routinely complete online learning modules for training purposes.
**Side Effects/Safety**	Some nurses on Unit 2A have experienced vision difficulties when looking at computer screens for extended periods of time. The KT intervention development team confirmed that the modules can be started/stopped based on participants' preference. An individual can start/stop a module as many times as they would like within one calendar year of starting.
**Equity**	The course is provided in English only and features examples from mostly white, female nurses. The KT implementation team believes that not all nurses will identify with the examples given. Some nurses have difficulty hearing, although the module is available with captions. All nurses speak English, but some take more time than others to read English text. All nurses have computer access to hospital library computers.

Activity 3: Selecting KT interventions for your project using APEASE[29]

Fill in the blank columns related to the APEASE criteria for your KT project.[29] **You can use the intersectionality considerations outlined in the third column to help think through each APEASE criterion.**

If there is more than one KT intervention you are considering, fill out the table below for each of these KT interventions. Be as specific as possible.

Affordability	The e-learning module is provided in large font size and is compatible with screen readers, which enables everyone's participation.	
Practicability	E-learning modules can be finished based on the flexible schedule needs of staff (e.g., family caregiving needs).	
Effectiveness and Cost-Effectiveness	Hospital administrators viewed e-learning modules as a cost-effective method to change individual behaviours.	
Acceptability	Nurses on Unit 2A routinely complete online learning modules for training purposes.	
Side Effects/Safety	Some nurses have vision difficulties. An individual can start/stop a module as many times as they would like within one calendar year of starting.	
Equity	Library computers at the hospital are accessible to all nurses, and captions are provided in the modules.	

Tailoring KT interventions

Once you have selected your KT intervention, you need to tailor it to your project. Tailoring a KT intervention is a planned personalization for a specific context. For example, it can be to create educational materials in English and French to fit clinicians' language skills.[30]

Using an intersectional lens, you can tailor interventions to be more affordable, practical, effective/cost-effective, acceptable, safe, and equitable (APEASE).[22,25,29]

While tailoring your KT interventions, consider the context of connected systems and structures of power (e.g., laws, the media) that interact with and influence the intersecting categories of the individuals involved.

Who to tailor for?

**Patient Level**	Using diagrams or visual materials to outline examples of exercises suitable for patients in a hospital who speak English as an additional language.	Are there key intersecting categories (e.g., age, gender, and location) that are relevant to tailor examples to? How can you make these examples more inclusive?<br>What can you do to redistribute power in the discussion between those in the intervention?<br>What assumptions are you making about the patient experience?<br>If you cannot tailor to each subgroup, how will you prioritize which subgroups to tailor to?
**Practitioner Level**	A specific psychotherapist who specializes in cognitive behavioural therapy (CBT) does not use a printed version of a goal-setting sheet when seeing patients.	How does an individual's intersecting categories influence their work? How can you support every practitioner's participation in the intervention?
**Organization Level**	A physiotherapy clinic does not offer 45-minute sessions. It offers 30-minute sessions.	Is there a way that the clinic/unit is structured that may benefit certain practitioners or patients? For example, the organization has a norm of "being consistent in the office," which penalizes those who use flexible hours for their caregiving roles.

Level of Tailoring[22]	Example[22]	Examples of Intersectionality Considerations
**Network Level**	A group of community clinics has the same amount of protected time for professional development. Educational KT interventions must fit within this amount of time.	Though everyone in the network is granted the same amount of protected time, compared to older physicians, do younger nurses across the network use this protected time? What social structures might impact staff's utilization of protected time?
**System Level**	The **Accessibility for Ontarians with Disabilities Act** requires all Ontario hospitals to make general accessibility provisions.	How do overt and hidden societal biases impact the implementation of legislation like The Accessibility for Ontarians with Disabilities Act? How is this legislation enforced?<br><br>How do these societal biases impact knowledge use? For example, does society respect the role of allied health professionals, such as physiotherapists?

**Content**: Changes made to the content of the KT intervention.[22]<br><br>Types of content tailoring[22]:<br><br>• Adding elements<br>• Removing/skipping elements<br>• Shortening or lengthening the intervention (changing pacing or timing)<br>• Substituting elements<br>• Reordering intervention modules or segments<br>• Repeating intervention modules or segments<br>• Integrating the intervention into another framework<br>• Integrating another treatment into the intervention (e.g., integrating a quality improvement (QI) intervention into a KT intervention)<br>• Others, as applicable to each project	**Context**: Changes made to the way the overall KT intervention is delivered.[22]<br><br>Types of context tailoring[22]:<br><br>• **Format**<br>E.g., an intervention designed to be used in a one-on-one format is now delivered in a group format.<br>• **Setting**<br>E.g., an intervention designed to be used in a mental health clinic is now delivered in primary care.<br>• **Personnel**<br>E.g., an intervention designed to be administered by a family doctor is now delivered by a pharmacist.<br>• **Population**<br>E.g., an intervention developed for an adolescent population is now used for a population in their mid-thirties.

Case example
Tailoring: Mobilization of Vulnerable Elders (MOVE)

Using an online survey tool, the Unit 2A KT intervention development team asked nurses to anonymously share what they thought could make the e-learning modules better for their context. The main suggestions were to provide additional examples that represented case examples from their unit. In addition, younger nurses suggested that the bias that older adults are frail and immobile before hospital admission needed to be addressed in these examples. The nurses also suggested that modules be completed in close succession so that content from previous modules could be recalled more easily. For example complete all modules within six weeks instead of one module every month.

Using a subset of four nurses, the KT intervention development team tested the first two modules from the planned course. The nurses provided suggestions on how to improve the delivery, including ensuring that computer screens are at full brightness, integrating the training with the hospital's existing falls prevention projects, and using examples of patients who were less critically ill than those currently included in the module. The KT intervention development team for Unit 2A explored various elements of tailoring (see table below):

Content[22]	• Adding elements:<br>i. including background information that contains examples of patients who were active before hospital admission to mitigate bias that older adults are frail<br>ii. including case examples of patients who are not as critically ill<br>iii. including case examples of nurses with a range of intersecting categories (e.g., a female nurse who wears a visible religious symbol and speaks with an accent)<br>iv. including explanations of how the practice change activities fit with current hospital practices in falls prevention<br>v. including intersectionality examples (e.g., elderly patient whose son is responsible for making decisions about her care and who does not want his mother to move because of their cultural beliefs)<br>• Pacing — shortening the length of time between the e-learning modules so that they can be completed in a shorter overall time period<br>• Integrating the intervention into another initiative — addressing how this KT project fits with the hospital's current falls prevention activities. Two introduction slides on this were added to the beginning of the module.
Context[22]	• Format — providing audio description for video content<br>• Setting — allow access to modules from remote laptops<br>• Personnel — all members of the intervention team can access the modules

**What to tailor**[22]	
Patient Level[22]	Not applicable. The intervention is directed at the provider level.
Practitioner Level[22]	Based on the notes provided at the beginning of the module, individuals can tailor the screen brightness and duration preference so that they can start/stop when they want.
Organization Level[22]	Coordinate with the IT department to install software to make learning modules accessible on remote laptops on Unit 2A instead of just in the hospital library.<br><br>Plan with the hospital to allocate a room where nurses can complete the modules in a quiet space.
Network Level[22]	Provide extended protected time for professional development to ensure learning is accessible to everyone.
System Level[22]	The **Accessibility for Ontarians with Disabilities Act** requires all Ontario hospitals to make general accessibility provisions. For example, organizations must train staff, volunteers, and contractors on communicating with persons with disabilities.<br><br>In accordance with the Accessibility for Ontarians with Disabilities Act, all text within the module was pre-recorded in an audio format

Activity 4: Tailoring KT interventions for your project

Fill in the blank column to describe the different ways you can tailor the KT intervention selected for your KT project. When doing this, you can keep both intersectional considerations and APEASE considerations in mind. Using an intersectional lens, you can tailor KT interventions to each of the APEASE criteria.[29] Be as specific as possible.

What to tailor? [22] (Keep in mind that you can modify the content or context)		
Patient Level[22]	What can you do to redistribute power?<br>How can you tailor examples to be more inclusive?	
Practitioner Level[22]	How can you support every nurses' participation in the intervention?	
Organization Level[22]	Is there a way that the organization is structured that may benefit certain nurses or patients?	
Network Level[22]	How do social structures impact informal communication systems across the network?	
System Level[22]	How has/does the education system impact knowledge use?<br>What historical considerations must you reflect on?<br>How do societal biases impact knowledge use?	

Consider the APEASE criteria at all levels: **A**ffordability, **P**racticality, **E**ffectiveness and Cost-Effectiveness, **A**cceptability, **S**ide Effects/Safety, **E**quity

Once you have selected and tailored KT strategies:

After you have selected KT intervention strategies that explicitly address barriers and facilitators to change, you can strategically plan how to operationalize these strategies to achieve your desired outome(s).

Before you and your team implement your KT strategies, reflect:

- Did all members of the KT intervention development team have the opportunity to share their thoughts?
- Were members of the group changing their behaviour (e.g., nurses on Unit 2A) involved in selecting and tailoring KT interventions?
- How did the KT intervention development team consider the intesrecting categories of those changing their behaviour?
- How did the KT intervention development team consider systems and structures of power that impact the behaviour change?

Please see **Appendix A** for more information on implementing KT strategies.

For practical resources on project management, see the **Intersectionality Guide**.

Recording and reporting what you implemented

It is important to record what you and your team have done when implementing a KT intervention to enhance transparency and allow others to use what you have learned. Reporting is useful to identify areas for future improvement, replication, and intervention evaluation among others.

In particular, it is important to document and disseminate information about knowledge gaps on underrepresented perspectives. This highlights areas for future research and calls attention to voices not typically represented in KT interventions, such as racialized women.

When recording and reporting the implementation of your KT intervention, you can use the Template for Intervention Description and Replication (TIDieR) framework.[31]

For a guide to using TIDieR, visit **Cotterill et al. (2018) The TIDieR Checklist:** https://tinyurl.com/yyzktlhf[32]

For links to TIDieR in other languages, visit **Hoffman et al. (2014) Better reporting of interventions: template for intervention description and replication (TIDieR) checklist and guide:** https://tinyurl.com/y4a6tfcd[30]

For examples on how other projects have used TIDieR, visit **Cotterill et al. (2018) Examples of different formats which can be used to describe and/or provide study intervention materials:** https://tinyurl.com/y6blcujm[32]

For a video on how TIDieR was developed, visit **Hoffman et al. (2014) Better reporting of interventions: template for intervention description and replication (TIDieR) checklist and guide:** https://tinyurl.com/y6786amu[30]

Appendix A: Implementing KT Interventions

You can use the following table to outline how you will operationalize your implementation strategy:

**Name of implementation strategy**[33]	Audit and Feedback	
**Define implementation strategy**[33]	Emailed reports sent to physicians outlining the number of prescriptions they made for a specific medication	
**Specify it: the actor**[33]<br>• What intersecting categories do you need to be mindful of?	The implementation team coordinator will send out the reports from a team-based email account. She identifies as an early-career researcher and is not in a position of power in relation to the physicians she is emailing.	
**Specify it: the action**[33]<br>• What assumptions are you making about the action?	Sending emailed reports. I am assuming that physicians all use their hospital email account to conduct work and that they access their email only when on-site.	
**Specify it: the action target**[33]<br>• What intersecting categories do you need to be mindful of?	Family physicians. Key intersecting categories include technology literacy, gender identity, and location of training.	
**Temporality**[33]	Reports sent once a week	
**Dose**[33]	Reports contain 3 metrics related to the specific medication	
**Implementation support components required**[33]<br>• Training<br>• Tools<br>• Technical Assistance<br>• Quality Assurance	Training for implementation team coordinator to prepare reports; organizing access to electronic medical records to pull audit data.	
**Implementation outcome affected**[33]	By using Audit and Feedback, I hope to decrease over-prescribing of this medication.	

Appendix B: Project Limitations

We acknowledge that the work of our Canadian Institutes of Health (CIHR)-funded team grant was conducted on unceded lands that were the traditional territories of many people, including the Algonquin, Cree, Dakota, Dene, Huron-Wendat, Mississaugas of the Credit River, and the Musqueam Peoples, and on the homeland of the Métis Nation. We acknowledge the harms of the past and the harms that are ongoing. We are grateful for the generous opportunities to conduct work on these lands.

In 2017, the CIHR launched an opportunity for team grants in gender and KT. This opportunity (sponsored by the Institute of Gender and Health) was developed to recognize that the field of KT had yet to thoughtfully integrate gender into its research agenda. The objectives of the CIHR team grant competition were to generate evidence about whether applying sex- and gender-based analysis to KT interventions involving human participants improves effectiveness, thereby contributing to improved health outcomes; contribute to a broader knowledge base on how to effectively and appropriately integrate gender into KT interventions; and facilitate the consideration and development of gender-transformative approaches in KT interventions.

In response to this call, we submitted a grant aimed at helping KT intervention developers use an intersectional approach when designing and implementing interventions to address the needs of older adults. We received feedback from the CIHR peer review committee that substantial concern was raised about our focus on intersectionality. In particular, the Scientific Officer's notes described that the focus on intersectionality would dilute the focus on gender and needed to be reconsidered. A meeting was subsequently held with the successfully funded team and this issue was raised again. We acknowledge the limitation that our intersectional approach comes at the expense of a minimized focus on gender. However, because intersecting categories, such as gender and age, are experienced together, we ultimately elected to use an intersectional approach as it encapsulates the lived experience of those we aim to impact.

A more significant limitation of our work is that we did not include First Nations, Inuit, and Métis community members in the grant proposal. As such, their needs and perspectives were not included in the research grant and, consequently, funded activities. Our team did not have established relationships or expertise in this area and as such, we felt it was inappropriate for our team to work on a grant in this area.

We strongly believe that consideration of gender and KT for Indigenous peoples should be a primary focus of a distinct team grant.

There are established best practices for community engagement with First Nations, Inuit, and Métis Peoples that begin with principles of collaboration, which take time to develop and must not be tokenistic. The principles for collaboration should ensure authentic engagement, shared respect, trust, and commitment to ensure long-term, mutually empowered relationships. These principles should also ensure that the research-related priorities meet the needs, perspectives, and expectations of the First Nations, Inuit, and Métis Peoples. Indigenous peoples have a long history of conducting research, and this tradition continues today with many Indigenous healers and scholars leading research in various areas. Indeed, there are many Indigenous scholars working in the KT field.

Because the team's work did not include First Nations, Inuit, and Métis Peoples and involve adhering to the principles that guide their engagement in research, the needs and considerations of these Peoples were not included in the work conducted in this team grant. As such, anyone who is considering using the outputs of this team grant needs to know that **they cannot be broadly applied to these Peoples and there may be other more culturally appropriate models/theories/frameworks that are useful to consider.** Similarly, because this research focused on older adults (and in particular, chronic disease management in older adults) **it does not apply to children and youth**.

We believe that any KT intervention work needs to begin with engaging the appropriate community and is only applicable when those communities are engaged throughout the research enterprise. Moreover, intersectionality involves deep immersion in the lived experiences and priorities of those communities. As a result, KT work requires immersive work with various populations and not just key informants to ensure the work meets the needs of the relevant populations.

We thank and acknowledge Dr. Lisa Richardson, Co-Lead, Indigenous Health Education, Faculty of Medicine, University of Toronto, for her time and expertise in reviewing this statement.

References

1. Government of Canada. Knowledge Translation in Health Care: Moving from Evidence to Practice - CIHR. 2010. http://www.cihr-irsc.gc.ca/e/40618.html. Accessed May 28, 2019.
2. Tannenbaum, C., Greaves, L., & Graham, I.D. Why sex and gender matter in implementation research. BMC Med Res Methodol 2016;16:145. doi: 10.1186/s12874-016-0247-7
3. City of Ottawa and City for All Women Initiative. Advancing Equity and Inclusion – A Guide for Municipalities. CAWI. 2015. http://www.cawi-ivtf.org/sites/default/files/publications/advancing-equity-inclusion-web.pdf. Accessed February 6, 2019.
4. Public Health Agency of Canada. Key Health Inequalities in Canada: A National Portrait – Executive Summary. 2018. https://www.canada.ca/en/public-health/services/publications/science-research-data/key-health-inequalities-canada-national-portrait-executive-summary.html. Accessed February 6, 2019.
5. The Association for Women's Rights in Development. Intersectionality: A Tool for Gender and Economic Justice. Women's Rights and Economic Change. https://lgbtq.unc.edu/sites/lgbtq.unc.edu/files/documents/intersectionality_en.pdf. Published August 2004. Accessed February 4, 2019.
6. Graham ID, Logan J, Harrison MB. Straus SE, Tetroe J, Caswell W, Robinson N. Lost in Knowledge Translation: Time For A Map? J Contin Educ Health Prof 2006; 26(1): 13.
7. MOVES. The MOVE Program. https://www.movescanada.ca/. Accessed May 2, 2019.
8. Collins, P. H. Black feminist thought: knowledge, consciousness, and the politics of empowerment. Routledge. 1990. https://trove.nla.gov.au/version/21207078
9. Crenshaw, K. Demarginalizing the intersection of race and sex: A black feminist critique of antidiscrimination doctrine, feminist theory and antiracist politics. U. Chi. Legal F. 1989;139.
10. Crenshaw, K. A Black feminist critique of antidiscrimination law and politics. The politics of law: A progressive critique. 1990;195.
11. Crenshaw, K. Mapping the Margins: Intersectionality, Identity Politics, and Violence against Women of Color. Stanford Law Review. 1991;43(6), 1241-1299. doi:10.2307/1229039
12. The Learning Network. Issue 15: Intersectionality. 2015. http://www.vawlearningnetwork.ca/sites/vawlearningnetwork.ca/files/Intersectioanlity_Newsletter_FINAL2.pdf. Accessed February 7, 2019.
13. Hankivsky, O. Intersectionality 101. The Institute for Intersectionality Research & Policy, SFU. 2014;1-34. http://vawforum-cwr.ca/sites/default/files/attachments/intersectionallity_101.pdf. Accessed February 7, 2019.
14. Hankivsky O, Grace D, Hunting G, et al. An intersectionality-based policy analysis framework: critical reflections on a methodology for advancing equity. International Journal for Equity in Health. 2014;13:119. doi:10.1186/s12939-014-0119-x.
15. PROGRESS-Plus. Cochrane Equity Methods. https://methods.cochrane.org/equity/projects/evidence-equity/progress-plus. Accessed November 12, 2019.
16. O'Neill, J., Tabish, H., et al. Applying an equity lens to interventions: Using PROGRESS ensures consideration of socially stratifying factors to illuminate inequities in health. Journal of Clinical Epidemiology. 2014;67:56-64.
17. Office of Diversity and Outreach. Unconscious Bias. UCSF. N.d. https://diversity.ucsf.edu/resources/unconscious-bias. Accessed May 7, 2019.
18. Harvard EDU. Project Implicit. N.d. from https://implicit.harvard.edu/implicit/. Accessed March 7, 2019.
19. Government of Canada, I. C. Canada Research Chairs. 2018. http://www.chairs-chaires.gc.ca/program-programme/equity-equite/bias/module-eng.aspx?pedisable=false. Accessed March 7, 2019.
20. EdX. Unconscious Bias: From Awareness to Action. 2017. https://www.edx.org/course/unconscious-bias-awareness-action-catalystx-ub1x. Accessed March 7, 2019.
21. Moore, J.E., Mascarenhas, A., Bain, J. et al. Developing a comprehensive definition of sustainability. Implementation Sci 2017;12:110. https://doi.org/10.1186/s13012-017-0637-1
22. Stirman, SW, Baumann SW, & Miller CJ. The FRAME: an expanded framework for reporting adaptations and modifications to evidence-based interventions. Implement Sci. 2019;14:58. https://doi.org/10.1186/s13012-019-0898-y
23. Atkins L. The Behaviour Change Wheel: a tool to promote consumer food safety. Presentation at: CFSE Conference; January 2017; Washington, DC. http://www.fightbac.org/wp-content/uploads/2017/01/CFSE_Lou-Atkins_Jan-17.pdf. Accessed May 2, 2019.
24. Michie, S., Atkins, L., & West, R. The behaviour change wheel. A guide to designing interventions. 1st ed. Great Britain: Silverback Publishing, 2014;1003-1010.
25. Cane J, O'Connor D, & Michie S. Validation of the theoretical domains framework for use in behaviour change and implementation research. Implement Sci. 2012;7(1):37. doi:10.1186/1748-5908-7-37.
26. Michie S, van Stralen MM, West R. The behaviour change wheel: a new method for characterising and designing behaviour change interventions. Implement Sci. 2011;6:42. Published 2011 Apr 23. doi:10.1186/1748-5908-6-42
27. Glasgow, R.E., Vogt, T.M., & Boles, S M. Evaluating the public health impact of health promotion interventions: the RE-AIM framework, American Journal of Public Health 1999;89(9):1322-1327.
28. McMillan SS, King M, Tully MP. How to use the nominal group and Delphi techniques. Int J Clin Pharm. 2016;38(3):655–662. doi:10.1007/s11096-016-0257-x
29. Atkins L. Using the Behaviour Change Wheel in infection prevention and control practice. J Infect Prev. 2016;17(2):74–78. doi:10.1177/1757177415615952
30. Hoffmann TC, Glasziou PP, Boutron I, Milne R, Perera R, Moher D, Altman DG, Barbour V, Macdonald H, Johnston M, et al. Better reporting of interventions: template for intervention description and replication (TIDieR) checklist and guide. BMJ. 2014;348:g1687.
31. Lundgren L: Modifications of evidence-based practices in community-based addiction treatment organizations: a qualitative research study. Addict Behav. 2011, 36 (6): 630-635. 10.1016/j.addbeh.2011.01.003.
32. Cotterill, S., Knowles, S., Martindale, A.-M., Elvey, R., Howard, S., Coupe, N., et al. Getting messier with TIDieR: embracing context and complexity in intervention reporting. BMC Medical Research Methodology, 2018;18(1):12. https://doi.org/10.1186/s12874-017-0461-y
33. Proctor, E.K., Powell, B.J., & McMillen, J.C. Implementation strategies: recommendations for specifying and reporting. Implement Sci. 2013;8(139). https://implementationscience.biomedcentral.com/articles/10.1186/1748-5908-8-139
